# Metal Halide Perovskite/Electrode Contacts in Charge‐Transporting‐Layer‐Free Devices

**DOI:** 10.1002/advs.202203683

**Published:** 2022-11-01

**Authors:** Deli Li, Xue Dong, Peng Cheng, Lin Song, Zhongbin Wu, Yonghua Chen, Wei Huang

**Affiliations:** ^1^ Frontiers Science Center for Flexible Electronics Xi'an Institute of Flexible Electronics (IFE) and Xi'an Institute of Biomedical Materials and Engineering Northwestern Polytechnical University 127 West Youyi Road Xi'an 710072 P. R. China; ^2^ Fujian cross Strait Institute of Flexible Electronics (Future Technologies) Fujian Normal University Fuzhou 350117 P. R. China; ^3^ Key Laboratory of Flexible Electronics (KLoFE) and Institute of Advanced Materials (IAM) Nanjing Tech University 30 South Puzhu Road Nanjing Jiangsu 211816 P. R. China; ^4^ Key Laboratory for Organic Electronics and Information Displays and Institute of Advanced Materials Nanjing University of Posts and Telecommunications Nanjing 210023 P. R. China

**Keywords:** artificial synapses, charge transporting layer, perovskite/electrode contact, solar cells, transistors

## Abstract

Metal halide perovskites have drawn substantial interest in optoelectronic devices in the past decade. Perovskite/electrode contacts are crucial for constructing high‐performance charge‐transporting‐layer‐free perovskite devices, such as solar cells, field‐effect transistors, artificial synapses, memories, etc. Many studies have evidenced that the perovskite layer can directly contact the electrodes, showing abundant physicochemical, electronic, and photoelectric properties in charge‐transporting‐layer‐free perovskite devices. Meanwhile, for perovskite/metal contacts, some critical interfacial physical and chemical processes are reported, including band bending, interface dipoles, metal halogenation, and perovskite decomposition induced by metal electrodes. Thus, a systematic summary of the role of metal halide perovskite/electrode contacts on device performance is essential. This review summarizes and discusses charge carrier dynamics, electronic band engineering, electrode corrosion, electrochemical metallization and dissolution, perovskite decomposition, and interface engineering in perovskite/electrode contacts‐based electronic devices for a comprehensive understanding of the contacts. The physicochemical, electronic, and morphological properties of various perovskite/electrode contacts, as well as relevant engineering techniques, are presented. Finally, the current challenges are analyzed, and appropriate recommendations are put forward. It can be expected that further research will lead to significant breakthroughs in their application and promote reforms and innovations in future solid‐state physics and materials science.

## Graphical abstract

Metal halide perovskite (MHP)/electrode contact is central in device physics, fabrications, and modification of many MHP‐based devices. Outstanding achievements in MHP interface science are overviewed, interface and electrode modification principles and techniques are introduced, interface chemistry is summarized, and the roadmap for further development of the topic is described.

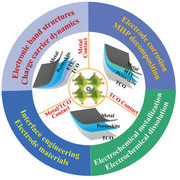

## 1 Introduction

During the past decade, metal halide perovskite (MHP) semiconductors have drawn tremendous attention due to their high intrinsic charge‐carrier mobility,^[^ ^1^ ^]^ ambipolar‐transporting property,^[^ ^2^ ^]^ ion transporting characteristic,^[^ ^3^ ^]^ high absorption coefficient with the magnitude of 10^4^–10^5^ cm^−1^,^[^ ^4^ ^]^ and high photon‐to‐electron conversion yield.^[^ ^5^ ^]^ Based on these, perovskite solar cells (PSCs),^[^ ^6^, ^7^, ^8^ ^]^ perovskite light‐emitting diodes,^[^ ^9^, ^10^, ^11^ ^]^ and perovskite photodetectors^[^ ^12^, ^13^ ^]^ have been developed, showing great potential in flexible electronic devices. Recently, charge‐transporting‐layer‐free perovskite devices, for example, electron/hole‐transporting‐layer free PSCs, artificial synapses,^[^ ^14^ ^]^ field‐effect transistors,^[^ ^15^, ^16^, ^17^, ^18^ ^]^ memories,^[^ ^19^, ^20^ ^]^ and memristors,^[^ ^21^ ^]^ have been realized to achieve new optoelectrical functions. These simplified devices are prepared without the electron‐transporting layer (ETL) or hole‐transporting layer (HTL), leading to the formation of MHP layer/electrode contacts in the device configuration. Metal electrodes (Au, Al, Ag, or Cu) can be deposited directly on the MHP layer by vacuum‐evaporation when preparing these electronic devices, which is beneficial for the future low‐cost and simplified massive manufacture. Impressively, the tremendous material and device progress about perovskite/electrode contacts not only broadens the new application of MHP materials but also provides an opportunity for further understanding of relevant device physics.

MHP/electrode contacts are important heterojunction structures in simplified‐structure PSCs, MHP‐based artificial synapses, memristors, and field‐effect transistors. In general, the simplified‐structure PSCs (ETL/HTL‐free PSCs) were designed by eliminating the ETL or HTL in conventional PSCs,^[^ ^22^ ^]^ where ETL and HTL were used initially to isolate MHP from the electrodes. The MHP layer is directly hinged with transparent conducting oxide (TCO) electrode, metal electrode, or conductive carbon electrode. Thus, understanding and regulating the MHP/electrode contacts become vital to improving the performance of ETL‐free, HTL‐free, transporting‐layer‐free (TL‐free) PSCs with mature material preparation technology. It is believed that simplified‐structure PSCs can reduce the cost and the preparation complexity while maintaining high device performances.^[^ ^23^, ^24^, ^25^ ^]^ It should be mentioned that MHP‐based artificial synapses and field‐effect transistors are considered the next generation of electric devices for massive data storage and processing,^[^ ^14^, ^21^ ^]^ together with the background of Big Data processing techniques. Besides, MHP/electrode contacts exhibit their importance in MHP‐based memristors because of the resistive switching memory behavior and the device configuration with a metal–insulator–metal, such as the combination of Au/MHP/indium tin oxide (ITO).

In addition to the excellent semiconductor characteristics of MHP, MHP/electrode contacts are crucial for constructing high‐performance charge‐transporting‐layer‐free devices, such as solar cells, field‐effect transistors, artificial synapses, memories, and so on. For MHP/metal contacts, some critical interfacial physical and chemical processes have been reported, including band bending, interface dipoles, metal halogenation, and the MHP decomposition induced by metal electrodes. Many studies have focused on the energy levels,^[^ ^25^ ^]^ band bending,^[^ ^26^ ^]^ charge transfer, charge carrier recombination,^[^ ^27^ ^]^ corrosion of electrodes,^[^ ^28^ ^]^ decomposition of MHP,^[^ ^29^ ^]^ various modifications of the electrode, and their application in electronic devices,^[^ ^13^ ^]^ indicating abundant physicochemical, electronic, and photoelectric processes at the MHP/electrode contacts. However, the core working mechanism of many devices that are closely related to the interface is still unclear, for instance, the origin of open‐circuit voltage (*V*_OC_) of ETL/HTL‐free PSCs, the mechanism of resistance switching effect of MHP‐based memristors, and the reasons that spoil the on/off ratio of MHP‐based field‐effect transistors. Answers to the above questions count for much to determine the performance potential of the devices quantitatively and to identify the main physical processes that limit the performance of the devices. Moreover, interface engineering of the MHP/electrode contact, highlighted as an essential way to improve the device's performance with MHP/electrode contacts, has been widely used in device modification. Thus, elucidation of the physics and chemicals, interfacial electronic structures, and carrier behaviors of MHP/metal junctions are the basis for understanding and optimizing the performance of these devices.

**Figure 1:**
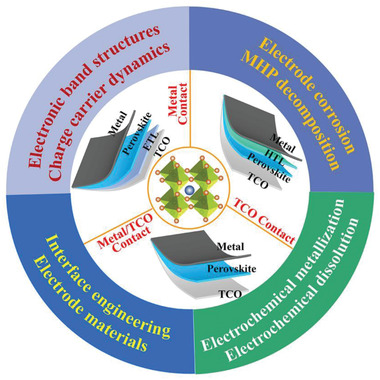
The related MHP/electrode contacts involve physics, chemistry, and engineering.

In this review, a systematic summary of the role of MHP/electrode contacts on the device's performance is made. The charge‐transporting‐layer‐free devices with the MHP/electrodes, including the ETL/HTL‐free PSCs, field‐effect transistors, and artificial synapses, are summarized and discussed with a particular emphasis on the effect of the special contacts on the device physics. Then, the understanding of band structure, charge carrier processes, interface chemistries, and problems of the MHP/TCO contacts and the MHP/metal contacts are presented, respectively, focusing on the electrode engineering technologies in the device modification. As shown in **Figure** **1**, the charge carrier dynamics, electronic band engineering, electrode corrosion, electrochemical metallization and dissolution, MHP decomposition, and interface engineering in MHP/electrode contacts‐based electronic devices are summarized and discussed for a comprehensive understanding of the contacts. The physicochemical, electronic, and morphological properties of various MHP/electrode contacts and relevant engineering techniques, are presented. In addition, an accurate model is helpful for understanding the device's operation and optimizing the device's performance. Here, the modeling of the ETL/HTL‐free PSCs and MHP‐based memristor devices are also discussed. Finally, the current challenges are analyzed, and relevant recommendations are put forward to improve the device's performance.

## 2 Metal Halide Perovskite

**Figure 2:**
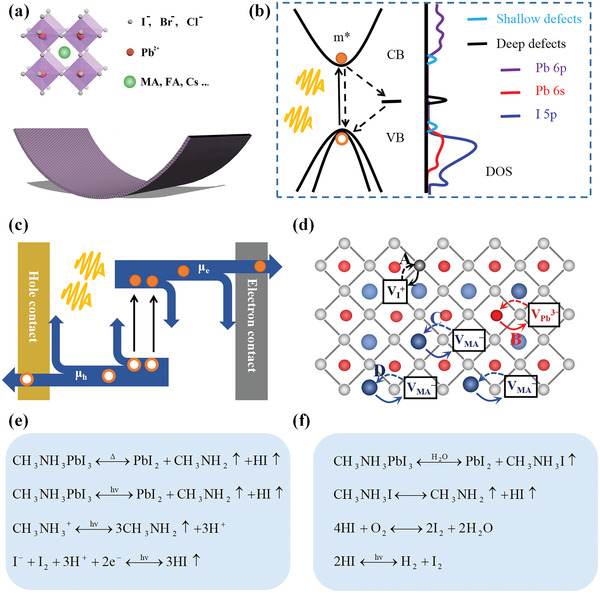
a) Cubic MHP structure of ABX_3_ and flexible perovskite films. b) Schematics illustration of the band structures of MAPbI_3_ at *Pm3m*, and density of the trap states (*n*_t_) with trap states close to the conduction band (CB) and the valence band (VB). c) Schematics illustration of the carrier dynamics in MHP semiconductors. d) Schematic illustration of the three ionic transport mechanisms involving conventional vacancy hopping between neighboring positions. e) The possible mechanism of MAPbI_3_ decomposition on exposure to light and heat. f) The possible mechanism of MAPbI_3_ decomposition on exposure to sunlight and water. Reproduced with permission.^[^ ^43^ ^]^ Copyright 2017, Wiley‐VCH.

MHP is a class of ABX_3_ compounds, where A, and B are cations and X is a halide anion (I^−^, Br^−^, Cl^−^). As shown in **Figure** **2**a, the MHP has a crystal structure of a cubic array of corner‐sharing [BX_6_] octahedra and cuboctahedral cages occupied by an A cation. The B‐site is occupied by a divalent cation (e.g., Pb^2+^, Sn^2+^, Ge^2+^).^[^ ^2^ ^]^ In 1978, Weber formed the MHP semiconductor by introducing methylamine cations (MA^+^) into the crystal structure.^[^ ^30^ ^]^ Later, formamidinium cations (FA^+^) were introduced into ABX_3_ compounds, leading to various high‐performance optoelectronic devices.^[^ ^1^ ^]^ The introduction of organic cations can improve the solubility and film‐forming properties of the materials, so that they can be prepared by solution‐based methods, such as spin‐coating,^[^ ^31^ ^]^ slot‐die coating,^[^ ^32^ ^]^ and blade‐coating.^[^ ^33^ ^]^ When a single cation occupies the A site (e.g., Cs^+^, Rb^+^, K^+^), all‐inorganic MHPs are formed, which have been widely investigated by researchers due to their excellent electronic and optoelectronic properties and high thermal stability.^[^ ^34^ ^]^

The chemical bonds of B‐X in the ABX_3_ structures determine the mechanical flexibility of MHP materials. The flexibility of the MHP is calibrated by the ratio of bulk modulus to shear modulus and Poisson's ratio.^[^ ^35^, ^36^, ^37^ ^]^ Feng calculated the ratio of bulk modulus to shear modulus and Poisson's ratio with results of over 20.0 and 0.26, respectively.^[^ ^38^ ^]^ These results indicated that MHP materials could endure compression, tortuosity, and bending to some extent, and the mechanical property is beneficial to fabricating MHP‐based flexible electronic devices.

MHP semiconductors exhibit numerical unique electrical and optical properties: high carrier mobility, ambipolar charge transportation, high optical absorption, relatively low trap density, and a steep absorption edge. The unique electrical and optical properties are related to the band structure and the density of state of the MHP materials. Based on the first‐principles calculations, Yin et al. presented a systematic theoretical study on two representative MHPs, such as MAPbI_3_ and CsSnI_3_.^[^ ^39^ ^]^ For example, Figure 2b shows the band structures of MAPbI_3_ with a cubic crystal structure. The calculation reveals that the lower parts of the conduction bands are mainly derived from the unoccupied Pb p orbitals. The upper valence bands are mostly halogen p orbitals mixed with a small component of Pb s orbitals, which makes the p–p transitions possible.^[^ ^39^ ^]^ The band structure also shows that the MHP materials are a kind of direct bandgap semiconductor, that is, the valence band maximum and conduction band minimum occur at the same K point. The realization of the p–p transition and the direct bandgap enable strong optical transitions of the MHP. Moreover, the band structure indicates small effective masses for both electrons and holes, which is thought to be the reason for the high carrier mobility and ambipolar charge‐transporting characteristics.

The traps and the trap density of the MHP semiconductor were usually revealed by the absorption spectra of polycrystalline and crystalline MHP films.^[^ ^40^ ^]^ Although MHP films have a higher density of defects, grain boundaries, and interfaces, the dominant traps are shallow with a low density, around 10^10^ cm^−3^ for MHP single crystals. Figure 2b also presents the density of the shallow trap states (*n*_t_) within the bandgap of the MHP semiconductor, in which the trap states are identified close to the conduction band and valence band. The first‐principles calculations show that all point defects with low formation energies, such as iodine interstitials, MA interstitials, MA vacancies, Pb vacancies, and iodine vacancies, can only generate shallow traps.^[^ ^5^ ^]^

Compared with shallow traps, deep traps in the bandgaps have more effects on the performance of MHP‐based optoelectronic devices, which can act as Shockley–Read–Hall nonradiative recombination centers. The deep defects, such as iodine atoms at MA sites, Pb interstitials, have relatively high formation energies, requiring a specific chemical environment. The surface passivation method, for example, the passivation of the MHP surface by [6,6]‐phenyl‐C_61_‐butyric acid methyl ester (PCBM), can reduce the nonradiative recombination centers, indicating that exposed faces may result in deep traps.^[^ ^41^ ^]^ Besides, chemical or physical processes at the interface between MHP and electrode or transport layer may affect the composition of the film and induce the formation of deep defect states.^[^ ^42^ ^]^

As shown in Figure 2c, charge carrier dynamics consist of the charge‐carrier generation, transportation, and recombination in the bulk of MHP semiconductors.^[^ ^1^ ^]^ First, the high efficiency of free charge‐carrier generation under light is enabled by a high absorption coefficient and low exciton binding energy. MHPs are among the materials with the highest absorption coefficient (≈10^5^ cm^−1^), so a 300‐nm‐thick MHP layer is enough to capture nearly 100% of the absorbable light. Magnetoabsorption measurements were used to directly determine the exciton binding energy, yielding a value of 12 meV, indicating that the charge carrier generation process is exciton‐free at room temperature.^[^ ^44^ ^]^ Second, MHPs mainly have two unique intrinsic transport aspects: large charge carrier mobilities and ambipolar properties. It is reported that both the electron and hole mobility could achieve 20 cm^2^ V^−1^ s^−1^,^[^ ^45^ ^]^ yielding considerable electron/hole diffusion lengths and efficient charge carrier extraction. The ambipolar conductivity characteristics in MHP‐based devices, such as the field‐effect transistors and PSCs, originate from the balanced electron and hole effective masses, showing great potential in simplifying the device design and device processing.^[^ ^2^ ^]^ As for the external factors, it is worth noting that the interfaces of MHPs with other components and the ion screening significantly affect the carrier transport in practical MHP‐based devices.^[^ ^3^ ^]^ However, the dependence of mobility on the temperature, electric field, and magnetic field is still controversial because the performance of MHP‐based field‐effect transistors is limited by ion shielding and interface effects, which may not get an accurate measurement of these factors. Third, charge carrier recombination has been widely studied because it is directly connected to the reduction of the performance of PSCs. Charge carrier recombination includes radiative recombination, nonradiative recombination in bulk and at the interfaces, and Auger recombination. The radiative recombination and Auger non‐recombination belong to intrinsic recombination processes. The radiative recombination is inevitably related to the well‐known Shockley–Queisser limit, the power conversion efficiency (PCE) limit of single‐junction solar cells.^[^ ^46^ ^]^ It was reported that the Auger nonradiative is much weaker in MAPbI_3_, which has a negligible influence on the performance of most PSCs.^[^ ^47^ ^]^ As for nonradiative recombination, surface recombination is more critical than bulk recombination within MHP films.^[^ ^5^, ^41^, ^47^ ^]^ Thus, minimizing the nonradiative recombination at the interface of MHP contact in PSCs is the key to improving the device's performance.

MHPs are mixed ionic–electronic semiconductors, as suggested by many computational and experimental studies.^[^ ^37^, ^48^ ^]^ Ion migration refers to the drift and diffusion of intrinsic ionic defects in semiconductors or electrolytes. The mobile ionic species in the MHP are associated with, for example, I^−^, Pb^2+^, and MA^+^ vacancies in MAPbI_3_, which have equilibrium concentration at room temperature. By performing transition‐state calculations, Eames et al. calculated the migration activation energies for three mechanisms of ion migration, as shown in Figure 2d.^[^ ^3^ ^]^ The lowest activation energy of 0.58 eV was obtained for I^−^ migrating along the edge of the [PbI_6_] octahedron, indicating that I^−^ is the most easily mobile ion. However, the experimental results show inconsistency, which confirmed that MA^+^ was also the migrating species. Yuan and Huang attributed the inconsistency to calculated activation energies that could be responsible for the ion migration in a bulk crystal. By contrast, the ion migration at grain boundaries might dominate the measured ionic conductivity.^[^ ^49^ ^]^ The formation of the PbI_2_ phase was observed under the applied electric field, which was explained by the massive migration of both MA^+^ ions and I^−^ ions.^[^ ^50^ ^]^ Mixed ionic–electronic processes are ubiquitous in MHP‐based devices. The anomalous photocurrent density–voltage (*J*–*V*) hysteresis in PSCs is an extensively studied issue in which ion migration has a vital contribution.^[^ ^51^, ^52^ ^]^ Another important mixed ionic–electronic effect is the giant switchable photovoltaic effect discovered by Xiao et al., indicating that a flipped ion distribution caused by the polarized applied bias voltage produced a direction‐flipped photocurrent in a planar MHP heterojunction.^[^ ^53^ ^]^ Ion accumulation at the MHP/electrode contact with applied voltage dynamically adjusts the charge carrier injection, which is considered to be related to the resistance switching effect that constitutes the basis of the working principle of the MHP‐based memristors. As for the MHP‐based field‐effect transistors, ion migration and the consequent ion accumulation at the MHP/metal contacts (the ion screening effect) are the main hindrances to improving device performances.^[^ ^54^, ^55^ ^]^

It has been evidenced that MHP can decompose under humidity, heat, light, and oxygen.^[^ ^43^, ^56^, ^57^, ^58^, ^59^ ^]^ The photodecomposition, thermal decomposition, and moisture decomposition of MHP introduce excessive organic iodides (MAI or FAI) and various inorganics (e.g., HI or NH_4_Cl) in the films, leading to the corrosion of electrodes and instability in MHP devices. MAPbI_3_ appears two reversible decomposition reactions: (1) CH_3_NH_2_ + HI, (2) Pb + I_2_, and one irreversible decomposition reaction: NH_3_ + CH_3_I.^[^ ^60^ ^]^ Figure 2e shows the possible decomposition mechanism on exposure to light and heat. On exposure to light or heat, I^−^ is oxidized to iodine by losing an electron, which deconstructs the MHP crystal structure. Meanwhile, the methylammonium ion loses a proton, and the iodine acquires the electron and the proton, resulting in methylamine gas and HI gas.^[^ ^43^ ^]^ Figure 2f shows the possible mechanisms of MHP decomposition in moisture. As demonstrated by Walsh and co‐workers, the proton of ammonium in MAPbI_3_ will first coordinate with H_2_O to form an intermediate of [(CH_3_NH_3_^+^)*_n_*_‐1_(CH_3_NH_2_)*_n_* PbI_3_][H_3_O].^[^ ^37^ ^]^ Then, the intermediate decomposes into HI, CH_3_NH_2_, and solid PbI_2_, and the generated HI will subsequently generate I_2_, H_2_O, and H_2_ in the case of oxygen and heating.

## 3 Electronic Devices with MHP/Electrode Contacts

The excellent optoelectronic properties of room temperature solution‐processability and flexibility make MHPs highly attractive for superior performance in electronic devices with simplified structures and low‐cost preparation. PSCs without ETL or HTL have been proved feasible because of the ambipolar characteristics and long carrier diffusion length. In addition to PSCs, the interaction between metal electrodes and the MHP layer yields some new electrical or optoelectrical characteristics of MHP‐based diodes, showing novel application directions of MHP materials in the electronic and optoelectronic devices. Thus, an overall review of the devices with MHP/electrode contacts could help the studies of the MHP‐based devices and the development of new concepts for device structure and geometry engineering.

### 3.1 ETL/HTL‐Free PSCs

#### 3.1.1 Structures of the ETL/HTL‐Free PSCs

Most reported PSCs are based on a stacked architecture, primarily consisting of a TCO, an MHP layer, a hole transport layer, an electron transport layer, and a top metal electrode.^[^ ^8^, ^61^, ^62^, ^63^, ^64^ ^]^ Among them, the charge‐transporting layers should meet the requirements of high light transmittance, excellent charge transfer, strict energy level matching, and certain chemical inertness to the metal electrode and MHP materials. At the same time, the fabrication process should be compatible with the process of the MHP layer. As a consequence, ideal charge‐transporting layer materials are challenging to obtain.

**Figure 3:**
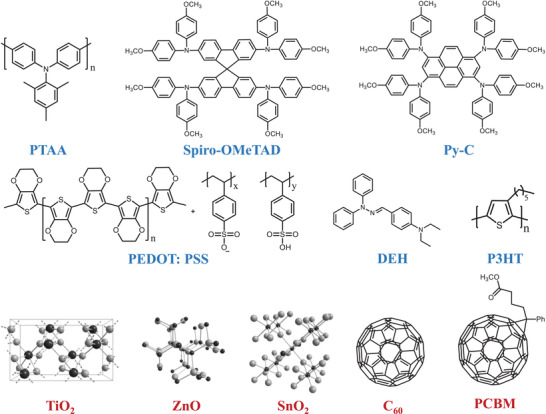
Common chemical structures of the hole transporting layer (blue) and electron transporting layer (red).

**Figure** **3** exhibits the chemical structures of the commonly used hole transporting and electron transporting materials. Metal oxide materials such as TiO_2_, SnO_2_, and ZnO require extremely high annealing temperatures (>450 °C) to ensure an excellent electron transport performance, inevitably increasing the industrial production cost in preparation.^[^ ^65^, ^66^ ^]^ Organic materials including hole transport materials (HTMs), like poly[bis(4‐phenyl)(2,4,6‐trimethylphenyl)amine] (PTAA), *N*′, *N* ′‐octakis(4‐methoxyphenyl)‐9,9′‐spirobi[9H‐fluorene]‐2,2′,7,7′‐tetramine (Spiro‐OMeTAD), poly(3,4‐ethylenedioxythiophene):poly(styrenesulfonate) (PEDOT‐PSS), etc., and electron transport materials (ETMs) like fullerene (C_60_), fullerene derivatives, etc. have been proven to possess effective charge transporting property. But compared with cheap MHP materials, these materials are usually expensive, especially for state‐of‐the‐art HTMs.^[^ ^59^, ^67^ ^]^ Besides, some HTL and ETL materials may result in physical and chemical instability issues in PSCs devices, for instance, the ion migration through the transport layers to metal electrodes, the diffusion of electrode metal atoms through the transport layers to the MHP layer, chemical reactions between the transport materials and MHP layer, accelerating the degradation of PSC devices.^[^ ^68^, ^69^ ^]^

Many kinds of ETL/HTL‐free PSCs are developed to simplify the complicated multilayer device architecture and further reduce manufacturing costs. **Table** **1** summarizes the configurations and photovoltaic parameters of the transporting layer‐free PSCs reported to date. As shown, the ETL‐free or HTL‐free PSCs have achieved peak PCEs of above 20%, boosting intensive research on device geometry design, optimization of thin‐film, interface engineering, and device physics. In addition, the PCE of TL‐free PSCs has been reported with a value exceeding 18%, indicating the prospects for further simplifying of the device structure.^[^ ^42^ ^]^ There are also several reviews on PSCs with emphasis on the progress of the performance and working mechanism of ETL/HTL‐free PSCs.^[^ ^24^, ^70^, ^71^ ^]^

**Table 1:** Summary of device structures and performance parameters of the examples of the reported ETL/HTL‐free PSCs with and without modification of the MHP/electrode contact

Type	Device structure	*V*_OC_ [V]	*J*_SC_ [mA cm^−2^]	FF	PCE [%]	Refs.
Mesoporous HTL‐free PSCs	Au/MAPbI_3_/m‐TiO_2_/FTO	0.809	4.85	65.38	2.56	[82]
	Au/MAPbI_3_/m‐TiO_2_/FTO	0.84	19	68	10.85	[90]
	Ag/MAPbI_3_/m‐TiO_2_/FTO	0.087	4.93	39.34	0.17	[82]
	Pt/MAPbI_3_/m‐TiO_2_/FTO	0.686	7.17	62.6	3.08	[82]
	Ni/MAPbI_3_/m‐TiO_2_/FTO	0.411	8.52	49.92	1.75	[82]
	Cu/MAPbI_3_/m‐TiO_2_/FTO	0.301	10.42	33.69	1.06	[82]
	Cr/MAPbI_3_/m‐TiO_2_/FTO	0.753	0.89	21.83	0.14	[82]
	C/MAPbI_3_/m‐TiO_2_/c‐TiO_2_/FTO	0.84	14.70	60	7.41	[70]
	C/MAPbI_3_/SiO_2_/m‐TiO_2_/FTO	0.993	21.43	66.23	14.09	[91]
	C/MAPbI_3_/m‐Al_2_O_3_/m‐TiO_2_/c‐TiO_2_/FTO	1.00	19.28	59	12.1	[92]
	C/MAPbI_3_/m‐ZrO/m‐TiO_2_/c‐TiO_2_/FTO	1.002	–	–	12.6	[93]
	C/MAPbI_3_/m‐Al_2_O_3_/m‐TiO_2_/c‐TiO_2_/FTO	0.95	19.50	70	13.0	[94]
	C/MAPbI_3_(SrCl_2_)_0.1_/m‐Al_2_O_3_/c‐TiO_2_/m‐TiO_2_/FTO	1.05	20.18	75	15.9	[94]
	C:SWCNTs/MAPbI_3_/m‐Al_2_O_3_/m‐TiO_2_/c‐TiO_2_/FTO	1.01	21.26	69	14.7	[78]
N–i–p ETL‐free PSCs	FTO/MAPbI_3‐_ *_x_* Cl*_x_*/Spiro‐OMeTAD/Au	1.06	19.76	0.67	14.04	[22]
	FTO/Cs_0.05_FA_0.81_MA_0.14_PbI_2.55_Br_0.45_/Spiro‐OMeTAD/Au	1.07	20.99	0.68	15.4	[95]
	FTO/MAPbI_3_/Spiro‐OMeTAD/Au	1.07	20.4	0.69	15.1	[70]
	FTO/MAPbI_3_/Spiro‐OMeTAD/Au	1.127	22.45	0.75	18.20	[96]
	E‐FTO/MAPbI_3_/PMMA/Au	1.123	22.81	0.75	19.22	[97]
	FTO/MAPbI_3_/Spiro‐OMeTAD(Cu/Ag nanoparticles)/Au	1.124	22.96	0.732	18.89	[70]
	FTO/maze‐like MAPbI_3_/Spiro‐OMeTAD/Au	1.08	22.2	0.77	18.5	[98]
	FTO/MAPbI_3−_ *_x_* Cl*_x_*/spiro/Ag	0.96	18.0	64.08	11.07	[99]
	FTO/MAPbI_3−_ *_x_* Cl*_x_*/spiro/Ag	0.83	18.8	47.3	12.7	[100]
	ITO/MAPbI_3_/Spiro‐OMeTAD/Au	1.061	23.61	0.779	19.52	[27]
N–i–p ETL‐free PSCs with ITO modified	ITO/Cs_2_CO_3_/MAPbI_3_/Spiro‐OMeTAD/Au	1.07	19.9	0.71	15.1	[101]
	FTO/TMAH/MAPbI_3_/Spiro‐OMeTAD/Au	1.17	23.22	0.73	20.1	[74]
	ITO/TAPC/MAPbI_3_/Spiro‐OMeTAD/Au	1.12	22.07	0.78	19.4	[25]
	ITO/MSAPBS/(FAPbI_3_)_0.85_(MAPbBr_3_)_0.15_/Spiro‐OMeTAD/Au	1.15	23.62	0.757	20.55	[75]
	FTO/BCP/[CsPbI_3_]_0.05_[(FAPbI_3_)_0.85_(MAPbBr_3_)_0.15_/Spiro‐OMeTAD/Ag	1.10	22.35	0.776	19.07	[102]
	ITO/MAPbI_3_/Spiro‐OMeTAD/Au	1.15	22.4	0.816	21.08	[26]
	FTO/BIPH‐II/MAPbI_3_/Spiro‐OMeTAD/Au	1.082	22.40	0.714	17.31	[103]
	FTO/PEIE/MAPbI_3‐_ *_x_* Cl*_x_*:Al_2_O_3_/spiro/Ag	1.10	21.1	64.7	15.0	[100]
	FTO/a‐NbOH/MHP/Spiro‐OMeTAD/Au (21.1%)	1.16	23	79	21.1%	[76]
P–i–n ETL‐free PSCs	Au(Cu)/Ti/MAPbI_3_/PTAA/ITO	1.03	22.5	0.78	18.1	[81]
	Au/MAPbI_3_/PEDOT:PSS/ITO	‐0.6∼0.5	20.3	0.40	–	[104]
	Au/MAPbI_3_/PEDOT:PSS/ITO	+(−)0.65 V	+(−)18.5	–	–	[53]
	Au/Ti/MAPbI_3_/PEDOT:PSS/ITO	0.89	24.38	59.49	12.91	[80]
P–i–n HTL‐free PSCs	ITO/MAPbI_3_/PCBM/Al	1.06	21.69	61	11.31	[105]
	ITO/MAPbI_3_/PC_61_BM/Al.	0.98/0.98	17.1/16.9	61.0/74.1	10.22/12.3	[106]
	ITO/CsSnI_3_/PC_61_BM/BCP/Al	0.50	9.89	68	3.56	[107]
	ITO/MAPbI_3−_ *_x_* Cl*_x_*/C60/BCP/Ag	0.75/0.83	21.5/21.35	58.5/57.8	9.5/10.26	[25]
	ITO/MAPbI_3_/ETL/Cu	1.00	19.7	56	11	[23]
	ITO/FA_0.75_Cs_0.25_Sn_0.4_Pb_0.6_I_3_/C60/BCP–Ag or SnO_2_–IZO	0.70	30.2	–	15.4	[108]
	ML‐ITO/MAPbI_3−_ *_x_* Cl*_x_*/C_60_/BCP/Ag	1.12/1.11	22.07/21.57	78.89/78.76	19.42/18.86	[25]
	ITO/MAPbI_3_:F4TCNQ/C_60_/Cu	1.10	22.7	81	20.2	[23]
N–i–p HTL‐free PSCs	Au/CsPbIBr_2_/c‐TiO_2_ F/TO	0.96/0.82	8.7/8.7	56/53	4.7/3.7	[109]
	Ag/MAPbI_3_/CsPbI_3_/CsPbBr_0.3_I_2.7_/CsPbBr_0.7_I_2.3_/MoO_3_/c‐TiO_2_/FTO	0.95	19.21	62.1	11.33	[110]
	C/MAPbI_3_/ZnO/FTO	0.77	18.16	0.51	7.26	[111]
	C/CsPbI_2_Br/SnO_2_/ITO	1.145	12.88	62	9.14	[112]
	C/MAPbBr_3_/c‐TiO_2_/FTO	1.46	7.83	78	8.7	[113]
	Multilayered graphene/MAPbI_3_/c‐TiO_2_/FTO	0.94	16.7	73	11.5	[79]
	Single‐layered graphene/MAPbI/c‐TiO_2_/FTO	0.87	14.2	54	6.7	[79]
	C/PMMA/CsPbI_2_Br/SnO_2_/ITO	1.202	12.64	71	10.95	[112]
TL‐free PSCs	ITO/CsSnI_3_/Au/Ti	0.42	4.80	22	0.88	[114]
	FTO/MAPbI_3‐_ *_x_* Cl*_x_*/Au	0.77	7.11	53	3.2	[115]
	FTO/CsPbBr_3_/C	1.05	4.64	48	2.35	[116]
	Al/MAPbI_3‐_ *_x_* Cl*_x_*/MoO_3_/Au	0.38	26.24	24	2.40	[117]
	Au/MAPbI_3_ single crystal/Au	0.82	2.28	–	1.88	[118]
Modified TL‐free PSCs	Au/OMETP/MAPbI_3_/ClTP/Au	0.55	11.93	48	3.11	[119]
	PEDOT (1.6 nm)‐modified ITO/MAPbI_3‐_ *_x_* Cl*_x_* modified with islands‐like C_60_ (1 nm)/BCP/Ag	1.08	22.3	76	18.2	[42]
	FTO/n‐type MAPbI_3_/p‐type MAPbI_3_/Au	0.80	21.81	46	8.08	[120]

**Figure 4:**
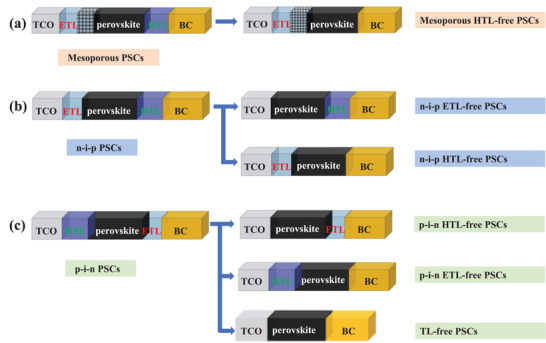
a) Schematics illustration of the mesoporous PSCs and mesoporous HTL‐free PSCs. b) Schematics illustration of the n–i–p PSCs and n–i–p ETL‐free PSCs and n–i–p HTL‐free PSCs. c) Schematics illustration of the p–i–n PSCs and p–i–n ETL‐free PSCs, n–i–p HTL‐free PSCs, and TL‐free PSCs.

Most reported simplified‐structure PSCs are generally referred to as ETL‐free PSCs, HTL‐free PSCs, and TL‐free PSCs. To explore the MHP/electrode contacts, these emerging PSCs can be subdivided into six major classes based on their corresponding full structures. **Figure** **4** shows mesoporous HTL‐free PSCs, n–i–p ETL‐free PSCs, n–i–p HTL‐free PSCs, p–i–n ETL‐free PSCs, p–i–n HTL‐free PSCs, and TL‐free PSCs. The full structures of PSCs with mesoporous TiO_2_ (m‐TiO_2_) (TCO/ETL/m‐TiO_2_/MHP/HTL/BC (back contact)) and the mesoporous HTL‐free PSCs by removing the HTL are also exhibited in Figure /b> 4a. The mesoporous PSCs (TCO/ETL/m‐TiO_2_/MHP/HTL/BC), based on alumina and titania nanoparticle films infiltrated with MHP, were initially the most efficient PSCs. However, a high‐temperature sintering step is typically required to prepare mesoporous PSCs, increasing the complexity of preparation and giving up the utilization of support materials with low melting points. In mesoporous HTL‐free PSCs, the expensive HTL material is abolished, but the troublesome mesoporous structure is retained.^[^ ^72^ ^]^ It can be seen that mesoporous structure is commonly used in HTL‐free PSCs with carbon‐based back electrodes (Table 1). Figure 4b,c presents the planar n–i–p PSCs and planar p–i–n PSCs and their corresponding ETL/HTL‐free devices. Planar n–i–p PSCs and planar p–i–n PSCs, omitting the mesoporous layers, are developed with the sequence of TCO/ETL/MHP/HTL/metal and TCO/HTL/MHP/ETL/metal, respectively. The HTL (ETL) was introduced to harvest charge carriers by the charge carrier selection function at TL/MHP interface.^[^ ^73^ ^]^ The photovoltaic characteristic of these cells is further guaranteed by the ETL and HTL systems in which the doped layers form a built‐in potential for the alignment of the Fermi levels. As expected, the effect of ETL and HTL systems on device performances is more sensitive than that of the traditional PSCs with TCO and back electrodes. However, the charge‐transporting layer is not an indispensable part of PSCs. As shown in Table 1, several planar ETL/HTL‐free PSCs have achieved PCEs exceeding 20%, such as ETL‐free PSCs of tetramethylammonium hydroxide (TMAH)‐modified FTO/MHP/Spiro‐OMeTAD/Au (20.1%),^[^ ^74^ ^]^ nanometer‐thick 4,4′‐(((methyl(4‐sulphonatobutyl)ammonio)bis(propane‐3,1‐diyl))bis(dimethylammoniumdiyl))bis‐(butane‐1‐sulfonate) (MSAPBS)‐modified ITO/(FAPbI_3_)_0.85_(MAPbBr_3_)_0.15_/Spiro‐OMeTAD/Au (20.55%),^[^ ^75^ ^]^ methylammonium acetate modified ITO/MAPbI_3_/Spiro‐OMeTAD/Au (21.08%),^[^ ^26^ ^]^ amorphous niobium oxyhydroxide (a‐NbOH) modified FTO/a‐NbOH/MHP/Spiro‐OMeTAD/Au (21.1%),^[^ ^76^ ^]^ and HTL‐free PSCs of ITO/2,3,5,6‐tetrafluoro‐7,7,8,8‐tetracyanoquinodimethane (F4TCNQ)‐doped MHP/C_60_/bathocuproine (BCP)/Cu (20.2%).^[^ ^23^ ^]^ Even for the TL‐free PSCs, a PCE of 18.2% has been achieved in the device of PEDOT (1.6 nm)‐modified ITO/MAPbI_3_/C_60_‐modified Ag.^[^ ^42^ ^]^ These experimental results suggest that the planar ETL/HTL‐free devices can achieve high performance after interface treatments.

Despite the rapid progress, ETL/HTL‐free PSCs still lag behind the traditional full PSCs in the critical factors of efficiency, stability, and large‐area fabrication to push their large‐scale production and application. The working mechanism of these devices is still unclear. The origin of *V*_OC_, the channel of the PCE loss, the underlying device physics, and the unique aging mechanism of these devices require a comprehensive understanding of MHP/electrode contacts. Understanding the device physics of MHP/electrode contacts is essential for boosting the development of ETL/HTL‐free PSCs. The in‐depth study of MHP/electrode contacts can promote the development of ETL/HTL‐free PSCs, promoting reforms and innovations in solid‐state physics and semiconductor science.

#### 3.1.2 MHP/Electrode Contacts of the ETL/HTL‐Free PSCs

In ETL/HTL‐free PSCs previously reported, the MHP layer is conjugated with TCO, metal, or carbon electrodes. In mesoporous HTL‐free PSCs, the absence of HTL leads to the MHP/metal contact, such as MAPbI_3_/carbon electrodes in the device FTO/c‐TiO_2_/m‐TiO_2_/MAPbI_3_/C and MAPbI_3_/Au contact in the device FTO/m‐TiO_2_/MAPbI_3_/Au. In 2012, Etgar et al. first reported the mesoporous HTM‐free PSC using an Au electrode, with a PCE of about 5.5%.^[^ ^77^ ^]^ By using the carbon materials instead of Au electrodes, some mesoporous HTM‐free PSCs have been reported. Li et al. prepared a MAPbI_3_‐based device with a mesoscopic TiO_2_/Al_2_O_3_ framework structure and single‐wall carbon nanotube‐doped graphite/carbon black counter electrode, achieving a PCE of 14.7% under AM 1.5G illumination.^[^ ^78^ ^]^ Yan et al. employed conductive graphene as the hole extraction electrode in the mesoporous HTL‐free PSCs, leading to the ohmic contact (single‐layered graphene/MHP) and Schottky junction (multilayered graphene/MHP).^[^ ^79^ ^]^ Using carbon electrodes instead of expensive noble metals can significantly reduce the fabrication cost for HTL‐free PSCs. Besides, carbon electrode possesses chemical inertness, water, and heat resistance, which benefit the device's stability. Consequently, the low cost of carbon electrodes can offset the relatively low efficiency of the mesoporous HTM‐free PSCs, reflecting the definite potential for future commercialization.

In the case of planner n–i–p HTL‐free PSCs and p–i–n ETL‐free PSCs, metal electrodes, such as Ti, Au, Ag, Pt, Cu, Al, and carbon‐based electrodes are in contact with MHPs, forming MHP/Metal or MHP/C contacts. In general, p–i–n ETL‐free PSCs with MHP/Metal contact have low PCEs, such as devices with structures of ITO/PEDOT:PSS/MAPbI_3_/Au and ITO/PEDOT:PSS/MAPbI_3‐_ *_x_* Cl*_x_*/Ag.^[^ ^42^, ^53^ ^]^ By using an ultrathin layer of Ti as a cathode interlayer between the metal electrode and MHP film, Shi et al. reported an efficient p–i–n ETL‐free PSC with a device structure of ITO/PEDOT:PSS/MHP/Ti/Au, showing a PCE of nearly 13%, high reproducibility, and hysteresis‐free *J*–*V* curves.^[^ ^80^ ^]^ Later on, Chen et al. increased the PCE of the p–i–n ETL‐free PSCs to 18% with the device of ITO/PEDOT: PSS/MHP/Ti/Au (Cu).^[^ ^81^ ^]^ The improvement of the PCE and stability of the Ti interlayer was attributed to the low diffusivity of Ti atoms and the passivation of surface defects by a bonding layer of Ti with nitrogen (N) atoms in methylammonium anions at MHP/Ti contact. Although the device of ITO/PEDOT:PSS/MHP/Au showed weak photovoltaic effects due to the almost same work functions of PEDOT:PSS and Au electrodes, the device can be as a switchable diode in the dark, leading to a giant switchable photovoltaic effect under illumination, i.e., the photocurrent direction can be switched repeatedly by applying a small poling electric field.^[^ ^53^ ^]^ The origin of the switchable photovoltaic effect in the device of ITO/PEDOT:PSS/MHP/Au was that the poling electric field promoted ion motion and accumulation at MHP/metal interfaces, inducing a doping effect and metastable band bending.

MHP/electrode contacts have gained a substantial attention, and the preparation process of MHP thin films is becoming increasingly mature. By contrast, understanding and controlling of the MHP/electrode contacts become a pivotal issue in improving the performance of the ETL/HTL‐free PSCs. The contact problems can be summarized as the following aspects. 1) The substrate‐dependent film formation of MHP thin films is one factor. As known, film morphology is one of the most critical factors in the preparation of MHP films on electrodes. The grain aspect ratio of the film morphology determines the ratio of film coverage on the substrate and the formation of possible leakage path that causes device shunting. 2) Interface‐dependent driving force for charge transfer and origin of photovoltaic effect is the second factor. The MHP/electrode usually forms Schottky contact, whose depletion region width and built‐in potential are still controversial. Some studies have shown that the change of metal or TCO work function has a noticeable effect on the *V*_OC_ of the device without the transporting layer, indicating that the built‐in potential is closely related to the electrode characteristics and MHP/electrode contact.^[^ ^82^, ^83^ ^]^ 3) Charge carrier transfer and recombination processes at the MHP/electrode and their influence on related MHP‐based device performance are the third factors. The charge carrier dynamics at MHP/electrode contacts play a decisive role in the performance of the MHP‐based devices. A basic understanding of the charge carrier dynamics at the contact focuses on the device physics in ETL/HTL‐free PSCs. Besides, the reaction between metal electrodes and MHP can influence the aging of PSC devices. The interface engineering and electrode modification can adjust the work function. This can realize the matching of energy levels, promote the collection of carriers at the interface, and reduce recombination.

#### 3.1.3 Working Principles of the ETL/HTL‐Free PSCs

**Figure 5:**
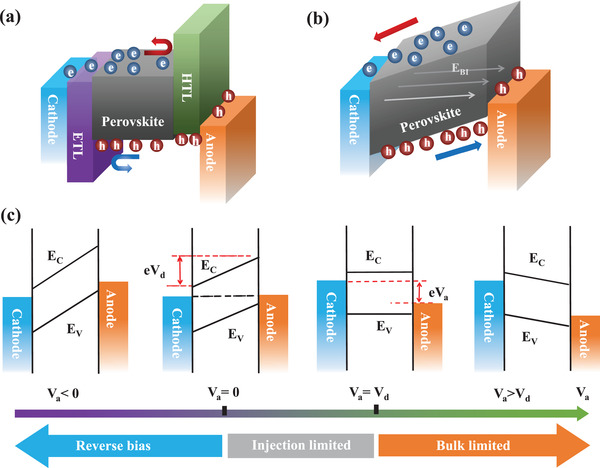
a) Schematics illustration of the working mechanism of the PSCs driven by the charge carrier selection. b) Schematics illustration of the working mechanism of PSCs driven by the built‐in electric filed. c) Schematics illustration of the metal–insulator–metal picture, which shows the energy band diagrams of *V*_a_ < 0, *V*_a_ = 0, *V*_a_ = *V*_d_, and *V*_a_ < *V*_d_.

In recent years, various theories have been developed to explain the operational mechanism of PSCs. These can be fundamentally categorized into two types: the charge selective heterojunction theory and the built‐in electric field theory. As for the charge selective heterojunction, the hole‐selective or electron‐selective contacts are required to create a photovoltage by harvesting electrons at the cathode and holes at the anode, as shown in **Figure** **5**a. The selectivity of charge leads to charge separation and photovoltage behavior. Most of the full‐structure PSCs introduce ETL and HTL as electron‐selective and hole‐selective layer, respectively. Figure 5b illustrates the operation mechanism of the built‐in electric field that is inherent in the device. A built‐in electric field in the MHP film is introduced due to the difference in work functions in the electrodes or the doped charge‐transporting layers under thermal equilibrium conditions. Under the built‐in electric field, the photogenerated electrons move to the cathode, and the holes move to the anode, producing the electromotive force pointing to the cathode. Moreover, the MHP could be either n‐doped or p‐doped, forming a p–n junction at the contacts.

Ideally, the MHP‐based devices could be described via the metal–insulator–metal picture, which has been widely used to explain the working mechanism of intrinsic semiconductor thin‐film devices.^[^ ^84^, ^85^, ^86^ ^]^ Metal–insulator–metal picture is one theory of the built‐in electric field. As shown in Figure 5c, a simple band structure with the bandgap (*E*_g_), the conductive band (*E*_C_), and the valence band (*E*_V_) is shown in MHP semiconductors. The cathode and anode are two Fermi levels or work functions. The electrodes contact MHP semiconductors, forming a cathode–insulator–anode structure. Due to the alignment of the different work functions between the cathode and anode, a uniform band bending produces. As a result, a built‐in electric field is present across the MHP semiconductor, which drives the carriers to transmit toward the corresponding electrode and generates the photovoltage. The model provides an arena for conducting, recombining, and collecting photogenerated electrons and holes, which fundamentally determine the device's performance.

The metal–insulator–metal picture gives a simple and self‐consistent explanation for the operation of MHP‐based PSCs in Figure 5c. Upon a reverse bias voltage, the collection of charge carriers is enhanced due to the enlarged internal electric field. The photogenerated current mainly contributes to the total current because the injected dark current is relatively small. In the short‐circuit condition, the built‐in electric field is only present in the semiconductor layer. Under illumination, the generated charge carriers drift to the contacts driven by the built‐in electric field, resulting in the short‐circuit current of the cell. Under the open‐circuit condition, a voltage is established to offset the built‐in electric field, and no current flows through the device. In consequence, the charge carriers mainly relax to the ground state by recombination. Charge carriers are effectively injected from the electrode into the semiconductor when the applied voltage is larger than *V*_OC_, thus forming non‐negligible dark currents. The recombination rate of the charge carriers and the dark current density significantly influence the shape of the *J*–*V* curves and *V*_OC_.

The metal–insulator–metal picture predicts that the maximum achievable *V*_OC_ of the solar cells is limited by the difference in the work functions between the two electrodes. However, due to the multilayer structure, many factors affect the *V*_OC_ in the thin film of PSCs. *V*_OC_ of full‐structure PSCs is determined by the conduction band of ETL and the valence band of HTL, and is not related to the bandgap of the MAPbBr_3_, which are aligned with the Fermi energy of the electrode.^[^ ^87^ ^]^ As for the ETL/HTL‐free PSCs, *V*_OC_ has a close relationship with the work functions of metal electrodes. However, there are departures in the experimental data from the expectations because ideal MHP/metal contacts can't realize due to the thin oxide layer, dipoles, and chemical reactions on the surface of contact, as well as doping and self‐doping of the semiconductor, which will be further discussed in Sections 4 and 5.

The charge selective heterojunction theory and the metal–insulator–metal picture present the ideal working mechanism of perovskite solar cells. They are macroscopic and rough, without explicit theoretical content. Therefore, it is necessary to carry out parametric modeling for the specific problems of the devices during the analysis or quantitative simulation of the device's performance. In most simulations, drift‐diffusion equations with mobility for charge carrier transport and the Poisson equation for the evolution of electric potential synergistically simulate the photoelectric characteristics of PSCs. Moreover, models for carrier generation and recombination in the bulk of the layers have been appropriately established during the simulation of PSCs. The working principles of the ETL/HTL‐free PSCs on the detailed device physics level account for much of the development of high‐performance devices. The specific models of charge carriers and the charge selective heterojunction theory or metal–insulator–metal picture constitute a unified framework for our understanding of PSCs. However, the modeling of PSCs interface in device physics is far less mature than that of MHP material. As a result, we have not been able to accurately simulate things at the interface of the ETL/HTL‐free PSCs, such as interface recombination, interface dipole layer, tunneling effect, etc. Most of the discussion and research on contact are still isolated experimental work. There are also a few device physics simulation efforts based on experiment achievements to handle the effects of the contacts on the performance of the PSCs. Later in this paper, after summarizing the primary device physics simulation conclusions on the PSCs, we specifically discussed the treatment of the MHP/metal contact by the device physics models during the simulation of ETL/HTL‐free PSCs in Section 6.1. We hope our work could draw attention to modeling the MHP/metal contacts of the perovskite devices.

### 3.2 MHP‐Based Memristors

Memristors, namely, “memory‐resistors,” was first postulated by Chua in 1971 as the fourth fundamental circuit element, after the resistor, the capacitor, and the inductor that has been widely used.^[^ ^149^ ^]^ In 2008, Strukov et al. realized the first memristor by connecting the memristor concept with their TiO*_x_*‐based devices.^[^ ^150^ ^]^ In 2011, Chua showed that two‐terminal nonvolatile memory devices with resistance switching behavior were memristors, regardless of the device materials and physical operating mechanisms.^[^ ^151^ ^]^ In 2015, Yoo et al. reported the first MHP‐based nonvolatile memory devices with a remarkable resistive switching effect.^[^ ^131^ ^]^ In 2016, Xiao et al. demonstrated a two‐terminal MAPbI_3_ device as the ideal candidate for memristors and applied the devices to mimic the biological synapses.^[^ ^21^ ^]^ Since then, many efforts have been devoted to optimizing the component function and understanding the working mechanism.^[^ ^152^, ^153^ ^]^

Almost all of the reported MHP‐based memristors have metal–insulator–metal stacking architectures with an MHP layer sandwiched between two electrodes. Generally, the MHP layer is in contact with top metal electrodes, such as Au, Ag, Cu, Ti, Zn, Pt, and Al. The bottom electrode could be FTO/TiO_2_ compact layer, bare TCO, metal, PEDOT:PSS, etc. When the scanning voltage is applied, the memristors are characterized by memristive characteristics and resistive switching behavior. According to their applications, MHP memristors can be divided into resistive switching memories for resistive random access memory (RRAM) devices and artificial synaptic devices for neuromorphic computing.

#### 3.2.1 MHP‐Based Resistive Random Access Memory

**Figure 6:**
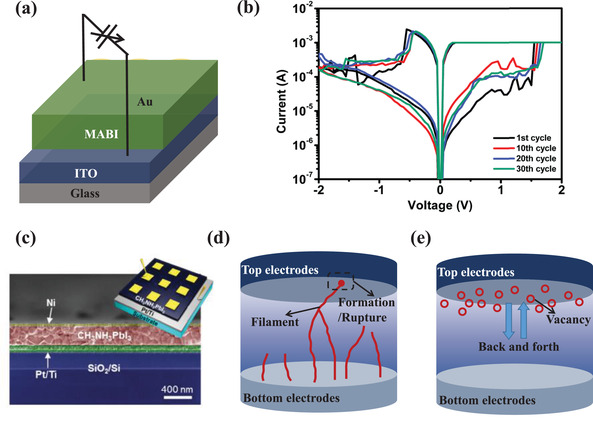
a) Schematic illustration of the memory device with the Au/(CH_3_NH_3_)_3_Bi_2_I_9_(MABI)/ITO structure. b) *I*–*V* characteristics of the Au/MABI/ITO device. Reproduced with permission.^[^ ^88^ ^]^ Copyright 2018, The Royal Society of Chemistry. c) Cross‐sectional SEM image of a fabricated Ni/MAPbI_3_/Pt/Ti/SiO_2_/Si vertical structure and schematic of the metal/MAPbI_3_/metal vertical structure for resistive switching (inset). Reproduced with permission.^[^ ^89^ ^]^ Copyright 2016, Wiley‐VCH. d) The filamentary‐type switching resistive mechanism. e) Interface‐type resistive switching mechanism.

**Figure** **6**a exhibits a typical MHP‐based resistive switching memory with a simple configuration of Au/(CH_3_NH_3_)_3_Bi_2_I_9_(MABI)/ITO.^[^ ^88^ ^]^ From the *I*–*V* characteristics of the Au/MABI/ITO device, a high resistance OFF state and low resistance ON state is present, as shown in Figure 6b. The device showed a resistive switching behavior, the transition between the low and high resistance state under the voltage stimulus. The abrupt transitions between the OFF and ON state happened when the stimuli voltage exceeded a threshold voltage, realizing the SET and RESET processes in the MHP‐based memory devices. The requirements for the resistive switching memories are SET voltages (write voltage), RESET voltages (read voltage), ON/OFF ratio (resistance ratio), endurance, and retention. Under appropriate SET voltage, the memory would turn from the high‐resistance state (OFF) to the low‐resistance state (ON), and the RESET voltage turns the memory from the ON state to the OFF state. The current ratio of two states defines the ON/OFF ratio. Endurance is the maximum number of write cycles, and the preferable value should be better than 10.^[^ ^7^, ^19^, ^152^ ^]^ A data retention time of >10 years should be needed at thermal stress up to 85 °C and minor constant electrical stress.^[^ ^154^ ^]^ **Table** **2** summarizes the configurations and parameters of the MHP‐based memories reported so far. The MHP‐based memory is a potential research field for nonvolatile memory technology of data storage, the internet of things, and artificial intelligence due to its simple structure, low power consumption, high memory density, fast operating speed, and low fabrication cost.

**Table 2:** Summary of device structures and performance parameters of the examples of the MHP‐based memories

Type	Device structure	Set/reset voltage [V]	ON/OFF ratio	Endurance (times)	Retention [s]	Refs.
Ag	Ag/MAPbI_3‐_ *_x_* Cl*_x_*/FTO	1.5/1.5	100	10^3^	4 × 10^4^	[121]
	Ag/MAPbI_3_/Pt	0.13/0.13	10^6^	350	10^4^	[89]
	Ag/MAPbI_3_/Pt	0.18/0.1	10^6^	1338	29 800	[89]
	Ag/MAPbI_3_/Au/Ti/SiO_2_/Si	0.2/0.13	10^7^	10^3^	700	[122]
	Ag/PMMA/MAPbBr_3_ QD/PMMA/ITO/PET	1/1	>10^3^	–	4000	[123]
	Ag/FAPbI_3_/Pt	0.22/0.22	10^5^	1200	10^3^	[124]
	Ag/CsPbBr_3_/PEDOT:PSS/ITO	–/–	<10	300	–	[125]
	Ag/PMMA/CsPbI_3_/Pt	0.18/0.1	10^6^	300	–	[126]
	Ag/CsPb_1‐_ *_x_* Bi*_x_* I_3_/ITO	–/–	≈10	500	10^4^	[127]
	Ag/PMMA/CsPbBr3 QDs/PMMA/ITO	2.6/2.8	10^5^	5000	10^5^	[128]
	Ag/PMMA/CsSnI_3_/Pt	0.13/0.08	7×10^3^	600	7 × 10^3^	[129]
	Ag/DBA_2_PbI_4_/Pt/Ti/SiO_2_/Si	0.7/0.8	10^7^	250	10^3^	[130]
Au	Au/MAPbI_3‐_ *_x_* Cl*_x_*/FTO	0.8/0.6	≈3	100	10^4^	[131]
	Au/MAPbI_3‐_ *_x_* Cl*_x_*/FTO	0.1/0.45	10^4^	400	10^4^	[132]
	Au/MAPbI_3_/ITO/PET	0.7/0.5	10	400	10^4^	[133]
	Au/MAPbI_3_/Pt	1/1	10^4^	500	10^5^	[134]
	Au/MAPbI_3_/FTO	1/1	60	100	10^4^	[135]
	Au/MAPbBr_3_/ITO	–/–	10^3^	10^3^	10^4^	[134]
	Au/MABI/ITO	1.6/0.6	10	300	10^4^	[88]
	Au/Cs_3_Bi_2_I_9_/ITO/PET	0.3/0.5	100	1000	–	[136]
	Au/CsPbBr_3_/FTO	1.5/1.2	10^3^	100	3 × 10^3^	[137]
	Au/Cs_4_PbBr_6_/PEDOT:PSS/Pt	0.6/0.2	100	100	10^4^	[138]
Al	Al/MAPbI_3‐_ *_x_* Cl*_x_*/cTiO_2_/FTO	1.1/1.65	10^9^	–	–	[139]
	Al/MAPbI_3_/p^+^‐Si	3.15/2.21	10^3^	200	10^4^	[140]
	Al/MAPbBr_3_/PEDOT:PSS/ITO	0.2/31	10^6^	120	10^4^	[3]
	Al/MA_2_PbI_2_(SCN)_2_/ITO	–/–	10^7^	10^8^	10^4^	[141]
	Al/MAPbI_3_:PVK (1:1)/ITO	1.57/–	10^3^	–	10^4^	[142]
	Al/PEO:10%MAPbBr_3_/ITO	3.5/–	10^4^	–	10^4^	[143]
	Al/CsPbBr_3_/PEDOT:PSS/ITO/PET	0.6/1.7	10^2^	50	–	[144]
	Al/PMMA:CsPbCl_3_Q Ds/ITO	0.3/2.6	2 × 10^4^	100	10^4^	[145]
Ti	Ti/TiO_2_/MAPbI_3‐_ *_x_* Cl*_x_*/Au	1/1.58	20	–	10^4^	[146]
Ni	Ni/ZnO/CsPbBr_3_/FTO	0.95/0.71	10^5^	100	10^4^	[147]
Pt	Pt/CsPbBr_3_/Cu_2_O/FTO	1.6/1.5	>10^3^	1000	–	[148]

The MHP/electrode contacts play a crucial role in determining the properties of the resistive switching memory devices. First, direct metal film deposition on the MHP layer usually results in electrode‐sensitive device characteristics, including switching endurance, the ON/OFF ratio, and operating voltage. Yan et al. studied the influence of different types of metal electrodes on device performances of resistive switching memory. The devices with the Au/MAPbI_3_ contact achieved a switching endurance of 400 cycles, a constant ON/OFF ratio of 10^4^, and an operating voltage of ≈0.7 V, while devices with Ag/MAPbI_3‐_ *_x_* Cl*_x_* contact achieved a higher ON/OFF ratio of 10^6^ and a lower operation voltage of 0.1 V. Considering that the crystal sizes and morphology of thin MHP films have slight influence on the performance, the importance of MHP/electrode contact was emphasized.^[^ ^146^ ^]^ Chio et al. compared the devices fabricated with Ni and Au top electrodes to the devices fabricated with Ag top electrodes (Figure 6c). They found that devices fabricated with electrochemically stable metal electrodes, including Au and Pt, exhibited operation voltages of less than ±0.14 V, whereas the latter utilized electrochemically active metal electrodes, such as Ag and Cu, exhibited higher operation voltages of over ±0.4 V.^[^ ^89^ ^]^ Second, the influence of MHP/electrode contacts on the MHP‐based devices also reflects the importance of MHP selection. It has been demonstrated that a device with a contact of metal/quasi‐2D MHP layer, prepared by inserting organic cations into a 3D MHP structure, shows an improvement of the ON/OFF ratio from 10^2^ to 10^7^ due to the high Schottky barrier lying at the interface between the active layer and electrodes. The enlarged Schottky barrier was attributed to an enlarged bandgap by the transformation in dimensionality from 3D structures to 2D structures, which hindered the injected carrier density from electrodes to MHP layers and diminished the current movement in high resistance states. Third, to describe the memristive behaviors and resistive switching behavior of the MHP‐based memristors, the function of MHP/electrode contacts was indispensable in forming a self‐consistent explanation. Kim et al. have classified the switching mechanisms of the MHP‐based memristors into interface‐type and filamentary‐type, hinging on the types of conducting path.^[^ ^19^ ^]^ As shown in Figure 6d, filamentary‐type memory devices operate by dynamic formation and destruction of conducting filaments in the insulating layer, which is controlled by the applied voltages. It has been acknowledged that the conductive filaments are produced by the migration of metal ions of the electrode, halide ions, and vacancies of the MHP layer.^[^ ^131^, ^146^ ^]^ Sun et al. investigated the competition between metal and vacancy defect conducting filaments in an Ag/CH_3_NH_3_PbI_3_/Pt device and showed that the formation/diffusion of iodine vacancy (*V*_I_) conducting filaments dominated the resistive switching behaviors when the medium layer thickness was in the range of hundreds of nanometers. The Ag conducting filaments emerged and coexisted with *V*_I_ conducting filaments because the medium layer thickness was reduced to ≈90 nm.^[^ ^155^ ^]^ In general, it is believed that filamentary‐type memory devices usually employ electrochemically active metal electrodes, such as Ag and Al. Figure 6e shows an interface‐type switching type, the resistance change formed by field‐induced effects at the MHP/electrode interface. Yoo et al. suggested a mechanism of trap‐controlled space‐charge‐limited conduction in a memory device that was fabricated with MAPbCl*_x_* I_3‐_ *_x_* sandwiched between Au and FTO electrodes.^[^ ^131^ ^]^ Xiao et al. attributed the resistive switching behavior to the dynamic regulation of the Schottky barrier at MHP/electrode interfaces via ion migration under the poling voltages, which would influence the hole injection barrier lying at the anode and/or electron injection barrier lying at the cathode. The current density tended to be larger at a positive bias through constant favorable polling compared to the case with no ion migration.^[^ ^21^ ^]^

These previous studies have shown that the MHP resistive switching memory devices possess great diversity in device performance and working mechanisms. So far, a stable MHP‐based resistive switching memory device has rarely been reported with high performance. One reason is the lack of effective control of the MHP/metal interface. As a consequence, more efforts need to be made to deal with the complexity of the filament dynamics, the electrochemical process of electrodes, the formation of defects in MHP materials, and ion migration under the electric field.

#### 3.2.2 MHP‐Based Artificial Synaptic Devices

**Figure 7:**
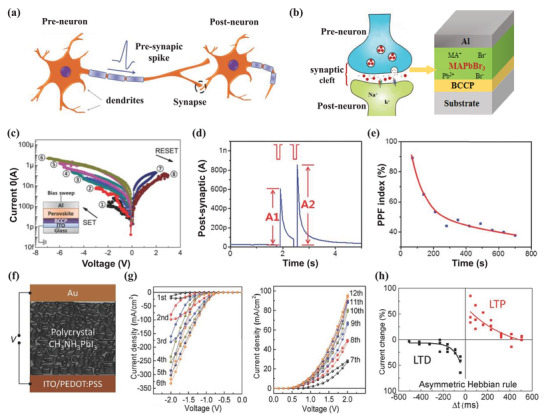
a) Schematic illustration of correlated neurons and their connection by synapse. Reproduced with permission.^[^ ^156^ ^]^ Copyright 2016, Wiley‐VCH. b) Fine structure of a synapse, including presynaptic membrane, postsynaptic membrane, and the synaptic cleft, geometry of an MHP‐based artificial synapse. c) *I*–*V* sweeps with consecutively increased ranges that induced gradual setting and resetting processes. Inset: schematic illustration of the architecture and measurement of the MHP‐based artificial synapse. d) Synaptic enhancement achieved by two successively applied pulses, emulating a biological process of paired‐pulse facilitation (PPF). e) PPF index versus time interval between successive pulses. Reproduced with permission.^[^ ^156^ ^]^ Copyright 2016, Wiley‐VCH. f) Schematics of the device structure. Under external applied bias, the charged ions/vacancies will migrate toward the electrode and result in doping of the MHP near the electrode. g) Memristive characteristics of the device under the positive and negative biases scanning, the scanning rate is 0.1 V s^−1^. h) Asymmetric Hebbian rule. Reproduced with permission.^[^ ^21^ ^]^ Copyright 2016, Wiley‐VCH.

Artificial synaptic devices are the element of artificial neuromorphic systems, which are designed to mimic the whole functions of biological synapses. In the nervous system, the synapse functions as a bridge that transports chemical or electrical signals between a presynaptic neuron and a postsynaptic neuron, as shown in **Figure** **7**a. The synapse plays a role in memory production and brain learning due to the regulation of its plasticity, the strength of the two connected neural pathways, and synaptic weight.^[^ ^157^, ^158^ ^]^ The depression or potentiation of the synaptic weight is regulated by the activity spikes. One of the memory/learning laws to emulate synaptic functions is the spike‐timing‐dependent plasticity (STDP), commonly referred to as the Hebbian learning rule.^[^ ^158^, ^159^ ^]^ For STDP, the relative timing between the prespikes and postspikes determines the sign and magnitude of the synaptic weight change. Spike‐rate‐dependent plasticity (SRDP) is another crucial synaptic learning mechanism in brain behaviors. For the SRDP, synaptic weights are modified by the firing frequency of a train of spikes. In general, a train of presynaptic spikes with high frequency would lead to the potentiation of synapses, while presynaptic spikes with low frequency lead to depression of the synapses, where the threshold frequency exists. The synapse is potentiated when the presynaptic spike has a higher frequency than the threshold frequency. As the presynaptic spikes have a lower frequency than the threshold frequency, the synapse is depressed. Synaptic plasticity could be classified into short‐term and long‐term plasticity (STP and LTP) depending on memory retention.^[^ ^160^ ^]^ The rules of temporal correlations among different spike signals provide methods to design electronic synaptic devices.^[^ ^158^ ^]^

Compared with the program‐based complementary metal oxide semiconductor systems in neuromorphic computing, an artificial synaptic system can theoretically reduce the device counts and system scales due to its real‐time processing of original data obtained directly from the sensitization components. However, it is still challenging to realize the functions discovered in biological synapses with a single artificial synapse. The establishment of efficient neuromorphic computing systems is still limited because of the lack of practical electronic synaptic devices. MHP semiconductors provide new feasibility due to their peculiar photoelectric characteristics. Significantly, the gradual modulation of the resistance in MHP‐based memristors is similar to synaptic plasticity, which constitutes the basis of the brain functions, such as memory and learning.

**Table** **3** summarizes the configurations and performance parameters of the reported MHP‐based artificial synapses. Most MHP‐based artificial synapses have the metal–MHP–metal stacking architecture with an MHP layer sandwiched between two electrodes, which can be considered as one kind of memristors. As shown in Figure 7b, Xu et al. reported an artificial synapse in a two‐terminal configuration of ITO/buffer‐capped conducting polymer electrode/MHP/top electrode.^[^ ^156^ ^]^ The top metal electrode was used to mimic the presynaptic membrane. The ITO substrate was used to emulate the dendrite of a post neuron. Under the operation, the presynaptic spike was applied to the top metal electrode and modulated by the ion‐rich MHP layer. The ITO substrates received the modulated transient signals through the ion‐rich MHP layer. Figure 7c shows the current–voltage (*I*–*V*) switching behavior in the artificial synapse, achieving a gradual current increase through consecutively improving the step voltages from −1 to −6 V. The gradual changes are necessary for artificial synapse applications as they can mimic the consecutively variable synaptic strengths. Figure 7d shows that the device successfully simulates the paired‐pulse facilitation (PPF) functions, one of the most commonly known STP, whose synaptic strength can be improved by a train of pulses. The analogous synaptic weight of the artificial synapse increases when successive stimuli are stressed on the device. The synaptic improvement could correlate with the time interval between presynaptic spikes and postsynaptic spikes, which has also been realized by artificial devices (Figure 7e).^[^ ^156^ ^]^ It is also demonstrated that the device with a configuration of Au/MAPbI_3_/PEDOT:PSS/ITO is one of the ideal candidates to mimic the biological synapse. Figure 7f shows the typical metal–insulator–metal structure with an MHP layer that is sandwiched between the Au electrode and PEDOT:PSS.^[^ ^21^ ^]^ Figure 7g shows the memristive characteristics, presenting a continuously increased dark current when applied consecutive bias sweeps. As shown in Figure 7h, the device has been successfully applied to mimic the Hebbian learning rule of the synapse. The relative timing interval between the prespikes and postspike has influences on both the magnitude and sign of the synaptic weight change.^[^ ^21^ ^]^ These pioneering works suggest that the MHP‐based device is one of the most prospective neural‐like computing methods. Although most reports focused on ion dynamics in interpreting the working mechanism of the artificial synapses, the MHP/electrode contact determines the current density and the changes of the current density under the stimulation of applied electric spikes. The MHP/metal contacts have three leading roles: the transfer of charge carriers, adjustment of the dynamic process for the ions at the interface, and changes of the conductivity with ion distribution. It has been revealed that the Schottky barrier at two sides of the synapse devices is essential to regulate the conductivity with redistribution of ions which act as dopants to change the depletion regions of Schottky contact. Thus, the conductivity/resistance can be gradually regulated through a series of poling spikes, which correspondingly change the distribution of ions.^[^ ^21^ ^]^ Xu et al. proposed that the conductance of buffer‐capped conducting polymer electrode/MHP/Ag device was persistently tuned by electrical pulses, which induced ion redistribution across the thin film or ion injection into conducting polymer layer.^[^ ^156^ ^]^

**Table 3:** Summary of device structures and performance parameters of the examples of the MHP‐based artificial synaptic devices

Device structure	PPF index	LTP	STDP	RTDP	energy consumption/synaptic	Refs.
Al/MAPbBr_3_/BCCP/ITO	91%, Δ*t* = 80 ms	≈90 s	Y		≈20 fJ	[156]
Au/MAPbI_3_/PEDOT:PSS/ITO	Over	30 s	Y		55 fJ/(100 nm)^2^	[21]
Ga(AL)/Bphen/MAPbI_3_/PEDOT:PSS/ITO	192%, Δ*t* = 5.3 ms	>5 s	Y		23 nJ mm^−2^	[172]
Al/MAPbX_3_/Si	2.5% Δ*t* = 240 ms			Y	5.8 pJ	[173]
Graphene/2D (PEA)_2_PbBr_4_ /Au	>1000 s				400 fJ	[174]
ITO/BCCP/OHP/Al	121%, Δ*t* = 20 ms			Y	≈0.7 fJ	[175]
Ag/MAPbI_3_/Ag	40%, Δ*t* = 5 ms					[176]
Si/SiO_2_/CsPbBr_3_ (QDs)/PMMA/pentacene/Au	130%, Δ*t* = 1 ms	≈3000 s			1.4 nJ	[128]
Au/CsPbBr_3_ QDs/Au	132%, Δ*t* = 0.5 ms	2.8 h				[177]

### 3.3 MHP‐Based Field‐Effect Transistors

**Figure 8:**
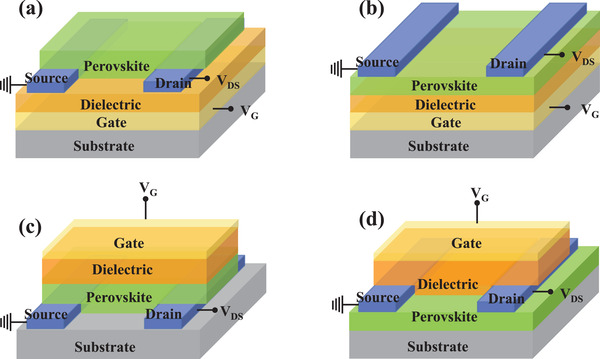
a) Schematic illustration of the bottom‐gate bottom‐contact (BGBC) field‐effect transistor. b) Schematic illustration of the bottom‐gate top‐contact (BGTC) field‐effect transistor. c) Schematic illustration of the top‐gate bottom‐contact (TGBC) field‐effect transistor. d) Schematic illustration of top‐gate top‐contact (TGTC) field‐effect transistor.

The field‐effect transistor that utilizes the applied electric field to control the current flow is one of the main elements in modern electronic circuits. The tuning of current is implemented with a gate terminal, which adjusts the conductivity between drain and source terminals. According to different film deposition orders, field‐effect transistors are divided into four structures, as shown in **Figure** **8**: bottom‐gate top‐contact (BGTC), bottom‐gate bottom‐contact (BGBC), top‐gate top‐contact (TGTC), and top‐gate bottom contact (TGBC). No matter what kind of structure, there definitely exists an interface contact that is sandwiched between the metal electrode and the semiconductor layer.

MHPs are promising materials for high‐performance field‐effect transistors due to the high charge carrier mobility up to 100 cm^2^ V^−1^ s^−1^ solution‐based processability, and mechanical flexibility. However, the development process of MHP‐based field‐effect transistors is cruelly slow, compared with the rapid progress of MHP‐based PSCs, light‐emitting diodes, and detectors. One of the reasons is that the MHP layer has large surface roughness as well as bad contact with the electrode, leading to considerable contact resistance. The transport of carriers in the lateral and interface of the transistor is particularly susceptible to the effects of the crystal plane state of the MHP film and the defects in the crystal grains. Defects in the MHP film give rise to ion migration that brings about a partial screening of the applied gate electric field, resulting in the reduced gate modulation of electronic conductivity, serious current hysteresis, and the reduction of the on–off ratio. Recently, more and more research has focused on MHP/electrode contact, hoping to improve the performance of MHP‐based field‐effect transistors through interface engineering. **Table** **4** summarizes the configuration and performance parameters of the examples of the MHP‐based field‐effect transistors.

**Table 4:** Summary of device structures and performance parameters of the examples of the MHP‐based field‐effect transistors

Semiconductor	Configuration	Modified layer	Dielectric layer	Source and drain	µ_e_/µ_h_ [cm^2^ V^−1^ s^−1^]		On/off ratio [e h^−1^]		Temp. [K]	Refs.
MAPbI_3_	BGTC	MoO_3_	SiO_2_	Au		3.88		10^4^	RT	[162]
MAPbI_3_	TGBC	PEIE	Cytop	Au/Cr	0.5 > 2		10^4^		RT 100	[169]
MAPbI_3_	BGBC		SiO_2_	Au	1.5	4.7	10^5^	8.78 × 10^4^	RT	[168]
MAPbI_3_	BGTC		SiO_2_	Al	7.5 × 10^−3^		3 × 10^3^		150	[165]
Cs*_x_* (MA_0.17_FA_0.83_)_1‐_ *_x_* Pb(Br_0.17_I_0.83_)_3_	TGBC	PEIE	SiO_2_	Y	3.35	4.02	>10^4^	>10^4^	RT	[164]
Rb_0.05_Cs_0.05_FA_0.15_MA_0.75_PbI_3_	TGBC		Cytop	Au/Cr	1.2		10^4^		RT	[16]
PEASnI_4_	TGTC	MH_3‐_SAM	Cytop	Al/C_60_	2.1		2.4 ± 6.4 × 10^4^		RT	[170]
(PEA)_2_SnI_4_	BGBC		SiO_2_	Au/MoO*_x_*		40		10^6^	RT	[167]

#### 3.3.1 Interface Passivation

**Figure 9:**
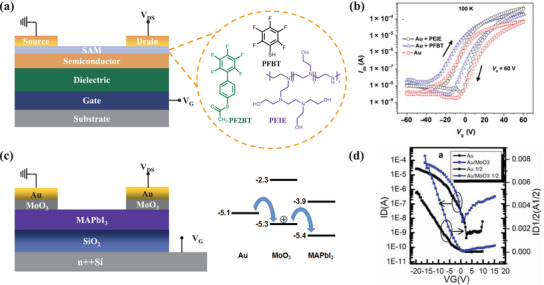
a) The schematic diagram of the structure of the SAMs molecules modified field‐effect transistor and structures of the SAMs molecules PF2BT, PFBT, and PEIE. b) Transfer characteristics at 100 K for different source and drain contact modification for PEIE‐treated Au contacts. Reproduced with permission.^[^ ^161^ ^]^ Copyright 2017, The Authors, some rights reserved; exclusive licensee American Association for the Advancement of Science. Distributed under a Creative Commons Attribution License 4.0 (CC BY). c) The schematic diagram of the structure of the MoO_3_ modified MHP‐based field‐effect transistor and energy level diagrams of Au, MoO_3_, and MAPbI_3_. d) A comparison of typical transfer characteristics of the device with and without MoO_3_ modification at *L* = 100 µm, *W* = 1000 µm, respectively. Reproduced with permission.^[^ ^162^ ^]^ Copyright 2018, IOP Publishing Ltd.

The chemically modified electrode with a self‐assembled monolayer (SAM) is a common method to reduce contact resistance. The schematic diagram of the interface‐modified field‐effect transistor is exhibited in **Figure** **9**a. It has been confirmed that S‐(2′,3′,4′,5′,6′‐pentafluoro‐[1,1′‐biphenyl]‐4‐yl) ethanethioate (PF2BT) and 2,3,4,5,6‐pentafluorothiophenol (PFBT) (Figure 9a) can effectively improve the gold work function using density functional theory simulations. Besides, many other atomic‐level properties, such as molecular structure, tunneling resistance, dipoles, and so on, affect surface energy, work function, and contact resistances.^[^ ^163^ ^]^ When the polymer PFBT modified the interface between MHP and electrode and polyethyleneimine ethoxylated (PEIE) (Figure 9a), both devices exhibited improved field‐effect mobility at 100 K (Figure 9b), although the work function of gold changed in the opposite direction. The field‐effect transistors treated by PEIE have better performance, and the field‐effect mobility for Au contacts improves from 0.02 ± 0.01 cm^2^ V^−1^ s^−1^ to 0.1–0.5 cm^2^ V^−1^ s^−1^ at room temperature. The performance improvement is probably due to the contact‐induced improved nucleation of the perovskite and the crystallization to confined channel regions and is not due to the change of work function.^[^ ^161^ ^]^ Mohammad et al. effectively passivated the MHP layer through surface treatment of PEIE, and diethyl‐(12‐phosphonododecyl) phosphonate was added into the precursor solution to proceed with molecular cross‐linking via hydrogen‐bond interactions between perovskite halogens and dangling bonds presented at grain boundaries. The MHP‐based field‐effect transistors they fabricated have no hysteresis and exhibited good electrical stability under continuous operation. The balanced mobilities of holes and electrons were 4.02 and 3.35 cm^2^ V^−1^ s^−1^, respectively.^[^ ^164^ ^]^ Besides, molybdenum oxide (MoO_3_), a metal oxide with a high work function, has been used as an interface buffer layer in transistors, PSCs, and organic light‐emitting diodes. By inserting a MoO_3_ layer into the surface of the metal electrode and the active layer, the normalized contact resistance obviously decreased from the magnitude of 10^2^ to 10^−1^. At the same time, field‐effect mobility increased from 1.06 to 3.88 cm^2^ V^−1^ s^−1^. The performance improvement is not only because the MoO_3_ film can reduce the injection barrier between the metal electrode and the MHP layer but also can prevent the diffusion of the metal electrode atoms into the MHP film. The schematic diagram of Au/MoO_3_/MAPbI_3_ is exhibited in Figure 9c, and the typical transfer characteristics for devices with and without MoO_3_ modification are shown in Figure 9d.^[^ ^162^ ^]^

#### 3.3.2 Ion Migration and Electrode Degradation

**Figure 10:**
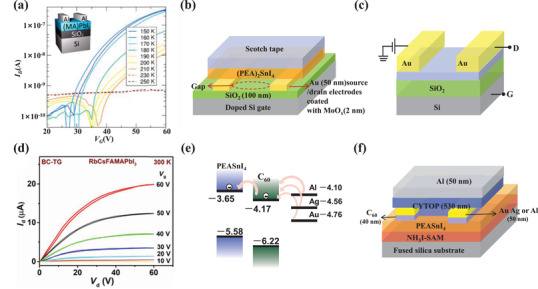
a) Transfer curves of MAPbI_3_‐based field‐effect transistor measured at various temperatures between 150 and 250 K. Reproduced with permission.^[^ ^165^ ^]^ Copyright 2015, American Chemical Society. b) The schematic architecture of field‐effect transistor fabricated by (PEA)_2_SnI_4_ single crystal. c) Schematic of the bottom‐gate, top‐contact (BGTC) device with a micrometer‐thin single crystals hybrid perovskite as semiconductor layer. d) Room temperature output characteristics of bottom‐contact, top‐gate (BCTG) field‐effect transistor fabricated from diethyl ether‐treated RbCsFAMAPbI_3_. Reproduced with permission.^[^ ^16^ ^]^ Copyright 2020, American Association for the Advancement of Science. e) Energy‐level diagram showing electron injection from a metal electrode to PEASnI_4_ via C_60_. f) Schematic representation of the n‐channel PEASnI_4_ transistor with a TCTG structure.

Ionic migration constitutes one of the major difficulties that restrict the development of the MHP‐based field‐effect transistors. Labram et al. found that devices fabricated by MAPbI_3_ exhibit current modulation ability at low temperatures (**Figure** **10**a), but it was not suitable at room temperature.^[^ ^165^ ^]^ Single crystal eliminates the defects of the grain boundary and reduces the obstacles to the lateral and interface transport of carriers.^[^ ^166^ ^]^ By reducing the solubility of (PEA)_2_SnI_4_ solution at low temperature, a bulk single crystal was successfully fabricated. The schematic architecture fabricated by this single crystal is shown in Figure 10b. The carrier mobility of the device is 40 cm^2^ V^−1^ s^−1^ or higher, although the repeatability is poor (less than 1%).^[^ ^167^ ^]^ The MAPbX_3_ (X = Cl, Br, I) single crystal fabricated by spatially confined inverse temperature crystallization strategy has better surface roughness, which approaches to sub‐nanometer level. The device structure is shown in Figure 10c. The room‐temperature field‐effect mobilities in p‐ and n‐channel devices are up to 4.7 and 1.5 cm^2^ V^−1^ s^−1^, respectively.^[^ ^168^ ^]^ In addition to the single crystal, multication doping is also a way to reduce ionic migration. It has been confirmed that the doping of strain‐relieving cations, like Cs^+^, and passivation/crystallization modified cations, like Rb^+^, are valid methods to reduce vacancy concentration and ion migration in the MHP‐based field‐effect transistors. The device doped with multiple cations and treated with Lewis bases exhibits excellent transfer characteristics and ignorable hysteresis at room temperature (Figure 10d).^[^ ^16^ ^]^

The electrode degradation of the MHP‐based field‐effect transistors is also a key issue that limits its development. Au electrodes could be corroded via the reaction with polyiodide produced from the perovskite decomposition.^[^ ^169^ ^]^ If the MHP‐based field‐effect transistors are prepared by the electrode with a lower work function, such as Ag and Al, these devices would not effectively work.^[^ ^168^ ^]^ Besides, the commonly used Pd or Au source/drain electrodes have a high injection barrier of up to 0.8 eV between the MHP and electrodes, producing a high operation voltage (Figure 10e). Matsushima et al. successfully adopted Al source/drain electrodes with low work functions by inserting C_60_ layers between the MHP layer and source/drain electrodes (Figure 10f). The C_60_ film is introduced to suppress the electrode degradation by isolating the electrode and MHP.^[^ ^170^ ^]^ Mohammad et al. replaced the electrode gold with inert yttrium (Y) to avoid chemical interactions between the electrode and perovskite film.^[^ ^164^ ^]^ The thermal evaporation method is a conventional vacuum‐based metal deposition process, which is usually accompanied by high‐energy metal atoms and the collision of metal clusters on the surface of the perovskite, resulting in interfacial metal diffusion. Liu et al. created a more gentle soft‐contact metal electrode fabrication technique. During the fabrication, a thin metal film is laminated onto a 2D semiconductor, which is atomically flat and free of direct chemical bound. The technique creates an interface that is free from chemical disorder and Fermi‐level pinning, which is called van der Waals metal–semiconductor junctions.^[^ ^171^ ^]^ Since the van der Waals metal–semiconductor junctions are soft, the electrode can be peeled off after device operation. Therefore, we can apply the surface analysis technique to the exposed surface in the perovskite crystal so as to explore changes in the interface structure during the operation of the device, which is meaningful for the investigation of interface engineering in the MHP‐based field‐effect transistors.

## 4 The Contacts of MHP to TCO

As conductive and transparent materials, TCOs are widely used as an electrode in optoelectronic devices in which both photons and charges freely enter and exit. For the ETL/HTL‐free PSCs and other MHP‐based devices in that light are involved, TCOs are usually used as electrode substrates, on which the MHP layers are directly prepared. They allow the transmission of light with broadband frequency spectrum due to their large band gaps of above 3.0 eV. In this section, we present the research and understanding of electronic band structures and charge carrier dynamics at the MHP/TCO contacts. We also list the physical and chemical modification methods of the contact as well as their contribution to the performance improvement of these devices. The characteristics of MHP/TCO contacts and their significant roles in related devices are emphasized.

### 4.1 Electronic Band Structures at the MHP/TCO Contact

Deep cognition of the electronic band structure can help to effectively clarify the charge carrier dynamics at the contact and the working mechanism of the ETL/HTL‐free PSCs and other MHP‐based devices involving MHP/TCO contacts. In this section, the studies related to the electronic band structures at the MHP/TCO contact are appropriately reviewed in a theoretical framework of the classical Schottky contact.

#### 4.1.1 Schottky Contact and Band Bending

The MHP/TCO contact is one type of metal/semiconductor contact. Schottky and Mott developed the classical electronic band models and band bending concept to explain the rectifying effect of the metal/semiconductor contacts.^[^ ^178^, ^179^, ^180^ ^]^ The rectifying behaviors of the metal/semiconductor contact arose from an energetic barrier between the metal and the semiconductor, commonly known as the Schottky barrier, due to their difference in work functions. For instance, the alignment of the Fermi energy for the metal with a work function exceeding that of the semiconductor redistributes the charge carriers at the junction, forming a depletion region in the semiconductor and an upward band bending toward the metal. There is also a built‐in electric field in the depletion region due to the polarity of charge distribution.

The Schottky barrier (*ϕ*_Sb_) can be given in a straightforward way as follows

(1) $$\phi_{\mathrm{Sb}}=\phi_{M}-\phi_{S}$$

where *ϕ*_M_ and *ϕ*_S_ are work functions of the metal electrode and the semiconductor, respectively. When *ϕ*_M_ > *ϕ*_S_, the band bending is upward toward the metal electrode, producing a barrier of electrons transferring from the semiconductor into the electrode. On the other hand, the upward band‐bending has no obstacle to the motion of holes from the semiconductor into the metal. If the semiconductor is p‐doped, the metal/semiconductor gives an ohmic contact.

From the view of the metal electrode, there is another energy barrier *ϕ*_b_ that the electron has to surmount when it is thermally injected from the metal into the semiconductor. For n‐type semiconductor, it is given by

(2) $$\phi_{b}=\phi_{M}-\chi_{S}$$

where *χ*_S_ is the electron affinity of the semiconductor, which is defined by the difference between the bottom of the conduction band and the vacuum level.

**Figure 11:**
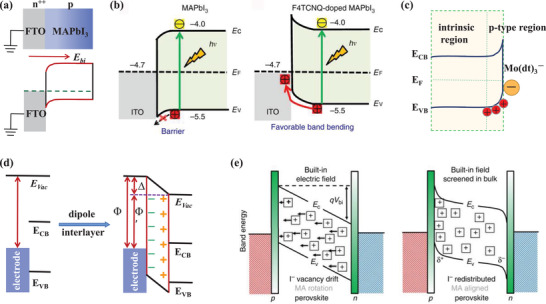
a) Downward energy band bending (toward to the ITO substrate) and built‐in electric field schematics of the FTO/MAPbI_3_ contact. b) A doping‐induced band‐bending shift from downward to upward at the ITO/MAPbI_3_ contact. Reproduced with permission.^[^ ^23^ ^]^ Copyright 2018, Springer Nature. c) Illustration of steep change of electronic band structures at the surface of the MHP crystal due to the charge‐transfer reaction with Mo(tfd‐CO_2_Me)_3_. d) Illustration of the reduced work function of the electrode due to interfacial dipole‐induced *E*_vac_ steep shift Δ. e) Illustration of ion migration and the induced band bending. Reproduced with permission.^[^ ^3^ ^]^ Copyright 2015, Springer Nature.

Based on Schottky's theory, the Fermi level of MHP aligns with that of TCO via charge flow. As a result, band bending is created by the electric field between the MHP semiconductor and the TCO due to the charge transfer. Theoretically, the height of the Schottky barrier and the width of the depletion region are determined by the Fermi level and doping carrier density of the MHP layer, which contacts with the TCO with a definite work function. As shown in **Figure** **11**a, the band bending of the MAPbI_3_ layer at the TCO/MAPbI_3_ contact has a downward direction to the TCO. Similar contacts and band bending have been reported in several studies based on the fact that the Fermi energy of TCO (4.4–4.9 eV) is shallower than that of MAPbI_3_.^[^ ^23^, ^27^, ^181^ ^]^ This is consistent with the single‐side abrupt n^++^–p junction model adopted by Wang et al. to explain energy levels at the FTO/MAPbI_3_ contact. The FTO was considered as an n^++^ electrode in this system, and the depletion region was chiefly distributed in the MHP layer, considered as a p‐type doped semiconductor.^[^ ^181^ ^]^ However, a quite different upward band bending direction toward the TCO was also reported at the TCO/MHP contact in some cases, indicating that the Fermi energy of TCO (4.4–4.9 eV) is deeper than the Fermi level of MAPbI_3_.^[^ ^26^, ^103^, ^182^ ^]^ Thus, there are no unanimously accepted band bending, Schottky barrier, and the width of depletion region detected at the TCO/MHP contact. One possible reason is that MHP materials are easily doped. Many computational and experimental studies have shown that MHP is self‐doped by adjusting the ratio of PbI_2_ in the process of film preparation.^[^ ^183^, ^184^, ^185^ ^]^ This means that the Fermi level of MHP is closely related to the preparation process that has been proved by several groups.^[^ ^186^, ^187^ ^]^ The time‐resolved photoluminescence (TRPL) decays revealed that MHP was changed from p‐type doped (none annealing) to n‐type doped (annealing at 100 and 150 °C).^[^ ^184^ ^]^ It was also found that the ions/vacancies can n‐dope/p‐dope the perovskite. In addition, MHP has been revealed to be light doped and electrochemically doped.^[^ ^5^ ^]^ As a result, it is hard to obtain an intrinsic MHP semiconductor, i.e., an almost pure semiconductor with no impurities and a well‐defined Fermi energy.

In most PSCs, the MHP materials have not been deliberately doped. The depletion region could be much larger than the thickness of the MHP layer, and the band bending extends several hundreds of nanometers in depth. The extended band bending and large depletion region of the MHP/TCO junction suggested a built‐in electric field across the whole MHP layer, which was proved by the Mott–Schottky analysis on the MAPbI_3_/FTO contact. The doping density has been estimated to be 1.35 × 10^14^ cm^−3^ from the Mott–Schottky analysis, which is several orders of magnitude lower than the density of the n‐doped semiconductor. Such a low doping density is not able to produce a band bend in tens of nanometers. The depletion region width has also been evaluated with a value of up to micrometer‐order.^[^ ^181^, ^188^, ^189^ ^]^

Doping is an effective way to regulate the Fermi energy of the semiconductors and to tune the band bending at the contact. MHP/TCO Schottky junctions with a certain depletion region can be realized by intentional doping of the MHP layer. In recent years, doping strategies have been developed to improve the performance of ETL/HTL‐free PSCs by adjusting the band structure at the TCO/MAPbI_3_ contacts. Wu et al. reported a molecular‐dopped MAPbI_3_ layer with a strong electron‐withdrawing molecule, F4TCNQ, which led to a shift from a downward band‐bending (toward the ITO substrate) to an upward band bending at the ITO/MAPbI_3_ interface, as shown in Figure 11b. The ITO/MAPbI_3_:F4TCNQ with an upward band bending was employed in a simplified device structure of ITO/MHP/ETL/Cu with an improved PCE of above 20.0% due to the enhanced efficiency of extraction of photoexcited holes from MHP direct to the conductive substrate.^[^ ^23^ ^]^ Lu et al. manipulated the band bending by doping the MHP layer with copper because the Fermi level does not match with that of ITO/(CH_3_NH_3_)_1‐_ *_x_* Cu*_x_* PbI_3_ heterojunction. It was revealed that the copper doping induced a shift work function of the (CH_3_NH_3_)_1‐_ *_x_* Cu*_x_* PbI_3_ films measured by Kelvin probe force microscopy.^[^ ^190^ ^]^ Wang et al. found MAPbI_3_ could be n‐ or p‐self‐doped by regulating the proportion of PbI_2_ and MAI in the precursor solution. They derived the energy diagram of MHP with all kinds of precursor proportions from their ultraviolet photoelectron spectroscopy measurements and found that when the precursor ratio was increased from 1.0 to 1.7, the Fermi level was pushed up from above the middle bandgap (0.13 eV) and below the conduction band (0.35 eV), indicating a precursor ratio relevant to n‐doping. Conversely, the Fermi level was pushed down close to the maximum of the valance band by decreasing the precursor proportion.^[^ ^185^ ^]^

#### 4.1.2 Steep Change of Electronic Band Structures

In the absence of bulk doping, the interface charge will produce a change in the electronic structure of MHP/TCO contact. The interface charge leads to steep band bending at the contact and effectively tunes charge carrier dynamics. Two common strategies to import interface charges, surface doping and the introduction of interfacial dipoles, are crucial for interfacial engineering methods in MHP‐based devices.

Recently, surface doping strategies have been reported to tune the electrical properties of the MHP films. As exhibited in Figure 11c, Noel et al. confirmed the charge transfer between the MHP and dopant complex, such as a strongly oxidizing molybdenum tris(dithiolene) complex and 4‐tert butylpyridine (tBP), by photoelectron and solid‐state nuclear magnetic resonance spectroscopies. The treated MHP film presented a shift in the work function and a twofold increase in the conductivity of the MHP film, indicating one type of p‐doping in the surface by the strongly oxidizing complex. As a result, an increased hole‐selective homojunction was constituted by the p‐doped interface, which promoted the device performance of PSCs with a PCE of 21%.^[^ ^191^ ^]^ Perry et al. coated cobaltocene onto the MHP surface, which acted as an n‐type dopant through charge transfer from the small molecules to MHP. The conductivity of MAPbI_3_ thin films can be tuned over several orders of magnitude because of the adjustable Fermi level.^[^ ^182^ ^]^ Arramel et al. reported the surface molecular doping of CsPbBr_3_ via using zethrenes. They assembled the heptazethrene triisopropylsilyl ethynylene and its homologue, and octazethrene on CsPbBr_3_ single crystal and measured the interfacial electronic properties via optical and photoelectron spectroscopy. It can be seen that a dipole layer formed at the contact due to the van der Waals force and the dispersion force from bulky triisopropylsilyl‐substituent group. The photoemission spectroscopy revealed that the electrons transfer from heptazethrene triisopropylsilyl ethynylene molecules to the CsPbBr_3_ while the holes transfer from octazethrene to CsPbBr_3_ in the other case. This work indicated that molecules like heptazethrene triisopropylsilyl ethynylene could be used to generate the interfacial dipoles at the perovskite surface.^[^ ^192^ ^]^

In general, it is difficult to incorporate some dopants into the MHP crystal structure. Surface doping could be the most common way of MHP doping. Metal ions, such as silver, strontium, and cerium ions, have low solubility in MHP crystals. These metal ions in the precursor are excluded from the MHP crystal to surfaces, where electronic states near the conduction band minimum of MHP semiconductor could be introduced, resulting in an n‐doping surface with a carrier concentration of up to eight orders of magnitude.^[^ ^193^ ^]^ It has been shown that a little Sr doped into the precursor can induce an effective surface modification by segregating at the MHP surface. Sr changed the energy band at the contact, promoting the n‐typed feature and strong surface band bending. The PSCs with MHP‐surface modification by Sr showed a *V*_OC_ of 1.18 V and further enhanced up to 1.23 V by adding a polymer layer.^[^ ^194^ ^]^

In addition to surface doping, aligned dipoles could also lead to steep electronic structure change at the MHP/TCO contact. Many reports have demonstrated that the aligned dipole layers could be achievable by chemical and physical adsorption of polar molecules with a permanent dipole moment.^[^ ^26^, ^70^, ^95^, ^195^ ^]^ As shown in Figure 11d, the introduction of a dipole layer oriented outward from the surface can shift the vacuum level (*E*_vac_) of the energy band in several angstroms at the contact and reduce the effective work function of the electrode. The dipole layer could be assembled with orientation inward from the surface, yielding an enlargement of the effective work function of the electrodes. Another benefit of the dipole layer is that it aligns the energy levels between the TCO and the MHP, which is one of the main factors affecting the charge transporting at the interface. It is worth emphasizing that MHP/TCO contact‐induced electronic structure constitutes the theoretical basis of interface engineering, which can alter the performance of the MHP/TCO contact‐related devices. The TCO modification by introducing aligned surface dipoles is reviewed in Section 4.4.

#### 4.1.3 Ion‐Induced Band Bending

Ionic transport and accumulation are ubiquitous in MHP‐based devices. On the one hand, the chemical process is closely related to the decomposition of MHP film, device stability, and electrode corrosion. On the other, it could dynamically adjust the current response of the device to voltage through the ion‐induced band bending, such as the well‐known current–voltage hysteresis effect of the MHP‐based PSCs, the screening effect of MHP‐based field‐effect transistors, and resistive switching memory devices. Eames et al. investigated the activation energy of ion migration in MAPbI_3_ by the first‐principles calculations and compared the results with kinetic data extracted from the *J*–*V* response of PSCs. The results indicated that MHPs were mixed ionic–electronic conductors.^[^ ^3^ ^]^

As shown in Figure 11e, an electric field is present in the MHP layer due to the built‐in voltage or applied voltage, and the migration of iodide vacancies and iodide ions produces band bending due to screening effects. The band bending can enhance or counteract the built‐in potential, depending on the direction of the applied voltage, resulting in a weakened or enhanced collection efficiency of photogenerated charge carriers. The mobility of the ions is low, which therefore responds slowly (seconds to minutes) to voltage scanning with a scanning rate from one to a hundred times per second. The lag of the ions’ response to the scanning voltage is believed to be responsible for the observed hysteresis from the current–voltage curve in PSCs and MHP‐based field‐effect transistors. The hysteresis reflects that the current density is mainly dependent on the biasing history, such as scanning rate, scanning step, and voltage scanning range.^[^ ^51^, ^69^, ^196^ ^]^ The ion‐induced band bending has also been adopted by Xiao et al. to explain the giant switchable photovoltaic behaviors in Au/MAPbI_3_/PEDOT:PSS/ITO devices, i.e., the photocurrent direction was completely flipped during voltage scanning. Once the positive voltage was applied, the vacancies would move toward the Au electrode and accumulate, leading to n‐doped MHP near the Au electrode. Similarly, the ions moved to the ITO side, leading to a p‐doped MHP layer near PEDOT:PSS layer. As a result, a p–i–n homojunction structure formed. Then, a reverse bias could flip a p–i–n structure to an n–i–p structure by poling the ions in the opposite direction. The flipping of the device from a p–i–n structure to an n–i–p structure by the time‐dependent drift of ions in an electric field explained the memristive dark‐current and photocurrent of the Au/MAPbI_3_/PEDOT:PSS/ITO device.^[^ ^53^ ^]^ Deng et al. reported a light‐induced self‐doping effect in an MHP‐based device under continuous illumination, which has been demonstrated by the band bending because of redistribution of ions/vacancies driven by a photovoltage‐induced electric field.^[^ ^104^ ^]^ The ion distribution has been revealed to regulate the Schottky barrier at both sides of the MHP‐based synapses. During the regulation, the ions act as dopants to change the band bending and the depletion regions of Schottky contact. The negative bias scanning gradually sweeps the negatively charged ions to one side of the MHP layer and sweeps the positively charged vacancy to the other, resulting in a device with n–i–p polarity. When the device is applied with positive continuous scanning voltage, the ions move in an opposite manner, producing a p–i–n polarity. Thus, the conductivity or resistance of the MHP‐based synapse devices can be accordingly adjusted by applying a train of poling voltage spikes.^[^ ^53^ ^]^

### 4.2 Charge Carrier Dynamics at the MHP/TCO Contact

The charge carrier dynamics in the bulk of the isolated MHP layer have been studied and reviewed.^[^ ^4^, ^5^, ^46^, ^197^, ^198^ ^]^ Free electrons and holes with high diffusion length are produced with high yield due to the efficient light absorption and exciton‐free property of the MHP. The charge carrier dynamics at many MHP/electrode contacts have not been clarified. Clarifying the carrier dynamics at the MHP/TCO contact can generate a standard contact model.

**Figure 12:**
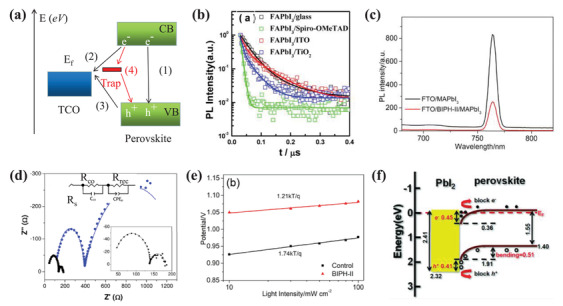
a) Schematic illustration of the charge carrier transfer and charge carrier recombination at the MHP/TCO contact. b) Photoluminescence decay curves of MHP layer on different quenching substrates. Reproduced with permission.^[^ ^204^ ^]^ Copyright 2015, American Chemical Society. c) A decreased steady‐state PL spectra at FTO/MAPbI_3_ when the FTO is modified by hydroxyethyl‐functionalized imidazolium iodide (BIPH‐II) ionic liquid. Reproduced with permission.^[^ ^103^ ^]^ Copyright 2020, Wiley‐VCH. d) Nyquist plots for the MHP solar cells with (black circles) and without (blue circles) a ZnO ETL, as measured under 1 sun illumination and the Equivalent circuit employed to fit the Nyquist plots. Reproduced with permission.^[^ ^205^ ^]^ Copyright 2014, American Chemical Society. e) A reduced ideality factor from 1.7 to 1.2 after the modification of FTO/MAPbI_3_ contact by BIPH‐II, revealed by dependence of *V*_OC_ on light intensity for PSCs with FTO/MAPbI_3_ contact. Reproduced with permission.^[^ ^103^ ^]^ Copyright 2020, Wiley‐VCH. f) Formation of the PbI_2_ and the Energy level diagram of the valence and conduction band at the contact. Reproduced with permission.^[^ ^204^ ^]^ Copyright 2016, Royal Society of Chemistry.

The charge carrier dynamics at the MHP/TCO are shown in **Figure** **12**a. The transfer of photogenerated charge carriers across the contact and their recombination are two typical processes at the contact which have attracted extensive attention in ETL/HTL‐free PSCs.

#### 4.2.1 Charge Carrier Transport to the Contact

Whether the charge carriers diffuse or drift toward the MHP/TCO interface under an internal electric field is debatable.^[^ ^76^, ^199^ ^]^ The carrier diffusion length can reach hundreds of nanometers, or even 1 µm,^[^ ^200^ ^]^ demonstrating that the electron and the hole have high mobility and low bulk recombination (Process (1) in Figure 12a). Thus, most carriers are exposed to the MHP/TCO contact, while the film quality improves, and the carrier recombination in the bulk of MHP thin film reduces. This has been further supported by the devices developed by Han et al., ETL‐free PSCs achieved efficiency above 19% by using MHP films with optimized film quality and microsecond carrier lifetimes. This work revealed that charge carrier dynamics at the MHP/metal contacts were responsible for the performance of ETL/HTL‐free PSCs.^[^ ^27^ ^]^

Introducing the hole transporting layers in PSCs aims to block electron transfer from MHP to TCO (Process (2) in Figure 12a) and to facilitate the transfer of holes. By contrast, the introduction of electron transport layers in PSCs aims to block hole's transfer from MHP to TCO (Process (3) in Figure 12a) and to facilitate the transfer of holes. Early studies showed that the poor performance of ETL/HTL‐layer free PSCs is due to the absence of charge‐carrier blocking layers.^[^ ^73^, ^76^, ^201^ ^]^ Accordingly, the photogenerated electrons and holes can be injected into the TCO, which leads to contact‐related interface recombinations or back‐recombination once the transporting layer is removed. Generally, the back‐recombination is one of the crucial surface recombination via minority carriers at the contact. The surface recombination, an important parameter limiting the performance of solar cells, was quantified by the surface recombination velocity (SRV).^[^ ^202^ ^]^ Apart from the nonradiative recombination due to the mid‐gap states introduced by defects or dangling bonds, the surface recombination rate (*R*_s_) via minority carrier concentration (Δ*n*_s_) at the MHP contact

(3) $$R_{S}=\mathrm{SRV}-\Delta n_{s}$$

SRV is a factor that indicates a boundary condition for the minority carriers during the device modeling calculations and the interpretation of photoluminescence (PL) lifetime measurement, where the SRV was extracted from the plateau value of the PL decay time, several orders of magnitude is generally smaller than that of traditional semiconductors (the order of magnitude 10^5^ cm s^−1^).^[^ ^202^, ^203^ ^]^ Wang et al. showed that the SRV of glass/perovskite or perovskite/transport layer by passivation has low SRV (≤10 cm s^−1^) values compared to other transporting materials such as PEDOT:PSS, Spiro‐OMeTAD, PCBM, and MoO_3_ with high SRV values of ≈10 to ≈5000 cm s^−1^. The SVR can be measured by PL decay assuming that surface recombination is the unique nonradiative loss pathway. However, the SVR needs to be further discussed because PL quenching (decay time) may be induced by other factors, such as charge extraction by the contacts.^[^ ^202^, ^203^ ^]^

Band bending in the MHP layer and the built‐in electric field can block the transfer of one type of carrier and facilitate the opposite carrier. Wu et al. changed the downward band bending at the ITO/MAPbI_3_ interface to an upward band bending by turning the MAPbI_3_ into more p‐typed. The band bending was found to effectively extract photoexcited holes from the MHP layer to the TCO, leading to HTL‐free PSCs with a stabilized PCE of above 20.0%.^[^ ^23^ ^]^ In ETL‐free PSCs, a downward band bending toward TCO should be developed to facilitate the extraction of photoexcited electrons from MHP to the TCO substrate. For this purpose, the TCO substrates were modified by introducing an interfacial layer, for example, tetramethylammonium hydroxide,^[^ ^74^ ^]^ the insulate BCP interlayer,^[^ ^102^ ^]^ and polar nonconjugated small‐molecule electrolyte,^[^ ^70^, ^95^ ^]^ or by in situ assembling layers of methylammonium acetate ionic liquid that also acts as the effective processing solvent in preparing perovskite thin films.^[^ ^26^ ^]^ The band bending in adjusting carrier dynamics indicated that the interface recombination caused by carrier reverse conduction is not a limiting factor in improving the efficiency of ETL/HTL‐free PSCs.

#### 4.2.2 Charge Carrier Extraction by TCO

PL measurement is an effective method to detect the impact of the MHP/TCO contact on the charge carrier dynamics. It is based on the record of photons from the MHP layer emitted by radiative recombination. The radiative recombination process differs from the nonradiative recombination processes because of the different time scales of the carrier processes. When the MHP layer contacts TCO, the nonradiative processes, such as the carrier extraction and interface recombination, will compete with the radiative recombination process, resulting in the PL quenching and an accelerated PL decay. On this basis, time‐resolved PL (TRPL) has been used to reveal the charge transfer dynamics or recombination by the average charge carrier lifetime or the key recombination rate constants.^[^ ^206^, ^207^, ^208^ ^]^ The PL quenching measured by steady‐state PL spectra of the MHP layer on different substrates reflects the relative rate of carrier extraction from the MHP layer by substrates, indicating the charge transfer from the MHP layer to the TCO electrode.

PL measurement shows that the photogenerated charge transfer at the TCO interface is not efficient, which increases the possibility of charge recombination in the bulk of the MHP layer. The TRPL spectra of Cl‐FAPbI_3_ films on glass, Spiro‐OMeTAD, ITO, and TiO_2_ were measured, respectively. As shown in Figure 12b, transient PL decay of Cl‐FAPbI_3_ on ITO almost has the same decay time of 68 ns as that of Cl‐FAPbI_3_ on glass. The PL of Cl‐FAPbI_3_ on TiO_2_ and Spiro‐OMeTAD shows a significantly reduced decay time of 14 and 6 ns, respectively.^[^ ^204^ ^]^ The results indicated that the TCO electrode is not an effective PL quencher, and charge carriers extraction by the TCO substrate is less effective than ETL/HTL. The conclusion was confirmed by the steady‐state PL spectra, which showed ≈40% of PL quenching in the cases of MHP on ZnO or ITO,^[^ ^205^ ^]^ and 50% of MHP on FTO,^[^ ^95^ ^]^ while there was almost PL quenching in the case of MHP upon contact to ETL and HTL.^[^ ^205^ ^]^ The inefficient charge carrier extraction by the TCO electrode is an important factor for the PCE loss in ETL/HTL‐free PSCs.

The charge transfer across the MHP/TCO contact can be enhanced by optimizing the contact between MHP and TCO electrodes. Ultraviolet/ozone treatment is a widely used surface cleaning method. During the surface cleaning process, the ozone reacts with the surface contaminants on the TCO substrate, forming a cleaner TCO surface that has a better affinity to the MHP precursor. It has been proved that the ultraviolet/ozone (UVO) treatment can ensure the full coverage of the MHP on TCO substrates.^[^ ^99^ ^]^ In consequence, an electrical MHP/TCO contact is achieved by an improved physical MHP/TCO contact. The steady‐state PL has shown various degrees of emission quenching when the MHP contacts TCO substrates with and without UVO treatment. It was observed that about 70% of PL quenching happened when the MHP contacted the TCO treated by UVO, while only about 50% of PL quenching was observed when the MHP contact the TCO without UVO treatment.^[^ ^99^ ^]^

Except for the physical treatment of the TCO substrates, tuning the energy band structure at the interfaces is another way to enhance interfacial electron extraction. As shown in Figure 12c, decreased PL intensity is present by introducing a self‐assembly hydroxyethyl‐functionalized imidazolium iodide (BIPH‐II) ionic liquid on the FTO, indicating an enhanced electron extraction from the MHP film to FTO. The reduced work function of the FTO substrate and an interface energy band structure enhanced the electron extraction ability, blocking the holes transporting to the FTO electrode. The work function of FTO was reduced to 4.40 eV, leading to a 0.2 eV reduction compared to the work function of bare FTO. Apart from the decreased work function of FTO, BIPH‐II also showed a deep highest occupied molecular orbital energy level of 8.19 eV, which could effectively block the transfer of holes from MHP film to FTO. Consequently, the ETL‐free PSCs achieved remarkable PCE improvement from 9.01% to 17.31% by self‐assembling BIPH‐II ionic liquid on the FTO substrate.^[^ ^103^ ^]^ More techniques for contact energy band optimization are given in Section 4.4.2.

Although the PL measurement exhibits insightful information on the charge carrier dynamics at the MHP/TCO contact, there is an apparent inconsistency in explaining the PL quenching and the decay times of the TRPL because they cannot distinguish the processes of charge carrier extraction, interface recombination or charge carrier conduction near the contact. Significantly, the effect of trap‐induced charge carrier recombination (Process (4) in Figure 12a) on the PL quenching is not considered. Understanding the average lifetimes extracted from the TRPL is somewhat difficult. The above analysis only ascribes the cause of average lifetime change to the charge carrier extraction by the TCO electrode. As a result, the PL measurement cannot clarify detailed carrier dynamics information for all processes shown in Figure 12a. Nevertheless, it would be helpful to explain the results of the PL measurement by physical models, which include the possibility of recombination at the contact, rate of electron/hole transfer to the TCO, and details of the MHP layers.

#### 4.2.3 Charge Transfer Resistance

Another parameter that directly reflects the electrical contact character of the interface is the charge transfer resistance also called contact resistance or interface resistance. The charge transfer resistance is measured by electrochemical impedance spectroscopy (EIS), an effective method to study charge transfer at the contact and charge carrier recombination.^[^ ^209^, ^210^, ^211^ ^]^ In an EIS measurement, a small alternate current signal with a wide range of frequencies is applied to the device, and differential current outputs are accordingly measured. Then, the impedance of the PSCs is calculated as the Nyquist plot, as shown in Figure 12d. The Nyquist plot usually presents two separate resistance–capacitance (RC) arcs when the device is measured under one sun illumination. As shown in Figure 12d, the left part of the Nyquist plot is the high‐frequency RC arc, and the right part of the Nyquist plot is the low‐frequency RC arc. Most Nyquist plots only show low‐frequency RC arcs if the device is measured in the dark condition.^[^ ^103^, ^181^, ^212^ ^]^

As to the impedance spectrum measurement results, the PSC is like a simple RC circuit with a series resistance, which can well fit the Nyquist curve with suitable resistance and capacitance. In measuring of ETL/HTL‐free PSCs, the high‐frequency resistance element is ascribed to charge transfer resistance (contact resistance) at MHP/TCO contacts, reflecting the interfacial charge transfer processes. In addition, the low‐frequency RC element is ascribed to recombination resistance, indicating a charge recombination process. Only recombination resistance is extracted from the Nyquist plot when measured in the dark. One of the advantages of the EIS method is that the devices are measured in a working state.

Figure 12d shows a typical Nyquist plot for the PSCs with (black circles) and without (blue circles) an ETL (ZnO in this case) measured under one sun illumination. As expected, the device without ZnO ETL has an increased contact resistance (the blue Nyquist plot has a bigger high‐frequency RC arc than the black Nyquist plot), indicating a reduced charge transfer at the contact is inconsistent with the measurement of PL. However, the MHP/ITO contact device presents a more considerable recombination resistance than the device with ZnO ETL, suggesting that surface recombination is reduced by removing the ZnO ETL. In other words, the surface recombination rate at MHP/TCO contact is lower than the rate at MHP/ZnO contact.^[^ ^205^ ^]^ The Nyquist plot measurement also reflects the effect of TCO pretreatment on devices. The charge transfer resistances of devices fabricated with untreated TCOs are significantly larger than those fabricated with TCOs treated by UVO or plasma cleaning.^[^ ^95^ ^]^

In EIS measurement, the charge recombination was revealed by the recombination resistance extracted from the low‐frequency RC arc. In most cases, ETL/HTL‐free PSCs with modified MHP/TCO contact show increased recombination resistance than unmodified MHP/TCO contact PSCs, suggesting that most MHP/TCO contact modifications can effectively reduce the charge carrier recombination at the contact.^[^ ^95^, ^103^, ^181^ ^]^

#### 4.2.4 Charge Carrier Recombination

Charge carrier recombination is one of the main factors influencing PSCs’ performance. It not only reduces the *V*_OC_ but also decreases the fill factor. The charge carrier recombination in competent PSCs has been interpreted as band‐to‐band radiative and nonradiative recombination. The nonradiative recombination loss limits the performance of current PSCs. For ETL/HTL‐free PSCs, the carrier recombination at MHP/TCO interface is an important factor of nonradiative recombination. However, the origin and proportion of nonradiative recombination are still a problem.

Traditionally, the recombination information is usually deduced from the light intensity‐dependent measurement. The type of charge carrier recombination is revealed by the ideality factor obtained by measuring the *V*_OC_ versus the light intensity, according to^[^ ^47^, ^213^ ^]^

(4) $$J_{R}\left(V_{\mathrm{OC}}\right)=J_{0}\left(\exp\left(\frac{qV_{\text{OC}}}{n_{\text{ID}}k_{B}T}\right)-1\right)-J_{G}\left(I\right)$$

with recombination current *J*_R_, the generation current density *J*_G_, light intensity *I*, the elementary charge *q*, the externally applied *V*_OC_, the Boltzmann constant *k*_B_, the temperature *T*, and the ideality factor *n*_ID_. The PSCs present a *n*_ID_ between 1 and 2. If the *n*_ID_ is close to 1, the recombination is dominated by free carrier band‐to‐band radiative recombination; while *n*_ID_ is close to 2, and the recombination is dominated by nonradiative recombination.

From the analysis of the ideality factor, the performance of most ETL/HTL‐free PSCs is limited by nonradiative recombination at the MHP/electrode contact. Figure 12e shows the dependence of *V*_OC_ on light intensity for PSCs with FTO/MAPbI_3_ contact and the BIPH‐II modified FTO/MAPbI_3_ contact, revealing a reduced *n*_ID_ from 1.7 to 1.2.^[^ ^103^ ^]^ The reduced ideality factor indicates that the dominant carrier recombination inside the device changes from nonradiative recombination to radiative recombination when the ITO/MHP interface is modified. As a result, the interface modification by BIPH‐II reduces the nonradiative recombination at the interface, which is the dominant recombination mechanism. The nonradiative recombination at the contact is further supported by comparing the ideality factors of full‐structure PSCs and ETL‐free PSCs. It was found that the ideality factor of the full‐structure PSC approaches 1.26, indicating band‐to‐band radiative recombination. The weak nonradiative recombination in full‐structure PSCs ensured that the device had a high *V*_OC_ and fill factor. However, the ideality factor was 1.65 for ETL‐free PSCs, suggesting that nonradiative recombination plays a dominant role in the ETL‐free PSCs.^[^ ^181^ ^]^

The origin of the surface nonradiative recombination of the MHP/TCO remains a debated topic. From the dark *J*–*V* measurement, the trap density can be extracted by a trap limited voltage (*V*_TFL_) indicating the current behavior transition from “ohmic” current to space‐charge‐limited current. It was found that the *V*_TFL_ of the full‐structure PSC is 0.20 V, smaller than that 0.32 V of the ETL‐free PSC. The trap‐state density is determined though

(5) $$n_{\mathrm{trap}}=\frac{2\varepsilon \varepsilon_{0}V_{\mathrm{TFL}}}{qL^{2}}$$

where *L* is the thickness of the MHP layer, *q* is the elementary charge, *ε*_0_ and *ε* are the vacuum permittivity and dielectric constant. Trap‐state density is increased from 0.44 × 10^16^ to 0.71 × 10^16^ cm^−3^ by removing the ETL layer.^[^ ^181^ ^]^ It is revealed that only shallow defects form in both the bulk and the surface of the MHP layer. However, the chemical distinction of the contact from the bulk could induce deep traps at the MHP/TCO contacts, which is detrimental to the properties of ETL/HTL‐free PSCs.^[^ ^39^, ^214^, ^215^ ^]^ As shown in Figure 12f, Xu et al. explored the interface chemistry of the evaporated MHP films on ITO. An inevitable PbI_2_ phase formed due to the low binding energy of adsorbed MAI particles to the ITO substrate, leading to a surface trap state at the interface.^[^ ^216^ ^]^ Thus, the increased trap‐states density by removing ETL could attribute to the MHP/TCO contact. For most ETL/HTL‐free PSCs, charge carrier recombination at the contact overrides the recombination in the bulk of the MHP films. The Shockley–Read–Hall recombination mechanism describes the typical nonradiative recombination.

There is another possible nonradiative recombination channel at the MHP/TCO contact. Back‐recombination is the recombination of the electron with the hole accumulated at the contact. It has been reported that the back‐recombination is an important factor limiting the performance improvement of the ETL/HTL‐free PSCs.^[^ ^71^, ^201^ ^]^ However, it is difficult to distinguish back‐recombination from the recombination of trap states at the contact. In response to this issue, the surface of the MHP layer is usually passivated in various ways, for example, the incorporation of addictive metal ions, exposure of the MHP boundary to oxygen ions,^[^ ^217^ ^]^ or introduction of various organic layers.^[^ ^207^, ^218^, ^219^ ^]^ Thus, the engineering of the contact is generally believed to be effective in reducing the nonradiative recombination at the contacts.^[^ ^41^ ^]^

### 4.3 *V*_OC_ and the MHP/TCO Contacts

In general, the *V*_OC_ of the solar cell is determined by the band structure and the carrier recombination of the semiconductor. The band structures of the semiconductor, along with the radiative recombination and nonradiative recombination, set an upper limit for the PCEs of solar cells. In this case, the *V*_OC_ equals the quasi‐Fermi level splitting (QFLS), which could be determined through the PL measurement. Recently, the PL measurements on neat MHP/layer, MHP/TL, MHP/electrode, and even full‐structure solar cells have been used to determine various recombination losses on the *V*_OC_. This methodology is based on the relationship between QFLS and fluorescence quantum yield (PLQY)

(6) $$\mathrm{QFLS}={\mathrm{QFLS}}_{\mathrm{rad}}+k_{B}T\times \ln\left(\mathrm{PLQY}\right)$$

where QFLS_rad_ is the maximum achievable, QFLS is the radiative limit of the MHP material. Hence, QFLS_rad_ is the maximum achievable *V*_OC_ in the case of pure radiative recombination.^[^ ^194^ ^]^ This way, the radiative limit of the *V*_OC_ in MAPbI_3_ is 1.32 V, with band gaps of *E*_g_ about 1.6 eV.^[^ ^199^, ^220^ ^]^ Recently, the QFLS of MAPbI_3_ films on glass has shown a value of 1.28 V, 0.04 V smaller than the radiative limit value, indicating that the bulk materials have a certain capacity to overcome nonradiative recombination in bulk. Also, a *V*_OC_ exceeding 1.26 V has been demonstrated in inverted planar MAPbI_3_ solar cells by suppressing surface and bulk recombination.^[^ ^83^ ^]^

The quasi‐Fermi level aligns with the opposite electrodes, producing a QFLS bending along the device. In this case, the *V*_OC_ is determined by the difference in quasi‐Fermi levels between the anode and cathode.^[^ ^194^ ^]^

#### 4.3.1 *V*_OC_ and Energy Band Alignment

**Figure 13:**
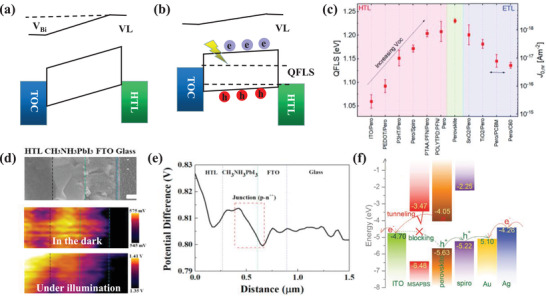
a) Band diagrams of ETL‐free PSCs in short‐circuit condition and b) in open‐circuit condition under light illumination. c) The calculated quasi‐Fermi level splitting (QFLS) of MHP contact with different substrates from absolute PL measurements. Reproduced with permission.^[^ ^213^ ^]^ Copyright 2019, Royal Society of Chemistry. d) The cross‐sectional scanning electron microscopy image and surface potential images in the dark and under illumination of HTL/MAPbI_3_/FTO/Glass. e) The difference of linear surface potential profiles of HTL/MAPbI_3_/FTO/Glass in the dark and under illumination. Reproduced with permission.^[^ ^181^ ^]^ Copyright 2019, American Chemical Society. f) Energy level diagram of ITO/MHP/Spiro/metal (Au, Ag) with an MSAPBS modified ITO. Reproduced under the terms of the Creative Commons CC‐BY license.^[^ ^75^ ^]^ Copyright 2020, The Authors. Published by Wiley‐VCH.

The metal–insulator–metal picture reasonably describes the vertical thin‐film MHP‐based devices and the origin of *V*_OC_.^[^ ^221^, ^222^, ^223^ ^]^ In the picture, a built‐in potential is established, which amounts to the difference between the work functions of metals. As shown in **Figure** **13**a, the built‐in potential of ETL‐free PSCs is determined by the difference in work functions between the TCO and doped HTL (the Fermi level is near the valence band). The metal–insulator–metal picture is consistent with the Schottky contact band structure, where the MHP layer is fully depleted (a uniform internal electric field is determined by the applied voltages and the built‐in electric field).

Under illumination (Figure 13b), the mismatch of the energy levels, the Fermi energy of TCO, and the conduction band of MHP, gives rise to the loss of QFLS at the electrode, which has been used to explain the *V*_OC_ loss. The simulation of *V*_OC_ and QFLS also confirmed this in ETL‐free PSCs using SCAPS‐1D software.^[^ ^26^, ^47^ ^]^ The simulated *V*_OC_ is identical to the QFLS injunctions with an aligned energy band. However, when the Fermi level of TCO mismatches with the conductive band of the MHP layer, the simulated *V*_OC_ of the device reduces. The energy offset at the contact leads to large QFLS bending to align with the Fermi energy of the electrode. As a result, significant *V*_OC_ losses are induced by the accumulation of charge carriers at the contact, increasing the interfacial recombination. Figure 13c presents the calculated QFLS of the MHP contact with different substrates from absolute PL measurements.^[^ ^47^ ^]^ It could be found that the MHP layer showed a QFLS of 1.23 eV, while the MHP/ITO contacts showed a QFLS of ≈1.06 eV due to the energy level mismatch. Thus, eliminating energy offset is essential to achieving energy level alignment and promoting *V*_OC_ of the ETL/HTL‐free PSCs.^[^ ^224^ ^]^

Scanning Kelvin probe microscopy (SKPM) has been employed recently to detect the real‐space internal potential, energy band, and charge carrier built up at the contact by measuring the surface potential image of the PSCs cross‐sections.^[^ ^187^, ^189^, ^225^ ^]^ During the SKPM measurement, the difference in surface work functions is determined by the local contact potential difference (CPD) between sample and tip with a resolution of nanometer

(7) $$qV_{\mathrm{CPD}}=\phi_{\mathrm{tip}}-\phi_{\mathrm{sample}}$$

where *φ*_tip_ is the work function of the tip and *φ*_sample_ is the sample's work function, and *q* is the element charge. When the work function of the tip is fixed during the scanning of the sample, the CPD reflects the distribution of electrical potential throughout the internal interface.^[^ ^204^ ^]^

SKPM measurements have given factual electrical information at the contact. When the MHP‐based solar cells are scanned in short‐circuit conditions and the dark, the CPD reflects the built‐in potential of the device. Bergmann et al. have found a decreasing CPD of the MHP film from the FTO (0.6 V) to the Au electrode (0 V) during the SKPM scanning on the cross‐section of the device with a configuration of ITO/TiO_2_/MHP/HTL/Au. The difference between the two CPD values corresponds to the work function difference between FTO (4.4 eV) and Au (5.1 eV).^[^ ^225^ ^]^ The CPD measurement gives a similar CPD distribution when MHP is in contact with the Au electrode (for HTL‐free PSCs, the MHP is in contact with the Au electrode). These results indicate that a uniform built‐in electric field is present with the difference in electrode work functions, as illustrated by the metal–insulator–metal picture, and the MHP film does not have enough impurities to induce the band bending at the contact.

Charge carriers can be induced by doping or interface dipoles in the MHP‐based solar cells, which can reconfigure the energy band structure at the interface, resulting in the redistribution of built‐in potential. In the SKPM measurement of HTL/MHP/FTO structure with the doping density is 3.94 × 10^14^ cm^−3^, the variable CPD is observed across the MHP layer in Figure 13d, illustrating that a built‐in electric field at the FTO/MHP interface.^[^ ^181^ ^]^ The result suggests that the energy band alignment at the FTO/MAPbI_3_ contact allows the MAPbI_3_ band to bend direction downward. Correspondingly, the built‐in electric field at the interface points from FTO to MAPbI_3_ can attract electrons and repel holes. A similar structure of TiO_2_/MAPbI_3_ can also be measured by the SKPM CPD method. The doping density of the MHP layer was about 1.8 × 10^17^ cm^−3^. It reveals that the depletion width within the MHP layer is approximately half of the MHP layer thickness (≈300 nm), leaving a significant neutral zone around the anode contact.^[^ ^188^ ^]^ Thus, the MHP doping can reconfigure interfacial energy band structure and the distribution of built‐in potential.

Except for doping, the SKPM measurement has proved that the electronic structure and the magnitude of built‐in potential are sensitive to the dipoles at the contact. This unique property allows modifying of the built‐in potential for the MHP‐based PSCs by introducing surface dipoles and ultimately improving the *V*_OC_. Zhang et al. revealed that enhanced built‐in potential was realized by a dipole interlayer assembled by the Lewis‐acid‐featured fullerene skeleton after iodide ionization (PCBB‐3N‐3I) with a strong molecular electric dipole.^[^ ^189^ ^]^ As can be seen, the electronic structure reconfiguration by introducing dipoles can be applied to the MHP/TCO contacts.

Under illumination, the mismatch of the energy levels causes charge carrier accumulation, which changes the distribution of the internal potential at the contact. Wang et al. compared the potential distribution across the layers of FTO/MAPbI_3_/HTL by measuring the cross‐section via SKPM of samples in the dark and illumination, respectively (Figure 13d). Under illumination, they found substantial surface potentials were changed, which were primarily produced over the region of the MHP/FTO contact by calculating the difference in line profiles of the potential distribution for devices in the dark and illumination (Figure 13e), implying that the interface charges accumulated at the surface.^[^ ^181^ ^]^ Due to the unbalanced charge transport, the charge accumulated in contact, resulting in the unmatched collection efficiency of the two electrodes for the corresponding carriers. Bergmann et al. found that the built‐in field was not uniform as CPD increased up to 0.4 V in the MHP layer of the ITO/TiO_2_/MHP/HTL/Au device upon illumination and in short‐circuit conditions by SKPM. This increase in CPD corresponded to the accumulation of positive charge carriers and demonstrated that the extraction of electrons in the TiO_2_ was more efficient than the extraction of holes in the HTM.^[^ ^225^ ^]^ The accumulated charge increased backward charge recombination current and the loss of the QFLS and *V*_OC._

#### 4.3.2 *V*_OC_ Losses at the Contact

For ETL/HTL‐free PSCs, one of the challenges to improving their performance is to approach the radiative limit of *V*_OC_ by minimizing nonradiative recombination processes at the contact. The *V*_OC_ losses at the MHP/TCO contact were revealed by comparing QFLS in the MHP bulk obtained and measuring the emitted PLQY on MHP/TCO contacts and glass substrate.^[^ ^213^ ^]^ The interface nonradiative recombination reduced PRQY and led to *V*_OC_ loss in the form given by Equation (6).^[^ ^213^ ^]^ As shown in Figure 13c, the QFLS is ≈1.23 eV, which is only 0.11 eV lower than the radiative *V*_OC_ limit. The presence of ETL/HTL or metal substrate causes the decrease of QFLS due to interfacial recombination. The MHP/ITO junction shows a QFLS of 1.06 eV, demonstrating a large number of carrier recombination at the interface. The nonradiative recombination occurs mainly at the MHP/HTL (ETL) interfaces, suggesting that the interfacial recombination currents set a limitation on the *V*_OC_ and ultimately limit the performance of their cells. Wu et al. extended this approach to obtain the pseudo *J*–*V* curves by measuring the intensity dependence of the QFLS between the isolated MHP absorbing layer and MHP layers on transport layers or electrodes. This powerful approach enables the quantification of interfacial nonradiative recombination on the *V*_OC_ and fill factor.^[^ ^47^ ^]^ Stolterfoht et al. highlighted that the threat to restrain nonradiative recombination losses of PSCs primarily depends on the energy level alignment and the suppression of recombination at the contacts.^[^ ^213^ ^]^

The analysis suggests that the properties of ETL/HTL‐free PSCs are mainly limited by three factors at the interface between the electrode and MHP.

1) Poor efficiency of charge carrier transfer from MHP to TCO brings more trap‐assisted carriers to interface recombination.
2) The energetic offsets cause *V*_OC_ losses beyond the limitation imposed by trap‐assisted and radiative recombination.
3) The trap‐assisted recombination at the MHP/TCO contact causes significant *V*_OC_ losses and fill factors in ETL/HTL‐free PSCs.

As a result, it puts forward the direction and requirement for interface modification of devices with MHP/electrode contact. As shown in Figure 13f, by employing a wide bandgap and MSAPBS film between ITO and MHP, Huang et al. made an ETL‐free PSC with a PCE of as high as 20.55%.^[^ ^75^ ^]^ The MSAPBS nanolayer reduced the effective work function of ITO by 0.97 eV, forming matched energy level alignment (ohmic contacts) between the MHP and ITO, which facilitated electron extraction and hole blocking from the MHP to the ITO electrode. Moreover, the MSAPBS nanolayer could also passivate the interface defect and inhibit interface charge accumulation, promoting chemical stability, and decreasing the hysteresis effect.

### 4.4 Interface Engineering of MHP/TCO Contact

MHP/TCO surface or interface engineering is used to optimize the performance of electronic devices, including solid‐state dye‐sensitized solar cells, organic solar cells, etc. Modifying TCO glass substrates could regulate the surface work function and form better ohmic contacts between substrates and active layers. Recent studies have proved that surface or interface engineering approaches could avoid the deposition of transport layers to effectively simplify device structure and reduce the fabrication cost of electronic devices. Herein, we have summarized MHP/TCO interface engineering, including the physical and chemical engineering, to understand how the surface or interface contact affects the performance of electronic devices.

#### 4.4.1 Physical Engineering for the TCO Substrates

**Figure 14:**
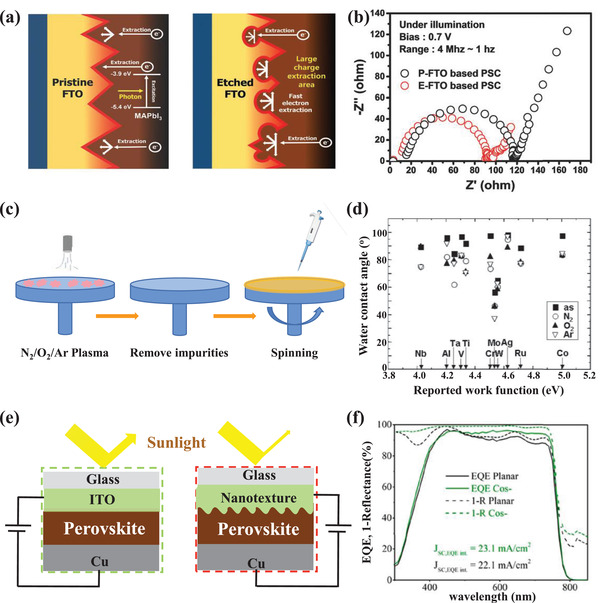
a) Schematic illustration of the enlarged MHP contact to the FTO through electrochemical etching (E‐FTO) and larger charge extraction than the pristine FTO (P‐FTO). b) The reduced contact resistance revealed by Nyquist plots of the PSCs with the P‐ and E‐FTO substrates measured under illumination. Reproduced with permission.^[^ ^97^ ^]^ Copyright 2017, Wiley‐VCH. c) Schematic illustration of pretreatment of the TCO substrate by N_2_, Ar, or O_2_ plasma. d) The water contact angle change of various metals, after treatment with N_2_, Ar, or O_2_ plasma. Reproduced with permission.^[^ ^226^ ^]^ Copyright 2008, American Institute of Physics. e) Schematic representation of the light management of PSCs with nanotextured (right) ITO substrate compared to planar (left) substrates. f) External quantum efficiency (solid line) and 1‐reflectance (1‐R) (dashed line) of PSCs employing ITO substrates with different nanotextures. Reproduced with permission.^[^ ^227^ ^]^ Copyright 2020, American Chemical Society.

For most ETL/HTL‐free PSCs, the MHP films are directly deposited on the TCO substrates. Depositing the MHP films using a solvent system is relatively difficult on a hydrophobic and smooth surface. Therefore, the physical premodification of the TCO substrates has a substantial impact on the morphology at the interface and the properties of the MHP film, such as the grain aspect ratio and the coverage on the TCO or contact areas. Various methods, including O_2_‐plasma treatment, UV‐ozone, wet‐etching, electrochemical etching, photoresist, etc., were utilized in different cases. These physical methods aim to remove contamination, enhance the roughness or wettability of the TCO substrate surface, and further optimize the properties of electronic devices. **Figure** **14** summarizes the influence of physical strategies on the TCO substrate surface in electronic devices, which can be divided into three sections, including

1) the increase of contact surface area between the TCO and MHP, and correspondingly the decrease of obstacles for photoexcited electron transport,
2) the improvement of MHP film quality, and
3) better optical transmittance because of the increased absorption and decreased reflection losses.

A porous FTO structure (etched FTO) by etching can substantially increase contact surface area, effectively reducing the charge transfer resistance at the interface in PSCs. Thus, the etching of FTO is an alternative approach to electron engineering. As shown in Figure 14a, the modified FTO surface to a porous structure via electrochemical etching contributes to a significant increase in the photocurrent density because of an increased interface area.^[^ ^97^ ^]^ The contact resistances for charge transfer with the pristine FTO and etched FTO in PSCs have been measured by EIS in the illumination condition. As shown in Figure 14b, the etched‐FTO‐based PSC exhibits a smaller main arc than the P‐FTO‐based PSC, presenting a reduced charge transfer resistance at porous FTO/MHP contact. This decreased resistance is caused by the preferable contact of the MHP with the etched FTO due to its better wettability and higher surface area than the pristine FTO.^[^ ^97^ ^]^ Apart from electrochemical etching, wet‐etching of self‐agglomerated Ag nanoparticles on ITO has been used to increase the MHP/TCO contact surface area. A nanoporous structure formed by removing the embedded Ag nanoparticles on the ITO surface, led to an increased contact area and optical transmittance of 89.08% at 550 nm. Ultimately, the fabricated PSCs with nanoporous‐surface ITO showed a higher PCE of 20.1% and a fill factor of 81.1% compared to PSCs with the pristine ITO.^[^ ^228^ ^]^

Perovskite films with large grain and smooth surfaces are the desired film morphology. A large grain MAPbI_3_ film could interrupt the penetration of HTL into FTO in the absence of ETLs, which reduces the leakage current of ETL‐free PSCs.^[^ ^97^ ^]^ The large grain also increases carrier mobility and reduces recombination. For one reason, the perovskite film with larger grains has fewer boundaries and reduces defect density, which causes the Shockley–Read–Hall monomolecular recombination. The decreased recombination rate in films with larger grains has been verified by enhanced PL emission.^[^ ^97^ ^]^ Moreover, large grains can reduce the ion migration routes, modify the undesired hysteresis, and hinder moisture penetration.^[^ ^97^ ^]^

The surface energy of the TCO glass, measured by the contact angle of water, influences the grain aspect ratio, grain size, and crystallinity. The substrate surface is more hydrophobic (a larger contact angle) and would have a larger grain film. The surface of aluminum‐doped zinc oxide (AZO) is the most hydrophobic with the smallest surface energy, possessing the smallest surface roughness and best crystal sizes as the MHP films compared to common glass substrates.^[^ ^229^ ^]^ The morphologies of MAPbI_3_ films prepared on ITO, AZO, and FTO have grain sizes of ≈100–150, 400–500, and 200–300 nm, respectively. The surface energy can be regulated by physical engineering for the TCO substrates. Figure 14c illustrates the pretreatment of the TCO substrate by plasma before coating the MHP layer, including N_2_/O_2_/Ar plasma. It was pointed out that a plasma‐cleaning pretreatment for FTO substrates can effectively enhance the quality of the MHP layer, promote charge separation, increase the electron transfer rate, and reduce the recombination at the MHP/FTO contact.^[^ ^95^ ^]^ Figure 14d illustrates that the N_2_/O_2_/Ar plasma can reduce surface contamination and make the metal surface hydrophilic, increasing the adhesion of the metal surface.^[^ ^226^ ^]^ Moreover, various methods exist to obtain perovskite film with larger grain sizes, such as solvent annealing, additive components, etc. Further details on the deposition of perovskite thin films, crystal growth techniques, and fundamental nucleation theories are discussed elsewhere.^[^ ^226^ ^]^

Planar PSCs always suffer from unavoidable light reflection losses, although the thicknesses of all the layers are optimized. Light management can increase the energy conversion efficiency of PSCs by reducing the reflection losses and thus increase light absorption in the perovskite. As shown in Figure 14e, advanced light management is usually applied by introducing appropriate micro‐ and nanotextures, which could contribute to a broadband reduction of reflection losses of light. The antireflective effect of microtextures is explained by light trapping (bouncing back the reflected light onto the MHP surface) and the graded effective index induced by nanotextures with a suitable conical shape. To optimize the transmittance of light, ITO substrates have been etched differently. ITO substrates with porous structures, such as a positive (“Cos+”) and a negative (“Cos−”) (feature heights of 220 and 380 nm), a pyramidal structure (a depth of about 600 nm and period of 1250 nm), and hexagonal pillar‐like nanotextures (a height of around 350 nm and a period of 750 nm), were formed. Then, PSCs were prepared on these ITO substrates with MHP films of similar grain size and morphology. As shown in Figure 14f, the external quantum efficiency (EQE) reached a plateau for the devices on nanotextures between 450 and 730 nm, demonstrating that the obvious thin‐film interference dips can be avoided. Moreover, the EQE close to the band edge increased in sinusoidal and inverted pyramid devices.^[^ ^227^ ^]^ The ITO etching was carried out by using samples that were partly covered with a photoresist. Then, ITO reacted with Brønsted acids with strength ranging from concentrated HCl to dilute carboxylic acid solutions. It demonstrated that ITO could be easily etched by MAI, MAPbI_3_, and FAPbI_3_‐based MHPs, leading to the formation of In^3+^ ions.

#### 4.4.2 Chemical Engineering for TCO Substrates

**Figure 15:**
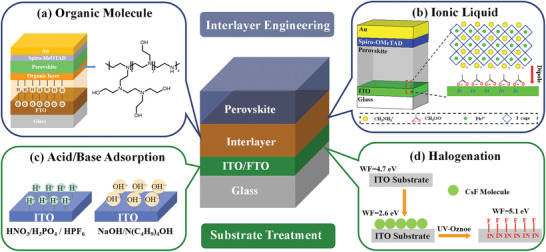
a) Schematics illustration of energy band of the contact modified by PEIE, which shows a band bending of the MHP. b) Schematic illustration of the in situ modification of the MHP/ITO contact utilizing the methylammonium acetate ionic liquid‐based MHP precursor. c) Schematics of the adsorption of monolayer of acids (left) and bases (right) onto the ITO surface. d) Schematic illustration of the effect of polar In—F bonds on the ITO substrate.

Chemical adsorption or deposition of functional layers on TCO is a standard interface modification method, which has been widely used to adjust the work function of substrates. It will lead to favorable band bending and charge carrier processes at the MHP/TCO contacts. According to the types of materials and adsorption mechanism, we classified chemical surface and interface modification of glass substrates into four categories as exhibited in **Figure** **15**: a) self‐assembled nonconductive polar small‐molecule compound and polymers, b) incorporation of ionic liquid, c) the adsorption of acid and bases, and d) the halogenation of TCO substrates.

Self‐assembled, nonconductive, and polar small molecules and polymers have been studied for the chemical engineering of glass substrates. The work function of TCO is adjusted by forming a chemical bond between the polar small molecules or polymers and the ITO surface. The device's performance can be obviously enhanced. Huang et al. spin‐coated polar nonconjugated small‐molecule layer MSAPBS on ITO substrates, and the MSAPBS molecule layer was chemically bound to the ITO substrate by strong S—In and S—Sn bonds. The work function of ITO was decreased from 4.70 to 3.73 eV because of the intrinsic dipole moment of the molecules. The decreased work function of ITO made the MHPs band‐bending downward. The energy offset between Fermi energy of ITO substrate and the conductive energy band of the MHP layer was dramatically decreased from 0.65 to 0.05 eV, while the energy offset between the Fermi energy of ITO and the valence energy band of the MHP layer was increased from 0.93 to 1.63 eV. The changes in the energy offsets improved the efficiencies of electron extraction and hole blocking at the ITO/MHP contact. Ultimately, the charge recombination and energy loss were accordingly inhibited, leading to the enhanced *V*_OC_, short‐circuit current, and fill factor. Accordingly, the efficiency of the modified ETL‐free PSCs reached 20.55%.^[^ ^75^ ^]^ Another approach to reducing the work function of TCO is introducing an ultrathin polymer layer (from 1 to 10 nm) with simple aliphatic amine groups, for example, PEIE and branched PEI. As shown in Figure 15a, these polymers are adsorbed onto the TCO conductor surface by aliphatic amine groups, inducing intrinsic molecular dipole moments at the top of the substrate. The spectra revealed that work functions of ITO reduced from 4.40 to 3.30 eV once adding an ultrathin layer of PEIE, exhibiting certain stability over four weeks in ambient conditions.^[^ ^230^ ^]^ Song et al. employed PEIE to modify the MHP/FTO contact interface and fabricated ETL‐free PSCs with an improved PCE. The dipole moment was introduced at the FTO/perovskite contact by the self‐organized PEIE interlayer. And a favorable contact with excellent electron extraction was yielded.^[^ ^100^ ^]^ These progress highlight that interface modification can significantly improve the performance of devices with TCO/MHP contact.

The hydroxy groups contribute to the chemical adsorption of ionic liquid onto the surface of TCO substrates. The self‐assembly of ionic liquid with functional groups tends to form a uniform and compact ultrathin film, which may produce a dipole layer and affect the work function of TCO substrates. Cheng et al. designed and prepared a BIPH‐II ionic liquid and applied the ionic liquid to a modified FTO conductive substrate. They measured X‐ray photoelectron spectroscopy (XPS) high‐resolution survey spectra of C 1s and O 1s and deduced that strong chemical bonds were formed via interaction between the hydroxy end group of ionic liquids and the surface of the FTO substrate. Finally, the PCE of ETL‐free PSCs with self‐assembly ionic liquid on the FTO substrate improved from 9.01% to 17.31%.^[^ ^103^ ^]^ Recently, Huang et al. reported an in situ interface engineering strategy by utilizing the methylammonium acetate ionic liquid‐based MHP precursor (Figure 15b). It was worth noting that the ionic liquid meanwhile acted as the outstanding processing solvent. They presented detailed information on the effect of the adsorption of methylammonium acetate on the ITO substrates. The adsorption of the polar methylammoniumacetate on the ITO surface induced a surface dipole, decreasing the work functions of ITO from 4.42 to 4.16 eV via XPS and first‐principles simulations. The reduced work function enabled in situ band bending in the perovskite semiconductors, facilitating charge collection and hindering interfacial charge recombination. Ultimately, the simple in situ strategy produced ETL‐free PSCs with a maximum PCE of 21.08%.^[^ ^26^ ^]^ Nuesch and co‐workers systematically research on the chemical adsorption of acids and bases on ITO. ITO substrates were immersed with different acids, such as H_3_PO_4_, HNO_3_, HPF_6_, to protonate the surface, leaving anions to assemble on top of the substrate (Figure 15c), forming a surface dipole and promoting the work function. The ITO samples treated with bases, such as NaOH or N(C_4_H_9_)_4_OH, exhibited an opposite ionic double layer compared to those treated with acids due to the bases dissociated into surface‐bound hydroxyl groups and the corresponding cations (Figure 15c).^[^ ^231^ ^]^

Halogenation is usually used to modify the substrate surface to improve interface energy level alignment, promoting more efficient electron‐selective contact. Lu et al. modified the ITO substrates by soaking in CsF solutions with various concentrations and measured their surface energy, surface polarity, and work function of ITO. The work function of the modified ITO substrate was 0.33 eV higher than that of the base ITO substrate. Figure 15d illustrates that immersion of ITO substrates in the CsF solution promotes the formation of In—F bonds due to these fluorine atoms chemically bonded onto the ITO surface.^[^ ^232^ ^]^ Research on chlorination of ITO has also been reported, such as indium trichloride evaporation, Cl_2_‐plasma etching, and chloroform treatment.^[^ ^233^, ^234^, ^235^ ^]^ To reveal the detailed process of halogenation, Huang et al. studied the interaction of F and Cl on the ITO surface via density functional theory calculations and proved that the dramatic increase of the work function was derived from the strong surface dipole rather than the change of the electrochemical potential of ITO.^[^ ^236^ ^]^ The FTO substrate can also be chlorinated by o‐dichlorobenzene, increasing the work function from 4.7 to 6.1 eV. Deng et al. have proved that a chlorinated FTO electrode was effective in optimizing the PSC with PCE enhancement of 49%, compared with plain FTO‐based PSCs.^[^ ^237^ ^]^ These works suggest that halogenation is an effective way to tune the work function of TCO.

## 5 The Contacts of MHP and Metals

The interactions between metals and MHP have initially attracted much attention because of their decisive impact on the stability of PSCs. In the presence of a barrier layer, the decomposition of MHP film and the corrosion of metal electrodes caused by the diffusion or penetration of material components are important factors leading to the aging of devices. When contact with the unstable MHP, the active metal degradation is more serious. Therefore, a long time maintaining the fragile contact with layered structures of several hundred nanometers is the primary concern to ensure the stability of the MHP‐based devices without barrier layers. However, it is not easy to prevent the fusion of the metal electrode and the MHP layer. For example, some of the top electrode metals directly attached to MHP are thought to break the balance of degradation and regeneration of MAPbI_3_ through reversible reactions^[^ ^60^ ^]^

(8) $${\mathrm{MAPbI}}_{3}↔{\mathrm{PbI}}_{2}+{\mathrm{CH}}_{3}{\mathrm{NH}}_{2}+\mathrm{HI}$$

(9) $${\mathrm{MAPbI}}_{3}\overset{hv}{⟷}\mathrm{Pb}+{\mathrm{CH}}_{3}{\mathrm{NH}}_{3}I+I_{2}$$

The instability and plasticity of MHP/metal contact lead to new properties of MHP‐based devices, such as memristive and resistive switching effects. In the last few years, the research on these properties has accelerated the development of devices with MHP/metal contacts, such as memristors and artificial synapses. Researchers are trying to clarify the detailed mechanism of these effects. Various models have been proposed, which involve the migration of both ions and metal atoms. Metal electrodes can “extract” ions from the MHP and accelerate the decomposition of the material and the corrosion of metal electrodes.^[^ ^238^ ^]^ Moreover, some metals, Ag and Al, could penetrate deep into the MHP film and form carrier recombination centers or conductive filaments, explaining the working principles of MHP‐based memristors.^[^ ^19^, ^239^ ^]^ Thus, an inert metal/MHP‐contact is required by MHP‐based devices, while a well‐controlled and chemically flexible metal/MHP‐contact is favorable. However, the fundamental principles of the metal/MHP contact that generally determine device operation are unclear. In this section, we hope to put forward the problems of MHP/metal contact via a summary of the recent relevant results.

### 5.1 Chemical Reaction at the MHP/Metal Contacts

The MHP/metal contacts involve abundant interfacial chemistry processes endowing the structure with many new features. MHP reacts strongly with the frequently‐used metal electrodes, such as Ag,^[^ ^242^ ^]^ Al,^[^ ^239^ ^]^ and Au,^[^ ^82^ ^]^ which constitutes a grand challenge for long‐lived MHP‐based devices. The chemical reactions at the MHP/metal contacts lead to the corrosion of metal materials, the decomposition of MHP, or both. The chemical reaction is closely connected with environmental factors, for instance, light, humidity, temperature, and applied voltages. However, the research on chemical reactions at the MHP/metal contacts is still in the early stage. The lack of understanding of these interfaces limits the development of related stable MHP‐based devices. In this section, we have summarized the chemistry of the contacts of MAPbI_3_ and used metal materials, such as Ag, Au, Al, and Cu.

#### 5.1.1 Chemical Reactions of the MHP/Au Contacts

As a noble metal, Au is commonly used as the electrode material for MHP‐based devices. MHP/Au contact is the crucial part of the MHP‐based diodes, and field‐effect transistors display current–voltage hysteresis, memristive effects, giant switchable photovoltage, space charge limited current, or injection limited current. One reason is that Au is chemically and photochemically stable. When Au and the MHP layer are in contact, there are no direct interfacial chemical reactions. Moreover, the resistance caused by the oxide layer does not exist at the MHP/Au contact. The chemical resistance is due to its high ionization potential (9.2 eV). By contrast, Ag and Cu have ionization potentials of 7.6 and 7.7 eV, respectively. As a result, a stable Schottky barrier at the MHP/Au contact is justified to explain that holes can transfer from MHP to Au electrodes while electrons can be blocked. From this point, Au can be replaced by another noble metal Pt because the work function of Pt (5.65 eV) is slightly higher than that of Au (5.4 eV), and it also has higher ionization energy of 9.0 eV.^[^ ^75^, ^82^ ^]^ Actually, HTL‐free PSCs with Pt electrode have similar properties to HTL‐free PSCs with Au electrode, indicating that Pt works well as the electrode of MHP‐based devices. However, there are few reports on the related devices up to now.

**Figure 16:**
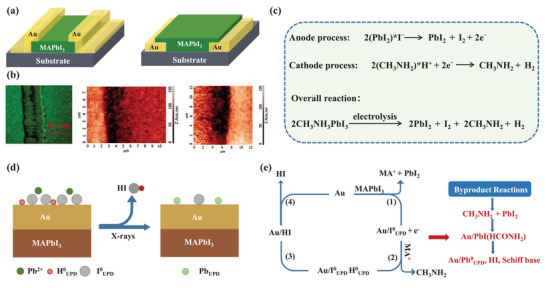
a) Schematic illustration of lateral devices with the top contact and bottom contact of Au electrode with MAPbI_3_ films. b) AFM images illustrating degradation of the perovskite layer nearby the Au anode and cathode. c) Schematic illustration of the MAPbI_3_ decomposition under applied electric field. Reproduced with permission.^[^ ^240^ ^]^ Copyright 2015, The Royal Society of Chemistry. d) Schematic illustration of the UPD adsorbed species (a hashed fill) and the evolution of the UPD layer on the Au surface for MAPbI_3_/Au during XPS measurements. e) Schematic illustration of the proposed Au catalyzed degradation at MAPbI_3_/Au contact during XPS measurements where the byproducts methylamine and PbI_2_ (in red) lead to the eventual formation of Pb^0^. Reproduced with permission.^[^ ^241^ ^]^ Copyright 2019, Author(s). Published by AIP Publishing LLC.

Although MHP/Au contact shows chemical and photochemical stability, its electrochemical instability brings irreversible damage to the relevant devices. Upon voltage bias, MHP‐based transistors whose Au‐film electrodes were deposited on MAPbI_3_ or MAPbBr_3_ (**Figure** **16**a) would degrade faster than that without the voltage bias. The noticeable material bleaching was observed in a lateral structure of Au/MAPbI_3_/Au, with the MHP/electrodes using optical microscopy upon applying a voltage bias of 100 V (Figure 16b), suggesting the electrochemical reaction between Au and MAPbI_3_.^[^ ^240^ ^]^ The energy‐dispersive X‐ray spectroscopy revealed the composition change of perovskite films from one electrode to the other. However, there is no definite evidence for the chemical composition produced by the electrochemical decomposition of the MHP.

Two electrochemical reaction mechanisms have been proposed to demonstrate the electrochemical decomposition of the MHP at the MHP/Au contact. Frolova et al. proposed electrolysis of the MAPbI_3_ films with the production of I_2_, H_2_, PbI_2_, and CH_3_NH_2_ (Figure 16c). According to the electrolysis mechanism, I_2_ was liberated by the oxidation reaction of the I^−^ at the anode, while H_2_ was produced at the same time at the cathode due to the reduction reaction of the H^+^.^[^ ^240^ ^]^ Similarly, Kerner et al. showed that the electrochemical reactions occurred at the MAPbI_3_/Au contact forming Pb‐ and I‐species. They proposed an under potential deposition (UPD) reaction mechanism (Figure 16d) that was a surface adsorption‐assisted reaction in which the ions of the MHP changed the oxidation state after adsorption on the noble metal substrate. For example, the iodide adsorbed on the Au substrate, producing an electron and an I0 UPD. In the picture of UPD (Figure 16e), the degradation processes of MAPbI_3_/Au are as follows. 1) Once the adsorption of I0 UPD onto Au, electrons would release, and methylammonium and PbI_2_ are also produced. 2) The released electrons are captured by a proton from methylammonium adsorbed on the Au surface, and methylamine is therefore released. 3) The adsorbed I0 UPD and H0 UPD then react, and volatile HI, H_2_, and I_2_ release. 4) Subsequently, the PbI_2_ and methylamine byproducts react by proton transfer and Pb intermediates, and more volatile organic species, HI, and Pb0 UPD formed.^[^ ^241^ ^]^

The UPD mechanism is broadly consistent with the observations of the XPS data that revealed Pb^0^ is a product at MAPbI_3_/Au interfaces after the electrochemical reactions. It also indicates an electrochemical stability window for such devices. The measured values of the stability window are between −0.5 and 0.9 V. Except for these electrochemical stability window limits, rapid degradation of the devices seems unavoidable. However, the UPD reaction based on the adsorption mechanism depends on the Au electrode's exposed surface and MHP. Due to the accumulation of products, the decreasing contact indicates that the scope of this reaction is limited and unsustainable. By contrast, electrolysis of the MAPbI_3_ films is less dependent on electrodes. It is still a problem whether the electrolysis reaction happens at other MHP/electrodes contacts, such as MHP/ITO and MHP/FTO. If so, it will limit the development of MHP‐base devices, especially for those with MHP/electrode contact.

Electrochemical reactions at the MHP/Au contact lead to the long‐term degradation of the MHP‐based devices. The study of MHP and Au reaction has revealed that the electrochemical process of MHP/Au contact has no relevance with water and oxygen, which indicates that the device encapsulation does not affect the long‐term aging of the devices. Noble metals are not an ideal material for electrodes, at least for devices with MHP/electron contact for massive production. Moreover, the price of Au is higher than other electrode metals, such as Cu, Al, or Ag. Thus, we conclude that for MHP‐based devices, finding suitable electrode materials will become an interesting topic.

In addition to electrochemical reactions, polyiodide species exhibit strong reactivity to all kinds of metals like gold. It has been reported that the fast etching of gold electrodes was enabled by the chemical interaction between Au and polyiodide species that is prepared by I_2_ and KI in water via the “soft” acid–base reaction^[^ ^169^ ^]^

(10) $$I^{-}+I_{2}=I_{3}^{-}$$

It has been shown that polyiodide ions produced with MAI and I_2_ from MAPbI_3_ under UV irradiation. The polyiodide ions react with Au, leading to the [AuI_2_]^−^ and [AuI_4_]^−^ chemical species and the (MA)_2_Au_2_I_6_ phase via a further interaction with MA^+^ cations.^[^ ^169^ ^]^ This reaction makes Au vulnerable in contact with iodine‐containing systems under UV irradiation. However, compared with electrochemical reactions, the corrosion effect of Au and polyiodide species reaction on MHP/Au devices is still a problem.

#### 5.1.2 Ag‐Electrode‐Induced Chemical Degradation

Ag is a low‐cost electrode material that exhibits a good back electrode in PSCs. Ag is deposited on the surface of the device by vacuum thermal evaporation to form an alloy electrode. Besides, Ag mesh and Ag nanowires have been used as flexible electrodes by embedding them into the PET substrate after being coated with conductive polymers.^[^ ^243^, ^244^, ^245^ ^]^ If Au electrodes are replaced by the Ag electrode, the fabricated PSCs, such as FTO/TiO_2_/MAPbI_3_/Spiro‐MeOTAD/Ag, could work well, although the difference in the work functions is huge.

However, the contact of MHP to the active Ag electrode leads to the corrosion of electrodes and the decomposition of MHP layers. In most cases, Ag electrodes usually turn yellow within a few days when the device is manufactured due to the formation of AgI. Even in the HTL/ETL‐based devices, chemical reactions of the MHP/Ag contacts are ineluctable.^[^ ^82^, ^246^ ^]^ The formation of AgI has been observed in the MAPbI_3_/Spiro‐MeOTAD/Ag and MAPbI_3_/PCBM/Ag structures. This means that the HTM layer and the ETL layer cannot prevent the formation of AgI at the interface, even if they can isolate Ag from MHP and slow down the proportion of the corrosion.

**Figure 17:**
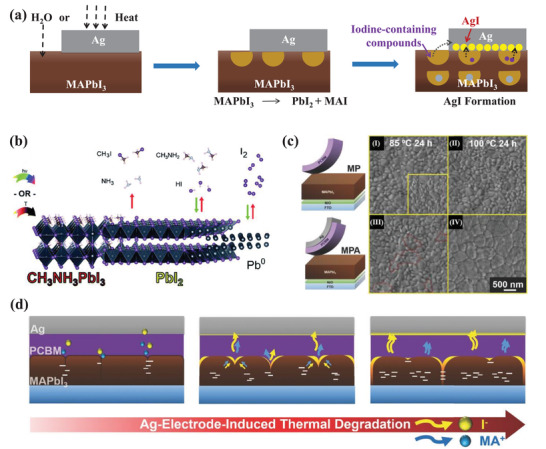
a) Schematic illustration of the proposed mechanism of decomposition of MAPbI_3_ and AgI formation at the MHP/Ag contact on exposure to light, heat and moisture. b) MAPbI_3_ photodecomposition and thermal degradation processes leading to irreversible decomposition to organic volatile gas species (CH_3_I + NH_3_), reversible decomposition (CH_3_NH_2_ + HI), and reversible generation of I_2_ and nonvolatile Pb^0^ under illumination or mild heat conditions. The irreversibility of the process to release CH_3_I and NH_3_ is indicated by a one‐directed arrow for the reaction. Reproduced with permission.^[^ ^60^ ^]^ Copyright 2018, The Royal Society of Chemistry. c) SEM images of the MAPbI_3_ thin films from (I, II) the MP substrates without Ag contact and (III, IV) the MPA substrates with Ag electrode after a thermal treatment at 85 or 100 °C for 24 h. d) Schematic illustration of the Ag‐electrode‐induced thermal degradation in the inverted PSCs with the diffusion and accumulation of I^−^ and MA^+^. Reproduced with permission.^[^ ^238^ ^]^ Copyright 2017, Wiley‐VCH.

Environmental factors influence the MHP/Ag contact, demonstrating a variety of chemical reactions on the interface. **Figure** **17**a shows the decomposition pathways of the Ag/MHP contact on exposure to moisture, light, and heat. It can be seen that the corrosion of the Ag electrode accelerated after exposing the MHP/Ag contact to air compared with the exposure to dry nitrogen. Under illumination, AgI, one of the decomposition products, was further decomposed, and the corroded electrode turned gray with the photosensitization of AgI. Thermally assisted degradation of MAPbI_3_/Ag contact was also revealed. And the decomposition of MAPbI_3_ became more significant in the air with a little water at 85 °C.

In general, Ag corrosion is accompanied by MHP decomposition, and there are two crucial chemical reactions, including the direct reaction of metal Ag with the I^−^ diffused into the electrode and the reaction of Ag with the iodine‐containing volatile species (MAI, HI, I^−^) from the decomposition of MAPbI_3_ induced by H_2_O, O_2_ or heat (Figure 17b). Kato et al. proposed a detailed mechanism of the chemical processes with MHP on exposure to moisture with five steps: 1) H_2_O molecule diffuses into the MAPbI_3_ MHP layer. 2) The decomposition of MHP produces iodine‐containing volatile species. 3) The iodine‐containing volatile species migrate from the MHP layer to the Ag electrode. 4) The iodine‐containing volatile species diffusion at the electrode surface. 5) The formation of AgI.^[^ ^242^ ^]^ Moreover, the thermal fluctuations at a high temperature can promote the iodine and MA to conquer the active energy and accelerate random diffusion, resulting in I^−^ and MA^+^. When these species diffuse into the Ag electrode, a corrosive reaction occurs. These reactions make the Ag electrode act as a pump to continuously extract iodine ions in the device, and the formation of AgI on the surface cannot slow down this process. This process can cause interesting microscopic changes in MHP morphology with varying temperatures. As exhibited in Figure 17c, Li et al. obtained the MHP thin film from the Ag‐based device by rinsing off the Ag electrode after thermal treatment at 85 °C for 24 h, and fused regions were present in the grain boundaries of the MHP thin film through the SEM image. The work showed the MHP morphology changing under continuous extraction of I^−^ via the formation of AgI produced by the reaction of Ag and I^−^.^[^ ^238^ ^]^ To further illustrate the experimental results, they proposed an Ag‐electrode‐induced thermal degradation at the Ag/MHP contacts (Figure 17d). First, I^−^ and MA^+^ depart from the MHP crystal lattice at the grain boundary and diffuse across any interlayers between Ag and the MHP. AgI near the Ag electrode acts as a pump for the I^−^ and MA^+^. Second, the I^−^ and MA^+^ loss reconstructs the grain boundary and visually “fused” large grain domain forms. Finally, a large number of ion losses promote the formation of lead iodide at the grain boundary, which separates the MHP polycrystals.

The work shows that the chemically active Ag contact is not an ideal choice for MHP‐based PSCs. The replacement of the Ag back electrode by developing chemically stable materials, including conducting polymers or carbonaceous materials, is a topic to be worth exploring. Another way to reduce the reaction of Ag and MHP is by developing auxiliary engineering technology, such as introducing a material layer to block ion migration, encapsulating the devices to prevent their exposure to O_2_ and moisture, and developing stable MHP materials. However, the formation and deformation of AgI seem to play a favorable role in MHP‐based devices such as memristors and artificial synapses.

The contact of MHP with the active Ag electrode is favorable for the memristor devices. The diffusion of silver atoms and silver halides into the MHP layer has attracted attention in recent years because it is closely related to the working mechanism of MHP‐based memristor devices. In memristors with the Ag electrode, Ag atoms diffuse into the MHP layer and react with MHP by substituting Pb ions due to the similar radius of Ag.^[^ ^239^ ^]^ Besides, it is believed that Ag ions are pulled to the electrode under the electric field, where an electrochemical reaction forms conductive filaments. The chemistry of the dissolved silver in MHP films will be reviewed in Section 5.3.

#### 5.1.3 Chemical Reaction at MHP/Al Contacts

**Figure 18:**
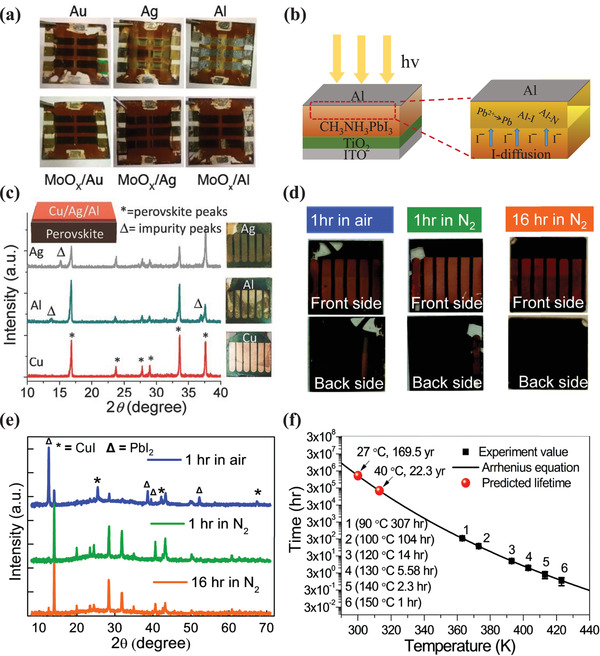
a) The decomposition for devices with and without the MoO*_x_* interlayer observed by optical images, indicating that device MoO*_x_*/Al contact are more stable than devices with Au and Ag electrodes. Reproduced with permission.^[^ ^247^ ^]^ Copyright 2016, American Chemical Society. b) Schematic illustration of Al electrode reaction with the MHP layer, the formation of the bonding between Al and N and aluminum iodide species, and the reduction of Pb^2+^ ions to metallic Pb species at the contact. c) Stability comparison of Ag, Al, and Cu deposited directly on MHP films. X‐ray diffraction (XRD) patterns of metal (Ag, Al, Cu)/MHP stacking layers after storing in air for 20 h. Top left inset shows the sample structure and right column shows photographs of those samples. Reproduced with permission.^[^ ^248^ ^]^ Copyright 2016, Wiley‐VCH. d) Photos taken from the front side and back side of the samples after being annealed at 100 °C in air and in N_2_ for 1 and 16 h. e) XRD pattern of the annealed samples. f) MHP stable time measured at different temperature. The black solid line shows the fitting of the experimental data with Arrhenius equation. Reproduced with permission.^[^ ^249^ ^]^ Copyright 2016, The Royal Society of Chemistry.

Al has a relatively low work function, which makes it possible as a top metal electrode in the MHP‐based PSCs with inverted structures and MHP‐based memristor, and the cost is much lower than Au or Ag electrodes. Al has been reported to face problems like air‐exposure‐induced oxidation and diffusion of Al atoms into the MHP layer. The reaction at the Al/MAPbI_3_ contact is different from Ag/MAPbI_3_ contact and Au/MAPbI_3_. As to Al/MAPbI_3_ contact, it is stable in static conditions. In situ synchrotron radiation photoemission spectroscopy (SRPES) and Pb 4f and I 3d XPS spectra on MHP/Al contact showed no obvious change in 24 h. It seems that the reaction between Al and I^−^ is not as easy as that between Ag and I^−^ at room temperature. This could explain the fact that MHP/MoO*_x_*/Al contacts are more stable than contacts of Au and Ag electrodes in ambient laboratory conditions (**Figure** **18**a).^[^ ^247^ ^]^ The introduction of ZnO in a device of ITO/PEDOT:PSS/MHP/ZnO/Al achieved better stability.^[^ ^250^ ^]^

Although it is believed that there is no substantial reaction between Al and I^−^, redox reactions between Al electrodes and perovskite films have been demonstrated to explain observed Al‐based device degradation phenomena. Using in situ SEM and X‐ray diffraction (XRD) measured under controlled humidity and vacuum pressure and ex situ XPS and EDX, Zhao et al. revealed a redox reaction between Al and MAPbI_3_ in which Pb^2+^ in the MAPbI_3_ film is reduced to Pb^0^ by Al. The comprehensive study of the chemistry of perovskite shows that MAPbI_3_ first converts to MA_4_PbI_6_·2H_2_O and then to MAI, during the redox. They argue that redox reactions are fundamental to the degradation of the devices of MAPbI_3_, CsPbI_3_, and CsPbBr_3_ perovskites with Al, Yb, or Cr contacts. Moreover, moisture plays a unique role in this process. It is argued that moisture enables continued reaction of the Al and perovskite layers by facilitating ion diffusion rather than serving as a decomposition reagent for the perovskite film.^[^ ^251^ ^]^ Ding et al. studied the interfacial structures and ion migration at the Al/MAPbI_3_ perovskite layer using SRPES. During the initial deposition of Al on the MAPbI_3_ surface, the in situ SRPES and XPS data revealed that the reaction of Al with the MHP layers led to the formation of aluminum iodide species, bonding between Al and N, and the reduction of Pb^2+^ ions to metallic Pb, as shown in Figure 18b.^[^ ^251^ ^]^ With the increase of Al deposition thickness, the diffusion of iodide species and aluminum can further promote the reaction, forming a narrow Pb^0^ distribution near the interface and exponential damping of Pb^0^ signal intensity at a depth below the surface. The diffusion depth of Al atoms is ≈10 nm during evaporation of the metal on substrates such as the PCBM layer and polymer layers. Comparing the two works, the corrosion of perovskite to the Al electrode, the need for water to participate in this process, and the role of water still need to be further clarified.

#### 5.1.4 Chemical Reactions at MHP/Cu Contacts

Cu electrodes may be one of the electrodes that can stably colocate with perovskite in the MHP‐based devices. The stability of the MHP‐based device is substantially enhanced by replacing Al or Ag with Cu. Unsealed devices with Cu electrodes can maintain performance in the air for 20–30 days, as shown in Figure 18c.^[^ ^248^ ^]^ It has been demonstrated that metal‐halide (CuI) was not detected after extended thermal stress.^[^ ^249^, ^252^ ^]^ However, for unencapsulated Cu‐based devices, Cu can be easily oxidized in the presence of moisture and oxygen via

(11) $$\mathrm{Cu}+\frac{1}{2}O_{2}+H_{2}O\to \mathrm{Cu}{\left(\mathrm{OH}\right)}_{2}$$

(12) $$\mathrm{Cu}+O_{2}\to {\mathrm{CuO}}_{X}$$

The surface oxidation of Cu causes an increased series resistance because of the poor conductivity of the oxide species. Introducing of a combined bilayer electrode by Ag and Cu is favorable to the device's stability. A thin film of Cu (10 nm) below the Ag (100 nm) electrode can effectively reduce the diffusion of species from the decomposed MHP layer into the electrode, indicating that Cu can reduce the corrosion of MHP decomposition products. In addition, the thermally evaporated Ag inhibits the oxidation of the Cu layer, showing resistance to water and oxygen.^[^ ^253^ ^]^ The encapsulation of the device can avoid exposing the intrinsic oxygen‐sensitive Cu to oxygen, which further reduces the possible reaction pathway between Cu and MHP (Figure 18d). The XRD data revealed no characteristic peaks of the CuI spectrum for ITO/PTAA/MAPbI_3_/Cu annealing at 100 °C in N_2_. This result confirmed the intrinsic stability of Cu/MAPbI_3_ contact in the absence of moisture and oxygen (Figure 18e). Based on these, Zhao et al. predicted that Cu‐based MHP devices could be stable for almost 170 years under room temperature and over 22 years under the nominal operating temperature (Figure 18f).^[^ ^249^ ^]^

However, the problem that Cu reacts with volatile species (MAI, HI, I^−^ ions) from the decomposition of MAPbI_3_ is still unclear. The auger electron spectroscopy measurement on the PSCs with a long time (100 h) of aging has shown that the Cu^+^ signals appear in the MHP layer, indicating that the Cu electrode is suffering some kinds of weak chemical reaction and corrosion. Moreover, Cu diffusion into MHP has been found in MHP‐based devices with metal Cu.^[^ ^254^ ^]^ Zhao et al. attributed the reaction at the Cu/MAPbI_3_ contact after one week of storage in the air to the reaction of Cu oxidation products (Cu(OH)_2_ or CuO*_x_*) with the products of perovskites decomposition.^[^ ^249^ ^]^ This explanation is consistent with the fact that CuI characteristic peaks are absent in the XRD spectrum measured in an MHP device with Cu in an N_2_ environment.

### 5.2 The Electronic Structures of MHP/Metal Contacts

Schottky's metal–semiconductor contact theory is possibly an ideal theoretical basis for MHP/metal contacts. According to Schottky's theory, when a metal and a semiconductor are put together, the band bending of the semiconductor occurs due to the alignment of Fermi levels. At the same time, there are potential barriers to charge transport. Taking a contact of an n‐type semiconductor with a metal electrode, for example, the potential barrier for electron diffusion from the semiconductor to metal is *ϕ*_m_ − *ϕ*_s_, while the potential barrier for hot‐electron injection from metal to semiconductor is *ϕ*_m_ − *E*_c_.^[^ ^171^ ^]^ Schottky's metal–semiconductor contact theory is occasionally used to explain qualitatively the electrical properties of devices with MHP/metal contacts. To explain the plasticity of MHP‐based synaptic conductance, a small contact barrier is helpful. Xiao et al. showed that with the stimulation of applied voltage, the ion distribution inside the MHP‐based synapse dynamically changes the potential barrier, resulting in memory characteristics of the current response to the device.^[^ ^21^ ^]^

#### 5.2.1 MHP/Metal Schottky Contact with Extended Depletion Region

**Figure 19:**
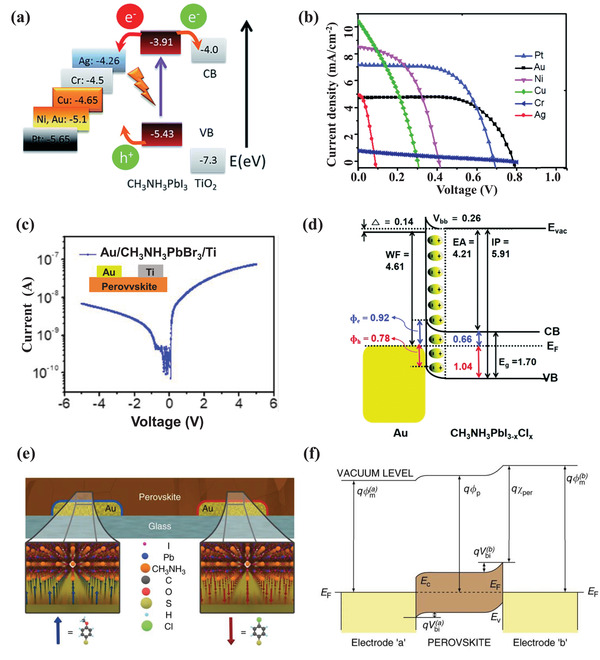
a) Schematic illustration of energy level alignments of MAPbI_3_ absorber against the Fermi level of different contact metals. b) *J*–*V* curves of HTL‐free PSCs with MAPbI_3_ contact with metals of different work functions. Reproduced with permission.^[^ ^82^ ^]^ Copyright 2016, The Royal Society of Chemistry. c) The *I*–*V* characteristics of Schottky diode device with a configuration of Au/perovskite (100 µm)/Ti. d) Schematic illustration of energy level alignment at the Au /MAPbI_3−_ *_x_* Cl*_x_* contact. Reproduced with permission.^[^ ^256^ ^]^ Copyright 2019, The Royal Society of Chemistry. e) Schematic illustration of cross‐section diagram of the back‐contact gold‐perovskite‐gold solar cell. The left Au anode is modified with a molecular monolayer of 4‐methoxythiophenol (OMeTP) with a molecular dipole of −2.67 D. The right Au cathode is modified with a monolayer of 4‐chlorothiophenol (ClTP) with a molecular dipole of +1.41 D. f) Schematic illustration of energy band diagram of a metal–MHP–metal solar cell at thermal equilibrium in the dark and cross‐section diagram of a self‐assembled dipole monolayer modified back‐contact gold–MHP–gold solar cell. Reproduced with permission.^[^ ^258^ ^]^ Copyright 2017, Springer Nature.

For undoped MHP semiconductors, the depletion region extends deep into the MHP film and is up to the order of micrometers. Thus, the depletion region is usually terminated by the electrode at the side of the MHP‐based devices with an MHP film thickness of several hundreds of nanometers. Consequently, the work function of the undoped MHP layer cannot influence the electrical performance of the MHP/metal contact devices. By contrast, the device's characteristics are largely determined by the electrode. It has been reported that metals with different work functions and various MHP/metal contact induce pronounced differences in the performance of HTL‐free PSCs.^[^ ^82^ ^]^ As shown in **Figure** **19**a, Cu, Ni, Cr, Pt, and Au have relatively high work functions of 4.65, 5.1, 4.5, 5.65, and 5.1 eV. It should be mentioned that Ag possesses a lower work function of 4.26 eV than the other metals. With these metals, Behrouznejad et al. studied the effect of different metal contacts on the HTL‐free PSCs with the configuration of Metal/MHP/ETL (TiO_2_)/FTO. In Figure 19b, the *V*_OC_ in HTL‐free PSCs depends on the work functions. As expected, Pt and Au contacts lead to larger values of *V*_OC_, while Ni, Cu, Cr, and Ag contacts result in lower values of *V*_OC_. By contrast, the effect of the metal contact is negligible for PSCs with Spiro‐OMeTAD. The results are consistent with the expectation of the metal–insulator–metal picture, which indicates that the built‐in voltage of the HTL‐free PSCs is determined by the difference in the work functions between TiO_2_ and the metal electrode.^[^ ^222^, ^223^ ^]^

The studies of MHP/metal Schottky contacts are crucial for applying MHP materials to perovskite electronics, including MHP‐based diodes, transistors, and memories. The single crystal of CH_3_NH_3_PbBr_3_/metal contacts has been studied, and the Schottky barriers are observed to be dependent on the work functions of the metal electrodes. For CH_3_NH_3_PbBr_3_ contact with Pt, Au, and Ti electrodes, the Schottky barriers are 0.38, 0.17, and 0.47 eV, respectively.^[^ ^255^ ^]^ Figure 19c shows a CH_3_NH_3_PbBr_3_ Schottky diode via Au and Ti contacts and its *I*–*V* characteristics. The diodes present a turn‐on voltage of 0.2 V, consistent with the Schottky barrier height of Au/CH_3_NH_3_PbBr_3_ contact (0.22 eV) considering the image charge effects. For the ideal Schottky barrier height of hole injection, according to Schottky theory, Schottky barrier height (*ϕ*_b_) between Au and CH_3_NH_3_PbBr_3_ is calculated as follows

(13) $$\phi_{b}=\mathrm{IP}-\phi_{M}$$

where IP is the ionization potential (5.6 eV) of CH_3_NH_3_PbBr_3_ and *φ*_M_ is the work function of Au (5.0 eV), which gives a Schottky barrier height of 0.6 eV. However, the surface pinning effect and the image charge at the MHP/Au junction interface were reported to reduce the height of the Schottky barrier to 0.20 eV.^[^ ^255^ ^]^

#### 5.2.2 Weak Fermi Level Pinning of the MHP/Au Contact

MHP/Au contact has a relatively stable electronic structure. In general, changing the energy level of the MHP/Au contact is easy. That is, the position of the energy level in the energy band is easy to shift. The Fermi level pinning effect is weak at the MHP/Au contact, and deep level defects (gap states) rarely form in the MHP materials due to their high activation energy. The energy level at the MHP/Au contact is sensitive to temperature, doping, dipole, image charge, and so on. The electronic structure for the interface of the MHP/Au usually exhibits an interface dipole and an upward band bending toward Au electrodes. Cha et al. investigated the electronic properties of the contact between Au and MAPbI_3‐_ *_x_* Cl*_x_* via ultraviolet photoelectron spectroscopy and XPS (Figure 19d). They found an energy level shifted by 0.26 eV upward the Au electrode and attributed the energy levels shift to an interface dipole. This exists at the Au/MAPbI_3‐_ *_x_* Cl*_x_* contact.^[^ ^256^ ^]^ The results are consistent with the work of Liu et al., who studied the electronic properties of MAPbI_3_/Au contact.^[^ ^257^ ^]^ Liu et al. proved that the electronic structure at the interface contains two parts: one interface dipole of 0.1 eV and one shift of energy levels of MAPbI_3_ upward by 0.4 eV. This finding indicated a built‐in electric field in the MAPbI_3_ layer, which promoted hole transfer from MAPbI_3_ to Au.

Because of the weak Fermi level pinning, the work function of Au could be adjusted by self‐assembled monolayers containing oriented molecules with certain dipole moments. Lin et al. modified two sets of interdigital gold microelectrode arrays, termed “a” and “b”, with a molecular 4‐methoxythiophenol (OMeTP) for set “a” and a molecular monolayer of 4‐chlorothiophenol (ClTP) for set “b”, as shown in Figure 19e, producing two sets of microelectrode arrays with different work functions. They further fabricated a gold–MHP–gold back‐contact PSC with the two sets of microelectrode arrays. The Kelvin probe force microscopy reveals that the device presents different work functions between “a” and “b” microelectrodes, and the difference is up to 600 mV.^[^ ^258^ ^]^ As a result, a pair of asymmetric built‐in potentials at the front and back contact is produced, as shown in Figure 19f. Ultimately, the total built‐in potential across the solar cell equals the sum of the built‐in potential at the MHP/metal contacts.

### 5.3 Electrochemical Metallization and Conductive Metal Filament

The origin of resistive switching of MHP‐based RRAM has aroused interest. The metal electrode plays a crucial role in the resistive switching effects of MHP‐based RRAM devices. When changing the top electrode from Au to Ag or Al, there is a difference in *I*–*V* characteristics. The electrochemical process of metal ions in the MHP layer determines the resistive switching behaviors. Moreover, electrochemical metallization and conductive metal filament formation models in MHP‐based RRAM have been developed.

#### 5.3.1 Resistive Switching Characteristics

**Figure 20:**
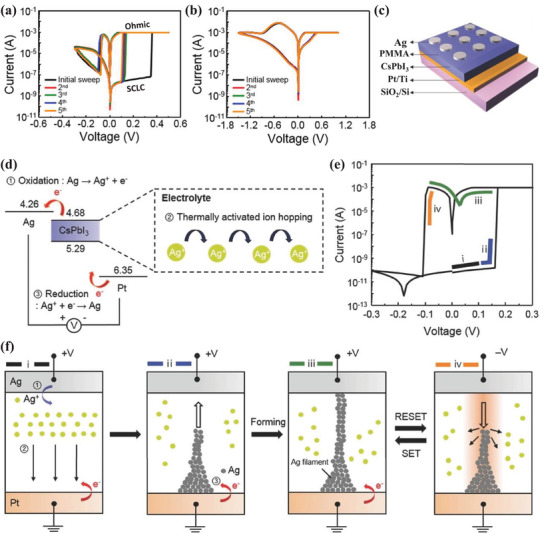
a) A typical sharp change resistive switching *I*–*V* curve in a Ag/PMMA/CsSnI_3_/Pt device. b) A gradual change resistive switching *I*–*V* curve in a Au/PMMA/CsSnI_3_/Pt device. Reproduced with permission.^[^ ^129^ ^]^ Copyright 2019, American Chemical Society. c) Schematic drawing of a Ag/PMMA/CsPbI_3_/Pt vertical stack structure for resistive switching. d) Schematic illustration of the Ag/PMMA/CsPbI_3_/Pt junction band diagram and the thermally activated ion hopping in the MHP layer. e) Typical *I*–*V* characteristics of the Ag/PMMA/CsPbI_3_/Pt device. f) Schematic illustration of the formation and rupture of the Ag conducting filament. Reproduced with permission.^[^ ^126^ ^]^ Copyright 2017, Wiley‐VCH.

**Figure** **20**a,b shows two typical resistive switching *I*–*V* characteristics in MHP‐based RRAM devices. During the resistive switching behavior measurement, the bottom electrode was on the ground, and a bias voltage was applied to the top electrode. The voltage sweeps following a sequence of 0 V → +*V*_max_ → 0 V→ −*V*_max_ → 0 V with a specific scanning frequency and step size.^[^ ^129^ ^]^ It can be observed that both of the typical *I*–*V* characteristics exhibit two conduction states, called the high resistance state (HRS) and the low resistance state (LRS), corresponding to ON (after writing) and OFF (before writing) states in the memory device. The MHP‐based RRAM exhibits bipolar switching behavior.

The ON/OFF switching or the LRS/HRS switching is achieved by SET and RESET processes. Figure 20a exhibits irreversible current breakdown or sharp change in the SET and RESET processes, while Figure 20b shows a negative differential resistance region and a gradual current change region when the device enters the HRS regime from the LRS regime. In most cases, the sharp‐changing resistive switching *I*–*V* is present in the devices employing electrochemically active metals, such as Ag and Al.^[^ ^19^, ^239^ ^]^ Han et al. have fabricated a vertical stack device composed of Ag/poly(methyl methacrylate) (PMMA)/CsPbI_3_/Pt/Ti/SiO_2_/Si. Initially, the device was in the HRS with a low current (10^−11^–10^−10^ A). When the positive sweeping voltage surpassed the operating voltage of 0.18 V, the device was sharply switched to a high current (10^−3^ A) and maintained the LRS during the voltage sweeping +0.5 V → 0 V → −0.3 V until the reset voltage of −0.1 V reached. Then the device was sharply switched to the HRS with a low current of 10^−12^–10^−11^ A.^[^ ^129^ ^]^ Similar sharp current changes have also been observed in RRAM devices with Al electrodes.^[^ ^239^ ^]^ The results indicate that active metal is necessary for the sharp current changes in resistive switching behaviors. By contrast, the MHP memory with chemically stable electrodes, such as Au and FTO, shows a gradual‐change resistive switching *I*–*V*.^[^ ^129^, ^152^ ^]^ Thus, the difference in *I*–*V* characteristics demonstrates the existence of different kinds of resistive switching mechanisms.

#### 5.3.2 Electrochemical Metallization

The electrochemical metallization mechanism is recently used to understand the sharply resistive switching behaviors. The mechanism depends on an electrochemically active electrode metal such as Ag, the metal ion conductive layer, and their metallization through discharge at the counter electrode.^[^ ^154^ ^]^ Basically, the metallization gathers metal ions and forms a highly conductive filament, which induces the LRS (the ON state of the RRAM). When the polarity of the applied voltage is reversed, metal filaments are electrochemically dissolved, resetting the system into the HRS (the OFF state of the RRAM).

MHP‐based resistive switching memory devices with the Ag or Al electrode have been reported to exhibit filamentary behavior by the metallic conducting filament. Based on the electrochemical metallization, the metallic conducting filament happens by the dissolution of the active metal. Then, metal cations migrate across the MHP thin film and are electrochemically metalized near the back electrode. For devices with Ag electrodes, the active metal Ag becomes Ag^+^ under a positive electric field. Then, the generated Ag^+^ migrates to the back electrode under the positive electric field. When Ag^+^ reaches the back electrode, the Ag conductive filament is formed by the reduction of Ag^+^ to metal Ag. Many groups adopted the formation and rupture of the metal resistive switching effect to explain the resistive switching of the sharp SET and RESET types.^[^ ^128^, ^129^, ^239^ ^]^ For example, Figure 20c schematically illustrates Ag/PMMA/CsPbI_3_/Pt vertical stack structure for resistive switching. Figure 20d shows a proposed mechanism for the resistive switching behavior of the device with the configuration of Ag/PMMA/CsPbI_3_/Pt, containing the oxidation of the Ag electrode, the thermal assistant Ag ion hoping, and the reduction of Ag^+^ into Ag at the Pt back electrode. Figure 20e shows the typical *I*–*V* characteristics of the Ag/PMMA/CsPbI_3_/Pt device, presenting an OFF state (i), an abrupt SET process (ii), a high ON state (iii), and an abrupt RESET process (iv). Figure 20f illustrates the state of the Ag filament corresponding to the typical *I*–*V* characteristics. Initially, oxidation under the applied voltage dissolved part of the Ag electrode. Then, the generated Ag^+^ migrates to the Pt electrode under the applied electric field. Here, the device is in the OFF state of low conductivity. When Ag^+^ arrives at the Pt electrode, it is reduced to metal Ag, forming a metal filament between the Pt electrode and the Ag electrode. The SET process occurs when the device changes sharply from the OFF state to the ON state. Then, the filament disruption occurs under an applied electric field in the opposite direction, resulting in the RESET process.

Based on the insight, the SET voltages are determined by the migration of the Ag^+^ and the supersaturation of the metal close to the counter electrode. Han and co‐workers showed an inhomogeneous nucleation theory by applying the classical nucleation theory for the supersaturation of the metal. According to the theory, *J* is the nucleation rate of Ag, which is described by the following equation

(14) $$J=J_{0}\exp\left(\frac{-\Delta G^{*}}{k_{B}T}\right)$$

(15) $$J_{0}=\rho_{\mathrm{Ag}}\left(\frac{k_{B}T}{h}\right)\exp\left(\frac{-\Delta g^{*}}{k_{B}T}\right)$$

where ΔG* is the maximum value of free energy change, *ρ*_Ag_ is the density of Ag in the liquid state, Δ*g* is the activation energy of Ag atom when it transports across an interface of the nucleus, and *h* is the Plank constant. The nucleation mechanism reveals that a low voltage is required for supersaturation, which leads to low SET voltages at high temperatures. On the contrary, low temperatures will give rise to high SET voltages.^[^ ^126^ ^]^

Generally, a thermally activated ion hopping mechanism is used to describe the migration of metal cations between adjacent sites in the MHP layer.^[^ ^126^ ^]^ At high temperatures, the migration speed of the Ag^+^ (*v_i_*) has been described by

(16) $$v_{i}=\frac{Aa^{2}q_{i}E}{2k_{B}T}\exp\left(\frac{-\Delta H}{k_{B}T}\right)$$

where Δ*H* is the energy barrier between adjacent sites, *a* is the distance of the adjacent sites, *E* is the applied electric field, and *q_i_* is the ionic charge. The equation shows a linear relationship between *v_i_* and *E*. It is believed that Ag^+^ has a low energy barrier between adjacent sites. Thus Ag can easily reach supersaturation at a low bias voltage, forming a small SET voltage that is usually observed in the MHP‐based RRAM with Ag electrode. The theory is consistent with the observation of the SET voltage in Ag/PMMA/CsPbI_3_/Pt device measured at varying temperatures of 253, 273, 293, 313, and 333 K, which showed a decreasing tendency with increasing temperature.^[^ ^126^ ^]^

The typical sharp change of resistive switching *I*–*V* curve has been reported in Al‐based devices, such as the ITO/PEI/MAPbI_3_/PEI/Al reported by Wu and co‐workers. They proved that the excellent penetration ability of Al atoms during thermal evaporation could penetrate the MHP layer. These metal atoms were then ionized into Al ions (Al^3+^) upon the charge injection. They assumed that the Al^3+^ was moved along the grain boundaries. As a result, Al metallization happened with formed filaments extending from Al to ITO electrodes. The device composed of ITO/PEI/MAPbI_3_/PEI/Ag showed a gradual changed switching *I*–*V* curve, but not sharply changing switching *I*–*V* curve. Ag^+^ did not participate in conductive filament formation, but it was consumed in substitution of Pb^2+^ in the perovskite crystal structure due to the similar ionic radius (115 pm) with Pb^2+^ (119 pm). By contrast, the ionic radius of Al^3+^ is 53.5 pm Therefore, Al^3+^ cannot substitute Pb^2+^ in the MHP crystal structure.^[^ ^239^ ^]^ For the active Ag electrode device, the absence of sharp change may be due to the effect of the PEI layer. The corrosion of MHP on the electrode is reduced, resulting in a decreased concentration of Ag^+^ in the MHP layer. The supersaturation of the metal Ag cannot be achieved in this case.

#### 5.3.3 Electrochemical Metallization and Vacancy Migration

According to the conductive channels associated with electrodes, the underlying mechanisms of the resistive switching are divided into interface‐type and filamentary‐type, corresponding to the gradual‐change resistive switching *I*–*V* curves and the sharp‐change resistive switching *I*–*V* curve, respectively.^[^ ^19^ ^]^ Han et al. analyzed the conduction mechanism of the Au/PMMA/CsSnI_3_/Pt device from the *I*–*V* characteristics of the HRS and LRS, which shows a variation from the Schottky emission HRS region to the linear ohmic conduction LRS region. The resistive switching behavior can be attributed to the Sn vacancies with the lowest formation energy among the defects of CsSnI_3_. Specifically, the migration of Sn vacancies under the applied voltage will induce interface‐type switching at the MHP/metal contacts. Under the positive bias, Sn vacancies will accumulate at the interface, and the depletion width in the MHP layer will decrease, which will turn the device into the LRS. On the contrary, decreasing in the number of vacancies at the interface will lead to an increased depletion width at the MHP/metal contact and the HRS.^[^ ^129^ ^]^ However, Yoo et al. proposed that there was charge carrier trapping and migration of iodide vacancies at the MAPbCl*_x_* I_3‐_ *_x_*/Au contact in the devices of Au/MAPbCl*_x_* I_3‐_ *_x_*/FTP.^[^ ^121^ ^]^ Therefore, it still needs to be further studied whether the trap effect or the Schottky barrier effect is at the MHP/metal contact causing gradual change resistive switching *I*–*V*.

### 5.4 Interface Engineering of MHP/Metal Contact

Interface engineering has been studied for decades of organic devices, corrosion science, silicon semiconductor devices, and interfaces between MHP and transport layers. However, there have been rare reports of the engineering in MHP/metal contacts within the field of MHP‐based devices in recent years.

#### 5.4.1 Restraint of Electrode Corrosion and MHP Decomposition

**Figure 21:**
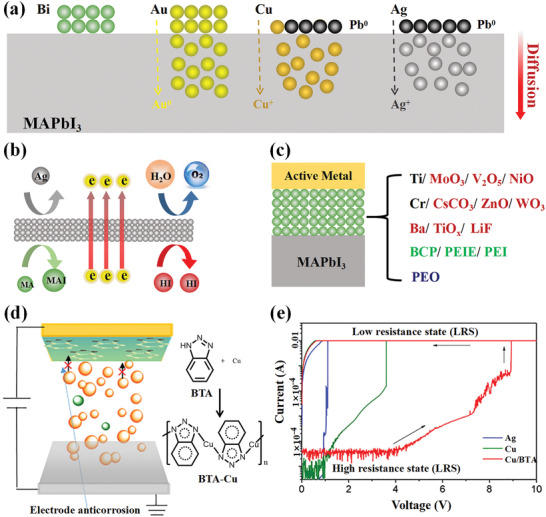
a) Schematic illustration of metal diffusion into the MHP and chemical reaction of MHP/metal contact. b) Schematic diagram of the functions of the introduced insertion layer between MHP and metal electrode. c) Interfacial engineering of the MHP/metal contact employing metal oxides, such as MoO_3_, CsCO_3_, TiO*_x_*, NiO, WO_3_, V_2_O_5_, and ZnO, LiF, bathocuproine (BCP), and organic or polymer insulator layers. d) Schematic illustration of MHP‐based RRAM devices with Cu electrode, to which the BTA molecules chemically coordinate, the reaction with the top electrode of MHP is suppressed because of the existence of BTA. e) *I*–*V* curves in RRAM devices with different metal electrodes. RRAM with BTA/Cu exhibiting ≈2.5 times higher transiting bias (8.9 V than that with Cu of 3.6 V). Reproduced with permission.^[^ ^254^ ^]^ Copyright Year, American Association for the Advancement of Science.

One of the primary goals of MHP/metal interface engineering is to achieve the continuous coexistence of active metals and chemically stable MHP layers at the contacts. The metal ions are usually observed in the MHP layer due to the reaction between the metal electrodes and MHP. Wu et al. studied the corrosion reactions of the metal when MHP is directly in contact with metals, such as Au, Cu, Ag, and Bi, and revealed the reaction mechanism and the diffusion process of metal ions and atoms into the MHP layer via XPS, as shown in **Figure** **21**a.^[^ ^259^ ^]^ Even worse, the thermal aging of the device will aggravate the metal ions entering the film layer. Domanski et al. proved that a mass of Au diffused inside the MHP layer and accumulated near the TiO_2_/SnO_2_ interface when the device was aged at 70 °C by the time of flight secondary ion mass spectroscopy elemental depth profiling and inductively coupled plasma mass spectrometry test of the device TiO_2_/mesoporous TiO_2_/MHP/Spiro‐MeOTAD/Au.^[^ ^260^ ^]^

Using chemically stable metal as an interlayer is the direct interface engineering method. Inserting chemically stable metals or low diffusivity metals, such as Bi, Cr Ba, and Ti, between the active metal electrode and MHP film, has been shown to prevent electrode corrosion and MHP decomposition by insulating the MHP from undesirable external moisture and protecting the metal electrode from iodine corrosion, as shown in Figure 21b. The chemically stable metal of Bi can act as a robust permeation barrier with a low diffusivity, which presents certain anticorrosivity to MHP, even during thermal aging of the MHP/metal contacts.^[^ ^259^ ^]^ A thin interlayer of Cr (10 nm) can be used as a diffusion obstacle between the Spiro‐MeOTAD and Au, resulting in unprecedented high‐temperature stability of Cr/Au devices that have never been observed in devices using Au or Ag as contacts.^[^ ^260^ ^]^ Chen et al. innovatively inserted an ultrathin layer of Ti between MHP film and metal electrode in ETL‐free PSCs (see Figure 21c). The Ti interlayer can prevent the diffusion of metal atoms into the MHP layer, passivate surface defects and suppress surface decomposition of MHP film, leading to improved device performance and stability. Similarly, Gupta et al. found that a Ba layer with a thickness of 10–20 nm between Al and semiconductors could significantly improve the device performances because of an increased built‐in potential after inserting the Ba layer with the lower work function of 2.7 eV compared to the work function of Al (4.3 eV).^[^ ^261^ ^]^

Interface engineering for MHP‐based memory devices with thin layer polymer insulators can prevent the exposure of the MHP film to oxygen and moisture under ambient conditions and the reaction of the MHP film with the top metal electrode. Several memory devices have inserted a thin PMMA layer with a few nanometers between the metal electrode and the MHP film. As a result, high‐endurable RRAM devices were fabricated with increased endurance cycles, stability, and reproducibility of the resistive switching behavior.^[^ ^126^, ^152^, ^262^ ^]^ Recently, Han and co‐workers demonstrated that inserting PEI interfacial layers into both the MHP/top‐metal contact and MHP/bottom‐metal contact enabled the device of ITO/PEI/MAPbI_3_/PEI/metal to achieve endurance cycles of more than 4000 times while keeping a low operation voltage around 0.25 V. In addition, resistive switching behavior was reproduced among 180 memory devices showing good reproducibility. They excluded the possible memory effect caused by replacing of the MHP layer in the ITO/PEI/PCBM(PTAA or P3HT)/PEI/Ag, which exhibited no resistive switching behavior. A chemical anticorrosion strategy was introduced by a specific organic corrosion inhibitor of benzotriazole (BTA) between the Cu electrode and MHP. As shown in Figure 21d, the BTA molecule chemically coordinated to metal Cu, and an insoluble polymer membrane of [BTA‐Cu] formed, which could suppress the electrochemical corrosion and reaction between MHP and the Cu electrode. It was proved that PSCs with BTA/Cu achieved superior air stability, retaining 92.8 ± 1.9% of initial efficiency after aging for 2500 h and >90% of initial efficiency retained after 85 °C aging for over 1000 h.^[^ ^254^ ^]^ In addition, RRAM with BTA/Cu exhibited ≈2.5 times higher transiting bias (8.9 V) (see Figure 21e) than that with Cu (3.6 V), which also indicated that BTA molecules could effectively suppress the Cu electrode reaction and corrosion.

In addition to inserting interlayers between the metal and MHP layer, replacing the easily corroded metal electrode with a carbon electrode is meaningful for the large‐scale production of ETL‐free PSCs and MHP‐based devices. Graphite/amorphous carbon, graphene, and CNTs can also be used as alternatives for back electrodes because of their low cost, high conductivity, and low‐temperature processing techniques.^[^ ^263^, ^264^, ^265^, ^266^ ^]^ Carbon electrodes can overcome another drawback of noble metal electrodes, i.e., the deposition of the expensive metals requires an energy‐consuming vacuum evaporation method. Besides, the carbon electrodes are used to improve the device stability due to their hydrophobic property that can promote the carbon electrode to protect the MHP layer from moisture.^[^ ^266^ ^]^ MHP/carbon modification should be developed to achieve high‐performance carbon‐based PSCs. However, MHP/carbon usually suffers from the problem of energy level mismatch, where the work function of carbon is much lower than that of MHP. Wu et al. achieved the energy level alignment between FA‐Cs‐based MHP/carbon electrodes by modifying the contact with a thin layer of poly(ethylene oxide) (PEO) that was spin‐coated on the top of MHP films. The work function of MHP/PEO decreased from 4.08 to 3.52 eV, attributed to the formation of surface dipole after introducing the PEO layer. Ultimately, the reduction of energy level mismatch and formation of Schottky barrier formation with spontaneous upward band bending is expected, which can promote the extraction of holes and effectively restrain electron transfer from MHP to carbon, resulting in a PCE improvement from 12.2% to 14.9%.^[^ ^266^ ^]^

#### 5.4.2 Optimization of the Contact Electronic Structure

Another aspect of MHP/metal interface engineering is optimizing the electronic structures, such as the work functions of the metal. To achieve efficient MHP‐based devices, tremendous efforts have been devoted to the interfacial engineering of the MHP/metal contact employing metal oxides, such as MoO_3_, TiO*_x_*, NiO, CsCO_3_, V_2_O_5_, WO_3_, and ZnO, LiF, BCP, and organic insulator layers.^[^ ^230^, ^268^ ^]^ Interlayers can significantly improve the fill factor and facilitate the efficient extraction of holes or electrons by minimizing the contact resistance and charge recombination at the contacts. The employment of interlayers can circumvent the direct contact between the active electrodes and photoactive donor (polymer or small molecule) that causes high densities of carrier traps and interface dipoles, hindering efficient charge collection.^[^ ^261^ ^]^ The introduction of metallic oxide nanoparticles in PSCs, for example, p‐type NiO*_x_* and n‐type ZnO as HTL and ETL, respectively, have been shown to improve device stability against degradation caused by water and oxygen.^[^ ^250^ ^]^ MoO_3_ oxides are usually coated onto electrodes to meet the energy‐level alignment at the interface.^[^ ^268^ ^]^ Greiner et al. showed a detailed study on the metal/MoO_3_ contact and attributed the shift of work function to two factors: One is the charge transfer. Electrons around the Fermi level transport from the metal into the MoO_3_ layer and occupy the low‐lying conduction band. The other is the oxidation–reduction reaction; the contact of metal and MoO_3_ results in metal oxidation and MoO_3_ reduction. Moreover, the metal oxide layer could prevent degradation of the metal and MHP by isolating the MHP layer and Al electrode.^[^ ^268^, ^269^ ^]^ Zhou et al. modified the work function of the metal by self‐assembled monolayers of dipolar polymers, including aliphatic amine groups, for instance, branched PEI and PEIE.^[^ ^230^ ^]^ Lee et al. utilized PEIE to modify Ag and Cu electrodes in the devices of ITO/HTL/MAPbI_3_/PCBM/Ag or Cu, decreasing the metal work functions from the original 4.6 or 4.7 to 3.9 or 4.0 eV, respectively.^[^ ^270^ ^]^ Lin et al. have modified the Au substrates by self‐assembled monolayers of two parasubstituted thiolbenzene derivatives with permanent molecular dipoles. By introducing dipole moment in the opposite direction, Au substrates can achieve persistent work function difference of up to 600 mV.^[^ ^258^ ^]^

#### 5.4.3 Promising 2D/3D MHP Interface Engineering

In the field of MHP PSCs, 2D perovskite fabricated on the bulk 3D perovskite has recently attracted much attention. The 2D/3D MHP junction is usually in contact with transporting layers in standard full‐structure PSCs.^[^ ^271^ ^]^ It is widely reported that 2D/3D perovskite interfaces work as a standard route to enhance the efficiency and stability of the PSCs. The underlying mechanism of the effective strategy is the efficient engineering of the MHP/TL interface simply by constructing a thin layer of 2D perovskite on the 3D MHP substrate. It is worth noting that 2D/3D MHP junction works well when in contact with various transporting layers, such as PTAA, Spiro‐OMeTAD, CuSCN, carbon matrix, etc.^[^ ^272^ ^]^ This implies that 2D perovskite mainly produces the marked effect by changing the properties of the interface on the MHP side. Further studies show that the 2D/3D interaction widens the band gap in the interface region. As a result, the 2D layer can optimize the energetic alignment and reduce electron density, which has significantly suppressed the nonradiative interfacial recombination losses by the QFLS method.^[^ ^273^ ^]^ The alignment of the valence‐/conduction‐band edge has been argued to have synergistic advantages: the graded combination enhances the hole extraction and conduction efficiency with effectively decreased recombination loss during the hole‐transfer process, leading to an enhanced built‐in electric field, hence a high *V*_OC_ of as much as 1.19 V.^[^ ^274^ ^]^ Moreover, the 2D crystalline structure keeps the 3D bulk underneath intact, which blocks the 3D bulk degradation into lead iodide even under thermal stress, revealing the paramount role of 2D perovskite in engineering stable device interfaces.^[^ ^275^ ^]^ It also reported that the 2D/3D graded interface structure suppresses ion migration, which contributes to the device's thermal stability.^[^ ^276^ ^]^

The behavior of 2D perovskite materials with electrodes offers promising developments in optoelectronic. 2D/3D MHP junction could bring beneficial interface engineering by altering the MHP properties at the MHP/metal contact, as it has done at the MHP/TL contacts. However, only a few works have been reported, showing that 2D/3D MHP junction functions well in TL‐free devices. Graded band structure can form in the 2D‐3D MHP heterojunction in the HTL‐free PSCs, using 2D perovskite as hole‐transport material to overcome the high cost and instability issues caused by the traditional organic HTMs. The efficient and stable device presents a 2D MHP/Au contact, indicating that the 2D/3D MHP junction is also stable with contact with gold.^[^ ^277^ ^]^ However, it is still unclear whether 2D/3D MHP junctions in contact with other metal electrodes show the same stability in contact with transporting layers. There are few reports on TL‐free devices of 2D/3D MHP contact directly with TCO substrates. It is still a technological challenge to insert a layer of 2D MHP between ITO and the bulk 3D MHP. We look forward to the breakthrough in the preparation of 2D/3D MHP/metal contacts, and the resulting considerable improvement of the TL‐free devices.

## 6 Modeling Electronic Devices with MHP/Electrode Contacts

Physical modeling aims to reconstruct the detailed device physics and realize the high reproducibility of the macroscopic electrical performance. For example, a self‐consistent optoelectronic simulation (the simulation of solar cells) contains the solution of the Maxwell equation, Poisson equation, drift‐diffusion equations, and ion migrations equations. A complete physical modeling process for one type of device involves the construction of device physics, research on the numerical method, and device analysis applications. The physical modeling for silicon‐based devices is well‐developed, which can provide a systematic simulation study and detailed power‐loss analysis on silicon solar cells.^[^ ^278^ ^]^ The experimental research and physical modeling of devices are mutually promoted. Here, we present recent progress in the physical modeling of the ETL/HTL‐free PSCs and the MHP‐based memristors.

### 6.1 Device Physics Modeling of ETL/HTL‐Free PSCs

The physical modeling of photovoltaic devices is based on drift‐diffusion equations and the Poisson equation. It helps predict the electrical characteristics of devices, such as the *J*–*V* curve, spectral responsivity, and power conversion efficiency. It also works as a consistent platform for studying the effect of the physical processes at the interface as well as in bulk on device performances. Here, we summarize the primary device physics simulation conclusions on the perovskite solar cells. Then, we discuss as a feature the treatment and conclusion of the MHP/electrode contact in the device's physical simulation.

The physical modeling of the ETL/HTL‐free PSCs is based on the simulation of the full‐structure PSCs devices, which have revealed the influences of the charge carrier process and MHP materials properties on the device performance. For example, the physical modeling revealed that the fast exciton dissociation of the MHP due to small exciton binding energy might improve the short‐circuit current and the fill factor, and the reduced bimolecular recombination may have a recombination coefficient that is several orders of magnitudes smaller than that based on the Langevin's theory, which leads to a high quasi‐Fermi level splitting and thus a small *V*_OC_ loss.^[^ ^279^ ^]^ Olyaeefar et al. have incorporated the permittivity, the effective density of states, charge carrier mobility, and doping density in the physical modeling and revealed that the interface defects were the major factor in the PSCs with the bulk defect density around 10^16^ cm^−3^ and surface recombination velocities in the range of 10 cm s^−1^.^[^ ^280^ ^]^ Through fitting the experimental results of several MHP‐based PSCs, the device model revealed that the dominant recombination limiting the performance of the device is trap‐assisted recombination at material interfaces, and a PCE of beyond 25% can be reached by passivation of traps.^[^ ^281^, ^282^ ^]^

**Figure 22:**
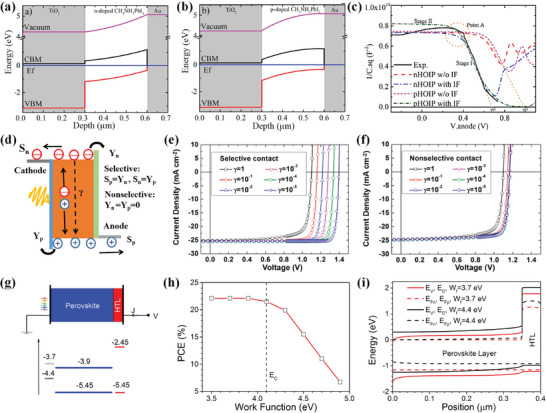
a) Static band diagram of n‐doped HTM‐free PSCs with a configuration of TiO_2_/MAPbI_3_/Au. b) Static band diagram of p‐doped HTM‐free PSCs with a configuration of TiO_2_/MAPbI_3_/Au. c) The computed and experimental (solid line) capacitance–voltage (*C*–*V*) characteristics in dark of HTL‐free PSCs. N‐doped MHP with/without an n‐doped interfacial layer (IF) at Au/MHP interface is indicated as dash dot (dash) line. P‐doped MHP with/without a p‐doped IF at TiO_2_/MHP interface is indicated as short dash dot (short dash) line. Reproduced under the terms of a Creative Commons Attribution 4.0 International License.^[^ ^267^ ^]^ Copyright 2017, The Authors. Published by EDP Sciences. d) Schematics illustration of the architecture and charge carrier transfer at the contact of PSCs under simulated. e) The calculated current *J*–*V* characteristics of PSCs with selective contact (full‐structure) under different radiative recombination by changing the reduction factor of *γ*. f) The calculated current *J*–*V* characteristics of PSCs with nonselective contact (ETL/HTL‐free) under different radiative recombination by changing the reduction factor of *γ*. Reproduced with permission.^[^ ^201^ ^]^ Copyright 2017, American Chemical Society. g) Schematics illustration of the architecture and band diagram of the simulated ETL‐free PSCs with different cathode work functions of 4.4 and 3.7 eV. h) The simulated dependence of the *V*_OC_ and fill factor on the work function of the cathode. i) The simulated band diagrams of the ETL‐free PSCs with the electrode work functions of 4.4 and 3.7 eV respectively under the open‐circuit condition. Reproduced with permission.^[^ ^26^ ^]^ Copyright 2020, American Chemical Society.

In most physical modeling, the origin of photovoltage of the ETL/HTL‐free PSCs is in line with the metal–insulator–metal mechanism. The energy band diagrams from simulations usually exhibit a completely depleted MHP and a uniform built‐in electric field. For example, Behrouznejad et al. calculated the band bending diagrams by SCAPS software for HTL‐free PSCs with metal electrodes of different work functions. Their simulation demonstrated that the built‐in potential depends on the work function of the metal, which is similar to the metal–insulator–metal picture. In the condition of metals with high work functions, an enlarged built‐in potential is present, producing a considerable *V*_OC_ to the HTL‐free devices, which has been confirmed in the study of the PSCs with Cu or Pt electrodes.^[^ ^82^ ^]^ However, the MHP/metal contact simulations are implemented under ideal conditions with no electrode corrosion, perovskite doping, interface dipole, and so on. Therefore, to improve the device's reproducibility, the MHP/metal contact modeling must be constructed in device physics. The collaborative research of simulation and experiments can address this issue. Huang et al. gave a detailed simulation of the band bending and the capacitance–voltage characteristics of PSCs with and without an HTL. **Figure** **22**a,b illustrates the static band diagrams of n‐doped HTL‐free PSCs and p‐doped HTL‐free PSCs with a configuration of TiO_2_/MAPbI_3_/Au, respectively. Figure 22c shows the *C*–*V* characteristics in the dark of HTL‐free PSCs. N‐doped MHP with an n‐doped interfacial layer at Au/MHP interface is indicated as the dash‐dot (dash) line. P‐doped MHP with a p‐doped interfacial layer at TiO_2_/MHP interface is indicated as the short dash‐dot (short dash) line. From the simulation results and experimental data, it can be seen that the n‐doped interfacial layer between Au and MAPbI_3_ with a thickness of 8.5 nm should be introduced into the architecture with MAPbI_3_ to fit the experimental data. The result indicates that the MHP semiconductor's properties have changed because of the contact with the metal.^[^ ^267^ ^]^ One obvious result was that the doping layer gave rise to the inhomogeneity of band bending, and the band bent more markedly at one contact and less at the other. It is not difficult to understand that the doping adjusts the band bending in the MHP region and deviates from the metal–insulator–metal picture by introducing charges into the doped region. The experimental results showed that the *V*_OC_ of ETL/HTL‐free PSCs is much lower than that of being predicted by the metal–insulator–metal picture, especially for devices with MHP/metal contact. From the perspective of device simulation, the nonuniformity of microstructures at MHP/metal contacts caused by adsorption effect, corrosion, and interface morphology is still unclear.

Another important mission of the physics modeling in ETL/HTL‐free PSCs devices is to understand the limitation set by specific internal factors to optimize device performance. Based on the device simulation, Ren et al. showed that giant recombination losses were present in PSCs with nonselective contacts due to the recombination of the holes with thermally activated electrons at the cathode. Figure 22d illustrates the architecture and charge carrier transfer at the contact of PSCs under the simulation. Figure 22e shows the calculated current *J*–*V* characteristics of PSCs with selective contacts under different radiative recombination by varying the reduction factor *γ*. Figure 22f shows the calculated current *J*–*V* characteristics of PSCs with ETL/HTL‐free contact realized by changing the reduction factor of *γ* during the simulation. The contacts set a limit to the performance of PSCs with a smaller *V*_OC_ of 1.17 V together with a lower fill factor of 82.5%, a short‐circuit current density of 24.74 mA cm^−2^, and a PCE of 23.83%, compared to that of the selective contacts PSCs with *V*_OC_ of 1.30 V together with a fill factor of 90.39%, a short‐circuit current density of 25.38 mA cm^−2^, and a PCE of 29.86%.^[^ ^201^ ^]^ The work indicated that ETL/HTL‐free PSCs (PSCs with nonselective contacts) could achieve comparable performance as the full‐structure device by suppressing the interface recombination. Lin et al. performed the device modeling of HTL‐free PSCs with a configuration of FTO/ZnO/MAPbI_3_/carbon, and their simulation revealed that both the Fermi level and doping concentrations in the MAPbI_3_ layer significantly affected the performance of the device. By controlling the trap densities at the MAPbI_3_/ZnO interface under the order of magnitudes ≈10^17^ cm^3^ and the doping concentration of MHP to 10^16^ cm^3^, a PCE of 18.11% could be obtained.^[^ ^286^ ^]^

In addition to interface recombination, the device model simulation on the ETL/HTL‐free PSCs revealed that the work function of the MHP/electrode contact significantly influences the device performance. Wang et al. simulated the *J*–*V* characteristics of the full‐structure device of ITO/TiO_2_/MAPbI_3_/Spiro‐OMeTAD/Au and the corresponding HTL‐free device, respectively. The PCE of the HTL‐free device with 11.35% is much lower than that of the full‐structure device of 19.02%.^[^ ^287^ ^]^ Li et al. performed an SCAPS simulation on the typical planar ETL‐free PSCs with a configuration of ITO/MAPbI_3_/Spiro‐MeOTAD/Au to further illustrate the effect of the reduced working function on band bending and device performance. Figure 22g illustrates the architecture and band diagram of the simulated ETL‐free PSCs with the different cathode work functions of 4.4 and 3.7 eV. Figure 22h shows the simulated dependence of the *V*_OC_ and fill factor on the work function of the cathode. Figure 22i shows the simulated band diagrams of the ETL‐free PSCs with the electrode's work function (4.4 and 3.7 eV), respectively. As shown in Figure 22h, both the *V*_OC_ and fill factor of the device tend to be saturated with the decrease of the metal work function, as expected by the metal–insulator–metal picture. In the case of an open circuit, as shown in Figure 22i, the applied voltage forms a flat‐band energy diagram in the active layer, and the difference in quasi‐Fermi energy level determines the *V*_OC_.^[^ ^26^ ^]^

Up to now, most of the device physics models of the ETL‐free PSCs are 1D models with well‐defined semiconductor layers. However, it is still challenging to define the unclear contact in the model due to the lack of understanding of physics. As a result, an overall the MHP/metal contacts model with consistent parameters is still lacking, making it almost impossible to simulate the interface and the interface engineering accurately. Compromise efforts have been made to simulate and fit the change in device performance caused by interface modification based on limited experimental results. In most cases, the simulation introduced the MHP/metal contact by changing the parameters and physical quantities of the functional layers connected to the MHP/metal contact. When the experiment found that contact engineering reduces the trap density, the modified device was modeled with a smaller interface trap density.^[^ ^180^, ^282^ ^]^ In other cases, Fermi level shifts of the contact metals and/or band bending of the semiconductor are constructed to represent the contact effects on the energy band.^[^ ^202^ ^]^ Sometimes, the device physics simulation fits the performance of the devices well with different interfaces by combining with the experimental results. This way of treating the contact from its effect strongly depends on the meaningful study of it by experiment. In other words, the device physics simulation only justifies the influences of these relevant experimental findings on the device performance to a certain extent. The fundamental understanding of the effects of the contact to the energy band and charge carrier processes is still not covered by device physics theory.

The device model simulation has achieved some basic instructions for the design of the ETL/HTL‐free PSCs. First, the energy level matching of the electrode and the MHP is always expected to increase the open‐circuit voltage. Second, in a given energy band, the reduction of carrier recombination can increase the quasi‐Fermi level splitting under the open‐circuit voltage condition and reduce the loss of open circuit voltage. Third, energy band alignment at the MHP/TL contact and band bending is beneficial to the transport and extraction of a carrier at the interface, which greatly influences the fill factor of the device. Indeed, device physics models the components of the device based on the material and physical parameters and analyzes and designs the device within the range of these parameters. In the past decade, many similar works have been done to reveal the working mechanism of PSCs, as well as to design the architecture.^[^ ^281^ ^]^ However, the device physics model has not realized a precise simulation of the ETL‐free PSCs. One of the primary reasons is the absence of an accurate understanding of the working mechanism for the MHP devices, especially for the MHP/electrode contact, leading to incomplete device physics models. Thus, there are still lots of experimental and theoretical works that should be done to enhance the understanding of the internal physical mechanism of ETL/HTL‐free PSCs in detail.

### 6.2 Modeling of Devices with Ion Migration and Charge Transfer

Most devices with MHP/electrode contacts exhibit memristive conductance and memristive photoconductivity. For ETL/HTL‐free PSCs, the device performance suffers from serious photocurrent–voltage hysteresis loops that depend on the voltage sweeping direction (from short‐circuit to open‐circuit and vice versa) and voltage sweeping rate. For the memristors, the memristive effect and resistance switching effects are present.

A coupled process of ion migration and charge carrier transport plays a vital role in explaining these behaviors. It was introduced into the physical modeling to reproduce the hysteresis curve of PSCs. Since devices with MHP/electrode contacts are more dependent on the historical state than traditional full‐structure PSCs, the coupled process of ion migration and charge carrier transport should be treated precisely.

**Figure 23:**
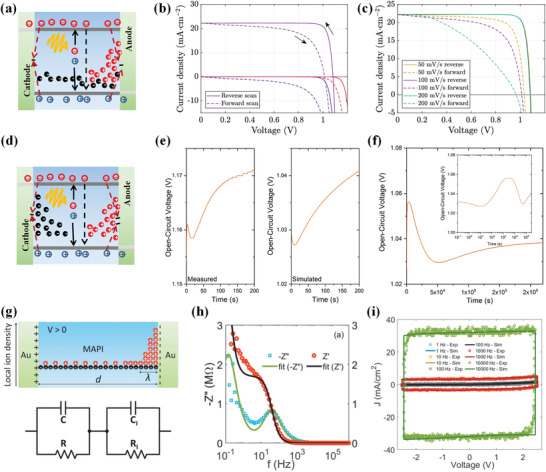
a) Schematics illustration of cations migration model. b) A simulated *J*–*V* curve at 100 mV s^−1^ from 1.2 V to short‐circuit and back, after a 5s preconditioning step. The purple lines show the current–density output, while the blue and red lines show the current losses due to interface recombination at the ETL/perovskite and perovskite/HTL interfaces, respectively. c) Simulated *J*–*V* curves with a set of three different scan rates. Reproduced under the terms of a Creative Commons Attribution 4.0 International License.^[^ ^283^ ^]^ Copyright 2019, The Author(s). Published by Springer Nature. d) Schematics illustration of migration of both anions and cations in interaction with trap‐mediated recombination in the bulk and/or at the surfaces. e) Experimental measurements and simulation of the *V*_OC_ transient following abrupt illumination from dark conditions for PSCs. f) The simulated long‐term transient voltage response of PSCs. The inset figure plots the transient response on a log(t) scale. Reproduced with permission.^[^ ^284^ ^]^ Copyright 2018, American Chemical Society. g) Schematics illustration on the ion movement in a biased Au/MAPbI_3_/Au device and equivalent circuit with *R*_I_ and *C*_I_ the resistance and capacitance of the accumulation layer, *R* and *C* are the resistance and capacitance of bulk MAPbI_3_. h) The measured and calculated real (*Z*′) and imaginary (*Z*″) part of the complex impedance *Z* as a function of frequency. i) Experimental (circles) and simulated (solid lines) current density–voltage characteristics of a MAPbI_3_ capacitor at different frequencies ranging from 1 Hz to 10 kHz. At low frequencies, the total current is small and dominated by the electronic current. At 10 kHz, the current is fully dominated by the displacement current. Reproduced with permission.^[^ ^285^ ^]^ Copyright 2020, Wiley‐VCH.

The obstacle of the numerical solution of the coupled equations of ion migration and charge carrier transport is the temporal and spatial stiffness. Courtier et al. developed a finite element scheme to solve this problem. They first applied the model to a single perovskite layer of PSCs and then extended it to multilayer PSCs.^[^ ^283^ ^]^ In Courtier's models, an equation was added to describe the effects of vacancy migration, as shown in **Figure** **23**a, which only considered the dynamics of the vacancy. As shown in Figure 23b,c, the model reproduced the current–density output with a hysteresis loop (purple lines) generally observed in PSCs. Moreover, the model gave a detailed interpretation of the origin of the hysteresis: the current losses because of the interface recombination regulated by ion distribution (the blue and red lines). However, whether the ion distribution regulates the recombination at the interface is still a problem. There are other mechanisms for the hysteresis loop. For example, it was assumed that the change of the internal electric field caused by ion distribution could affect the collection of carriers, eventually resulting in current–voltage hysteresis. Courtier's model just considered the vacancy dynamics and neglected the migration of I^−^ ions and their reaction with metals at the contact, which are limited in the simulation of devices with MHP/electrode contacts. Walter et al. have considered the motion of both anions and cations in their model, as shown in Figure 23d. And their solution to the model is based on Quokka3, a freely available software initially designed to model silicon‐based solar cells.^[^ ^284^ ^]^ The model replicated the nonmonotonic transients of multiple time constants (Figure 23e,f). Figure 23e shows the *V*_OC_ transient of 200 s obtained by experimental measurements and simulation on a MAPbI_3_ PSC with an abrupt illumination from dark conditions. The voltage transient showed approximately a residual decay up to 2000 s, which only one mobile ion model could reproduce. However, with prolonged time, the time constants of the decay should be predicted with an additional slower‐moving anion. Figure 23f shows the extended simulation of *V*_OC_ transient up to 10^6^ s, which implies that steady‐state for both anions and cations is not obtained until roughly 2 × 10^5^ s. Besides, the additional *V*_OC_ transient maxima and minima indicated a slow‐moving cation over hours. The work demonstrated that the MHP device is nonmonotonic but with multiple ion processes, and the slow‐moving species should be treated on a long‐time scale.

The electrical characteristics of the device in the frequency domain are instructive. The complex impedance *Z* as a function of frequency is usually measured and calculated. And the device, such as Au/MAPbI_3_/Au, is described by an equivalent circuit with *R*_I_ and *C*_I,_ the resistance and capacitance of the accumulation layer, respectively, as shown in Figure 23g. The measured and calculated real (*Z*′) and imaginary (*Z*″) parts of the complex impedance *Z* as a function of frequency were shown (see Figure 23h). The coincidence of the measured and calculated curves makes it possible to extract the features of ion migration from the experiment data. Experimental impedance data show that *Z*″ exhibits a minimum (*ω*_3_) and a maximum (*ω*_1_), as shown in the green line in Figure 23h.^[^ ^285^ ^]^ Alvar et al. obtained an ion diffusion coefficient of 1 × 10^−15^ m^2^ s^−1^ by using the frequency and assumed that the positively charged ion vacancies are mobile while ions keep immobile in the coupled ionic and electronic drift‐diffusion model. Figure 23i shows the *J*–*V* characteristics of the device at different frequencies ranging from 1 Hz to 10 kHz. At a low frequency of 1 Hz, the low electronic current is dominated, whereas the displacement current is dominated at the frequency of 10 kHz.^[^ ^285^ ^]^ The frequency‐domain electric properties indicate a way to study the process of ion motion, which separates the ion process from the electron and the hole carriers. The combination of the ion model and experimental measurement is expected to reveal more detailed information on the ion migration in devices with MHP/electrode contacts.

### 6.3 Modeling of MHP‐Based Memristors

Modeling the MHP‐based memristor is an essential aspect of developing emerging electronic devices. An accurate model is helpful for understanding the device operation, optimizing the performance, and designing the structure of the device to match specific requirements. The modeling of the MHP‐based memristor device is similar to traditional RRAM. Numerous physical models of traditional RRAM have been proposed according to different device types. Chua's model gave a basic theory for memristors.^[^ ^149^ ^]^ Panda et al. provided a comprehensive discussion and detailed information on various models proposed for the RRAM.^[^ ^290^ ^]^ However, compared with PSCs modeling, the simulation of MHP‐based memristors is still in its infancy, and there are very few related reports.

**Figure 24:**
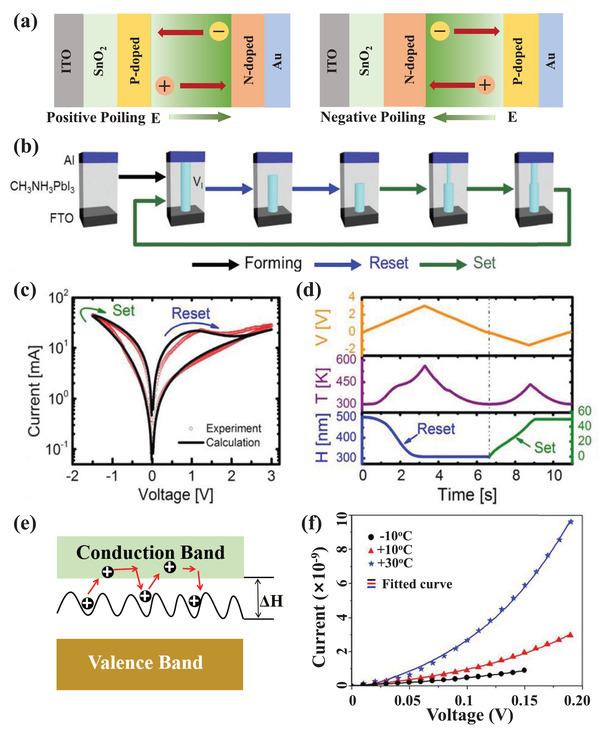
a) Schematics illustration of ion drift in MHP layer during voltage poling of device Glass/ITO/SnO_2_/MAPbI_3_/Au. b) Schematic illustration of conductive filament evolution during forming, reset and set processes in modeling resistive switching of Al/MAPbI_3_/FTO RRAM device. c) Measured and calculated *I*–*V* characteristics of the MHP‐based memory device evidencing set and reset transitions for negative and positive voltages, respectively. d) Time evolution of calculated applied voltage waveform (top), temperature profile (center), and conductive filament height and radius (bottom) during reset/set operation cycle. Reproduced with permission.^[^ ^288^ ^]^ Copyright 2018, Wiley‐VCH. e) Schematic illustration of electron movement in the thermally assisted hopping model. f) Fitting results for the *I*–*V* curves obtained experimentally at three different temperatures. Reproduced under the terms of a Creative Commons Attribution 4.0 International License.^[^ ^289^ ^]^ Copyright 2018, Author(s). Published by AIP Publishing LLC.

Generally, the modeling of an MHP‐based memristor needs to deal with two factors related to the metal interface: 1) coupling effect of the ion migration process and interface carrier process, and 2) diffusion of metal ions or ions species of the MHP and the formation of conductive filaments. Gupta et al. proposed a SPICE model to simulate MHP‐based synaptic memristor devices with Glass/ITO/SnO_2_/MAPbI_3_/Au structure.^[^ ^291^ ^]^ In their models, there are mainly two processes that need to be handled mathematically. 1) The time‐dependent drift of ions under the applied field in a dark condition. The drift of the ions produces a switching mechanism in the MHP layer for the positive poling, and negative poling, respectively, where accumulated charge carriers in the MHP layer near the electrodes induce p and n doping, as shown in **Figure** **24**a. 2) The switching mechanism of ion‐induced p‐ or n‐doping would increase or decrease the work function of the MHP surface, respectively, forming a dynamically regulated electron or hole injection barrier on cathode or anode. When the voltages swept between −1.5 and +1.5 V, the model successfully reproduces *I*–*V* curves with hysteresis behavior in dark conditions. Ren et al. attributed the resistive switching effect to the formation and rupture of *V*_I_ based conductive filament and showed an analytical model for resistive switching in MAPbI_3_‐based memristor.^[^ ^288^ ^]^ As shown in Figure 24b, the *V*_I_ migration‐based analytical model includes three operation phases: forming phase, reset phase, and set phase. During the formation process, a negative voltage promoted a high electric field, leading to the *V*_I_ nucleation followed by conductive filament formation in the MHP film, which enables the activation of the resistive switching for the following cycles. After forming, the application of a positive bias causes the *V*_I_ migration from the top electrode to the back electrode, leading to the formation of a depleted region near the top electrodes, referred to as a gap, and thus in a reset transition. Subsequently, a set transition induced by a negative bias at the top electrode allows the formation of the conductive filament again, thus supporting resistive switching cycling. The model successfully grasps the current and the voltage characteristics. Figure 24c shows the measured *I*–*V* characteristics of the MHP‐based memory with gradual set and reset transitions for negative and positive voltages, respectively. Figure 24d shows the time evolution of the calculated applied voltage waveform (top), temperature profile (center), and the conductive filament height and radius (bottom) during the reset/set operation cycle. Under the positive voltage scanning with a rate of 0.9 V s^−1^, the temperature and the electric field of conductive filament would increase until the voltage reaches *V*_reset_. By contrast, under a negative voltage, it would induce a set transition from the final state achieved by reset transition. During the set transition, *V*_I_ migration from the back electrode to the top electrode causes the formation of a subconductive filament in the gap region with a gradual increase in conductive filament radius.^[^ ^287^ ^]^

The previous models were usually limited to *I*–*V* curves measured at room temperature. To simulate the temperature‐dependent *I*–*V* curves, Park et al. demonstrated a thermally assisted hopping model as an appropriate ion‐transporting model for MHP‐based memories.^[^ ^289^ ^]^ As shown in Figure 24e, the thermally assisted hopping model employs a mechanism that describes the movement of individual ions through MHP materials. The ions transporting is highly dependent on the temperature because the ions obtain thermal energy of thermal fluctuations and phonon interaction to escape from their localized state and jump to an extended state and travel for a short time before being recaptured by another localized state. The *I*–*V* curves obtained at different temperatures are well fitted by the model, as shown in Figure 24f. They further proved that high trap density could help to realize a high ON/Off ratio in MHP‐based RRAM devices. Considering that the resistance of the device in the set state is dependent on the conductive filament rather than the trap density, the larger resistance from the traps in reset states would lead to the larger ON/OFF ratio.

For the resistive switching behaviors, the electrochemical metallization mechanism of the active metal ions is advocated. The mechanism involves an electrochemically active electrode metal, the metal ion conductive layer, and their metallization through discharge at the counter electrode, showing dissolution and establishment of conductive metal filament. However, an accurate description of the metal conductive filament dynamic process has not been established. It still needs a lot of work to uncover the elusive microscopic details.^[^ ^292^ ^]^ Comprehensive modeling of the resistive switching mechanism could help in solving the poor endurance and multicoefficient sensitivity that is often encountered in memristors.

## 7 Outlook and Perspectives

Despite recent progress in preparing devices with MHP/electrode contact, more in‐depth investigations about MHP/electrode contacts are still in demand. The currently available research in allusion to chemical reactions and physical processes around the MHP/electrode contacts also needs further study. It is desirable to develop new characterization techniques on MHP/electrode contacts, advanced experimental methods, interface modification techniques, contact physics, and models to assemble MHP/electrode contact appropriately and modify interface technology. Here, we provide some potential topics concerning MHP/electrode contact further to boost the overall performance of charge‐transporting‐layer free electronic devices.

1) Characterization techniques on MHP/electrode contact. More in situ characterization techniques are needed to obtain more information about the film morphology, chemical composition, and electronic structure at perovskite/electrode contacts. The time‐resolved and frequency‐resolved electrical characterization can be combined with the device's physical model to illustrate the charge‐carrier processes accurately. Time‐resolved and frequency‐resolved measurements, for example, impedance spectroscopy and transient photocurrent, can provide more information than steady‐state measurements due to their different response times to external electrical stimulation. Impedance spectroscopy in EIS measurements shows shoulders and peaks in the frequency range. Different electrical processes can be distinguished from the frequency impedance responses. Ions with low mobility are responsible for the low‐frequency peak that will vanish without mobile ions. Similarly, transfer, recombination or interface processes of more conductive electron/hole induce characteristics of impedance spectroscopy in the medium and high‐frequency regions. However, accurate modeling of carrier generation, transporting, and recombination are required in this method. The mutual promotion of the experiment and device physics not only helps to clarify the electrical mechanism for the charge‐transporting‐layer free electric devices but also promotes the establishment of the physical modeling as a complex system.
2) Modeling the device physics. The main factors that restrict the performance of ETL/HTL‐free PSCs should be clarified. Such devices approach the Shockley–Queisser limit by suppressing the carrier recombination may be possible. Currently, the PCEs for ETL/HTL‐free PSCs have exceeded 21%, and it is promising that the PCE can reach above 25%, whereas the PCEs of full PSC have exceeded 25%. The detailed and systematic power‐loss analysis of electrical and optical mechanisms of ETL/HTL‐free PSCs would be beneficial for constructing the physical model. Meanwhile, it is necessary to strengthen the systematic study of the combination of device physics and experiments on new photovoltaic devices. Moreover, device physics theory should cover the effects of the contact to the energy band and charge carrier processes. To this end, a fundamental understanding and detailed modeling of the MHP/metal contact are necessary.

Modeling the device physics process of MHP/electrode contacts is a challenge. As the device thickness ranges from hundreds of nanometers to a few micrometers, the interface characteristics greatly affect the performance of charge‐transporting‐layer free devices. The relevant physical simulations originate from crystalline silicon devices and organic thin‐film devices. And physical simulation methods have been developed by adding ion migration into the simulation process of PSCs in recent years. In spite of the previous impressive results, there are still some challenges to be solved for more accurate modeling in MHP/electrode contacts.

In the case of MHP‐based memristors, studying MHP/metal contact is crucial to developing the MHP memory devices and artificial synapses for massive data storage and low‐power neuromorphic computation. Up to now, there is no precise theory to describe the formation process of metal filaments and the process of ion migration and interface carrier coupling, which are responsible for plastic conduction and resistance switching. For the development of MHP/electrode‐related devices, in‐depth investigations of the ion transfer mechanism and interface characteristics of MHP/electrode, such as charge accumulation, band bending, and electronic structure would be needed. In addition, physical device models need to be further developed to precisely predict the performance of the devices with MHP/electrodes and better design such devices. The modeling of MHP‐based field‐effect transistors involving 3D modeling and simulation is relatively rare, which is a challenging direction in physical modeling and application.

3) Regulation of the complex coupling effect. The MHP/metal contacts are coupled with the migration of metal atoms and MHP ions to produce a resistant switching effect and plastic conduction, which is innovatively applied to massive data storage and mimics neuron function. Besides, the ion screening effect and charge carrier injection barrier variation due to the ion pervasion and accumulation at the contact spoil the performance of the MHP‐based field‐effect transistors at room temperature. The sensitivity of the MHP‐based device to ion migration and accumulation reduces the repeatability and stability of the related MHP‐based devices. It is essential to regulate the interaction between interface charge carrier dynamics and the processes of ion migration, diffusion, and accumulation to accurately control the performance of MHP‐based memories, artificial synapses, and field‐effect transistors. Interface modification and interface engineering can change the contact properties and electrical characteristics and provide methods for regulating the coupling effect at MHP/electrode contacts.
4) Interface Engineering. Introducing interfacial modification layers between MHP and electrode is a common strategy to avoid defects introduced by MHP/electrode contacts, such as carrier recombination, interface degradation, poor interface contact, and so on. We emphasize the importance of MHP/electrode interface engineering technology in constructing the related devices discussed in this review. First, interface engineering techniques should be developed to change the morphology, the wettability of the electrode, and the growth of MHP films. Second, the electrode modification can adjust the work function and realize the energy level matching between the electrode and the MHP film by introducing favorable electronic structures such as interface dipole layers, tunneling channels, and band bending at the interface. Compatible effective insulating layers are also needed to isolate MHP from the electrode and prevent MHP decomposition at the MHP/electrode contacts.
5) Developing new stable electrode materials. The choice of electrode materials constrains the development of devices with the MHP/electron contact. In addition to modifying existing electrodes, developing new electrodes would be a good research direction. In recent years, various C‐based electrodes have been used to prepare perovskite devices, exhibiting certain hydrophobicity and stability. However, PSCs using C‐based electrodes have not yet achieved competitive performance due to their low PCEs. It is still a critical challenge to enhance the conductivity of C‐based electrodes and realize the energy level matching between them and MHP.
6) The perovskite quantum dots (nanocrystals)/electrode contacts. Perovskite quantum dots, as an important low‐dimensional perovskite material, show great potential in optoelectronic devices, especially perovskite memristor. However, there are few reports on specific research and substantive information about the perovskite quantum dots (QDs) (or nanocrystals)/electrode interfaces. Here, perovskite QDs/metal contact are briefly discussed in the field of perovskite memristor. We concluded that perovskite QDs/metal contacts are preferred over bulk perovskites/contact in memristor for three reasons. First, the QDs perovskite films are usually prepared by spin‐coating closely packed QDs on top of the ITO pads at room temperature, which avoids the heating processes during the forming of the bulk perovskites. The high temperature for facilitating the nucleation and crystallization would introduce undesirable thermal stress to the underlying TCO substrate and reduce its electrical conductivity. Second, QDs/metal contact shows good crystallographic quality with sharp boundaries and low surface roughness, resulting in modified TCO/QDs and metal/QDs contacts.^[^ ^293^ ^]^ Third, the perovskites QDs would offer a great opportunity compared to their bulk counterpart by introducing new effects, such as high charge carrier mobility, the effect of the exciton, electrons trapping, and the inherent quantum confinement. It was reported that the junction of QDs with electrodes leads to the separation of the exciton in the interfaces, which serves as the basis for optically mediated charge trapping and electrically induced charge releasing in CsPbBr_3_.^[^ ^293^ ^]^ The maintenance capability of the trapped charge carriers and electrical erasing operation by the release of the trapped electrons is the basis of programming operation and the electric erasing operation in memory devices.^[^ ^262^ ^]^ By combining the electrochemical reduction of Ag electrode and in the body of QDs/QWs, a fast formation of Ag filament in the material was achieved, resulting in a drastic conductivity increase and improved electrical switching behavior in perovskite memristors. It is reported that the fast formation of Ag filament was assisted by the enhanced electron transport of the QDs/QWs.^[^ ^294^ ^]^ However, the uniqueness of the perovskite quantum dots (or nanocrystals)/electrode contacts needs to be revealed by further research.
7) Promising 2D/3D MHP/electrode contacts. The 2D/3D MHP/electrode junction offers promising developments in optoelectronic, simple‐structure MHP devices. However, the preparation technology is still a limitation of the broad application of this structure. In addition, the physical properties of 2D/3D MHP/electrode contact also have important theoretical and application values.

## 8 Conclusion

In conclusion, a systematic summary of the role of MHP/electrode contacts on the device performance is made. The charge‐transporting‐layer‐free devices with the MHP/electrodes, including the ETL/HTL‐free PSCs, artificial synapses, and field‐effect transistors, are summarized and discussed with a particular emphasis on the effect of the special contacts on the device physics. The band structure, charge carrier processes, interface chemistries, and problems of the MHP/TCO contacts and the MHP/metal contacts are present, respectively, with a focus on electrode engineering technologies in the device modification. The physicochemical, electronic, and morphological properties of various MHP/electrode contacts, as well as relevant engineering techniques, are provided. In addition, the modeling of the ETL/HTL‐free PSCs and MHP‐based memristor devices is also discussed. Finally, the current challenges are analyzed, and relevant recommendations are put forward to improve the device's performance further. It can be expected that further research will lead to significant breakthroughs in their application for future energy, memory, and computing technologies and promote reforms and innovations in future solid‐state physics and materials science.

## Conflict of Interest

The authors declare no conflict of interest.
